# Catalyst‐Driven Scaffold Diversity: Selective Synthesis of Spirocycles, Carbazoles and Quinolines from Indolyl Ynones

**DOI:** 10.1002/chem.201601836

**Published:** 2016-05-19

**Authors:** John T. R. Liddon, Michael J. James, Aimee K. Clarke, Peter O'Brien, Richard J. K. Taylor, William P. Unsworth

**Affiliations:** ^1^ University of YorkYorkYO24 4PPUK

**Keywords:** carbazoles, catalysis, diversity, quinolines, spirocycles

## Abstract

Medicinally relevant spirocyclic indolenines, carbazoles and quinolines can each be directly synthesised selectively from common indolyl ynone starting materials by catalyst variation. The high yielding, divergent reactions all proceed by an initial dearomatising spirocyclisation reaction to generate an intermediate vinyl–metal species, which then rearranges selectively by careful choice of catalyst and reaction conditions.

The synthesis of structurally diverse compounds is central to the discovery of pharmaceutical lead compounds.^1^ However, the formation of distinct compound sets usually requires multiple synthetic routes, which is time‐consuming and labour‐intensive; therefore, strategies capable of selectively forming multiple products from common starting materials are of high value. The concept underpinning our approach is the formation of a common reactive intermediate (from a simple, inexpensive starting material), which depending on the catalyst used can rearrange into different scaffolds (e.g., spirocycles, aromatics and heterocycles/carbocycles; Figure 1). This approach has the potential to significantly streamline existing synthetic methods, and lead to a broader understanding of catalysis and reaction mechanisms. Although there have been numerous examples of catalyst variation leading to different products in recent years,^2^, ^3^ such methods have mainly focused on the formation of products with similar frameworks (e.g., redox isomers, regioisomers or stereoisomers). In this work, our aim was to develop a series of divergent processes capable of selectively delivering multiple products with the level of scaffold diversity outlined in Figure 1.

**Figure 1:**
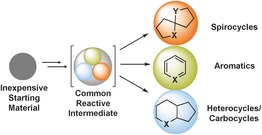
Catalyst‐driven scaffold diversity.

To demonstrate the synthetic potential of our scaffold‐diversity approach, we chose to explore the formation and subsequent reaction of spirocyclic vinyl–metal intermediates of the form **2** (Scheme 1). Previous work in our research group has demonstrated that the dearomatising spirocyclisation^4^ of ynones **1** into spirocyclic indolenines **3** can be catalysed by AgOTf, with vinyl–silver species **2** ([M]=Ag) as likely intermediates.^5^ A key design feature of our strategy was the idea that varying the catalyst would alter the nature and reactivity of the vinyl–metal intermediate **2** in a programmable way, such that alternative products could be formed by different rearrangement reactions. Herein, we report the successful realisation of this approach. Notably, by judicious choice of catalyst, simple, inexpensive ynone starting materials **1** can be converted into spirocyclic indolenines^6^ **3** using Ag^I^, carbazoles **5** using Au^I^ and quinolines **7** using Ag^I^/Al^III^ in high yield, each by a simple, catalytic and atom‐economical process. Furthermore, in suitable cases, tetracyclic scaffolds **8** can be formed with complete diastereoselectivity, by a telescoped spirocyclisation/nucleophilic addition sequence, which was performed using a chiral Ag^I^ salt to furnish an enantiopure product.

**Scheme 1:**
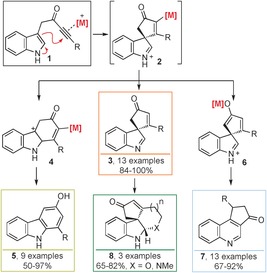
Divergent synthesis of spirocycles **3**, carbazoles **5**, quinolines **7** and tetracyclic scaffolds **8** from indolyl ynones **1**.

The spirocyclisation of **1 a** using AgOTf formed indolenine **3 a** in quantitative yield (Scheme 2);^5^ the mild reaction conditions are believed to play a key role in this process, stabilising the spirocycle with respect to further reactions. However, in the proposed scaffold diversity approach, in which the synthesis of carbazole **5 a** was an initial goal, the challenge was to deliberately promote 1,2‐migration^7^ in a controlled manner.^8^ A Ph_3_PAuNTf_2_ catalyst was chosen based on the prediction that the π‐acidic gold(I) catalyst would effectively promote the initial spirocyclisation reaction and that the intermediate vinyl–gold species (**2 a**‐**Au**) would be prone to 1,2‐migration, based on known reactivity of related vinyl–gold and gold–carbenoid species.^9^ This idea was validated (94 % yield of **5 a**) with a likely reaction mechanism depicted in Scheme 3; the ring enlargement is believed to proceed either via cyclopropane intermediate **9 a**, or by a direct 1,2‐migration reaction (**2 a**‐**Au→10 a**) based on related precedent.^7^, ^9^ The importance of vinyl–gold intermediate **2 a**‐**Au** in the 1,2‐migration is evidenced by the fact that no reaction takes place when spirocycle **3 a** is treated with Ph_3_PAuNTf_2_ under the same conditions.

**Scheme 2:**
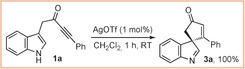
Formation of spirocyclic indolenine **3 a**.

**Scheme 3:**
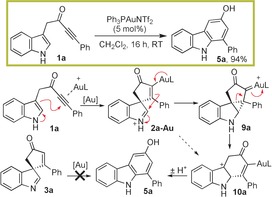
Formation of carbazole **5 a**; [Au]=Ph_3_PAuNTf_2_, L=ligand.

We next examined whether we could initiate an alternative rearrangement commencing from ynone **1 a**, by seeking to promote cyclopropanation of an enolate from the less substituted branch of the cyclopentenone; more oxophilic catalysts were chosen for this task, as it was thought that they would better promote the necessary enolate formation. We were unable to uncover a catalyst that could successfully initiate spirocyclisation and subsequent rearrangement on its own. However, first performing the spirocyclisation using 2 mol % of AgOTf as catalyst in isopropanol, followed by the addition of 5 mol % of AlCl_3_⋅6H_2_O and subsequent heating in a microwave gave quinoline **7 a** in high yield (Scheme 4).^10^ Following Ag^I^‐mediated spirocyclisation, it is thought that the Al^III^ catalyst promotes enolate formation and subsequent cyclopropanation to form **12 a**, which can then fragment to form **13 a** and aromatise to give quinoline **7 a** (either by simple proton shuttling, or by a series of 1,5‐sigmatropic H‐transfer reactions).

**Scheme 4:**
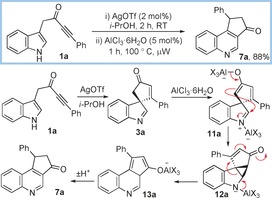
Formation of quinoline **7 a**; X=Cl or *i*PrO.

Supporting evidence for this unprecedented rearrangement was obtained: treatment of spirocycle **3 a** with LHMDS in THF (i.e. conditions which almost certainly would result in enolate formation) also led to the formation of quinoline **7 a**, in 81 % yield. Furthermore, the importance of the carbonyl group was shown by the fact that treatment of known cyclopentenol **14** 11 with AlCl_3_⋅6H_2_O did not result in quinoline formation. Instead, 1,2‐migration of the alkenyl group took place, furnishing carbazole **15** following tautomerisation and dehydration (Scheme 5).

**Scheme 5:**
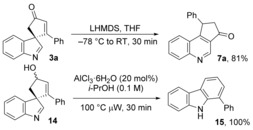
Base‐mediated formation of quinoline **7 a** and the contrasting reactivity of spirocyclic cyclopentenol **14**.

To probe the scope of all three reaction manifolds, various functionalised indole‐tethered ynones **1 a**–**1 m** were prepared, substituted in several positions with electron‐rich and ‐poor aromatics, alkyl substituents, *O*‐ and *N*‐protected alkyl groups and PhS.^12^ First, using the AgOTf‐mediated spirocyclisation methodology, substrates **1 a**–**1 m** were cleanly converted into the corresponding spirocyclic indolenines **3 a**–**3 m**, all in excellent yields (Table 1, conditions A). The Ph_3_PAuNTf_2_‐mediated carbazole‐forming reaction was similarly broad in scope (conditions B); some reactions were less efficient than the analogous spirocycle formations, and ynone **1 d** did not produce any of the desired product (instead stalling at the formation **3 d**), but the majority of the carbazole products **5 a**–**j** were isolated in very good yields.^13^ Finally, the quinoline‐forming reaction sequence was also found to be very general (conditions C). For ynones **1 a**–**1 e**,**1 g**,**1 k**–**1 l**, the sequential AgOTf spirocyclisation and AlCl_3_⋅6 H_2_O mediated rearrangement steps could both be performed in *i*PrOH in one‐pot as described, whereas for ynones with more sensitive functional groups (**1 f**, **1 h**, **1 i**, **1 j**, **1 m**), the process benefited from a solvent swap, with the spirocyclisation first being performed in CH_2_Cl_2_ before concentration and addition of *i*PrOH prior to the AlCl_3_⋅6 H_2_O step. The AlCl_3_⋅6 H_2_O reactions were typically performed under microwave irradiation at 100 °C, but they were also shown to proceed well on a gram scale with conventional heating, albeit with a longer reaction time being required.^14^ The structure of quinoline **7 f** was confirmed by X‐ray crystallography.^15^

**Table 1:** Reaction scope for the formation of spirocyclic indolenines, carbazoles and quinolones.

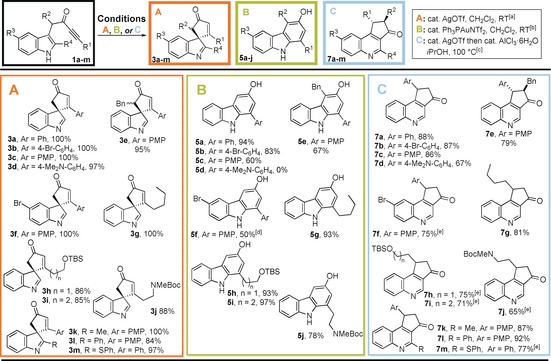

[a] AgOTf (1 mol %) in CH_2_Cl_2_ (0.1 m) at RT for 0.1–3.5 h. [b] Ph_3_PAuNTf_2_ (2–5 mol %) in CH_2_Cl_2_ (0.1 m) at RT for 7–18 h. [c] AgOTf (1 mol %) in *i*PrOH (0.1 m) at RT for 1–3 h, then AlCl_3_⋅6 H_2_O (5–10 mol %) at 100 °C μW for 1–2 h. [d] Reaction performed in toluene. [e] AgOTf (1 mol %) in CH_2_Cl_2_ (0.1 m) at RT for 1–3 h, then solvent swap for *i*PrOH (0.1 m) then AlCl_3_⋅6 H_2_O (5–10 mol %) at 100 °C μW for 1–2 h. PMP=*para‐*methoxyphenyl.

Another strand of scaffold diversity starting from more functionalised ynones **1 h**–**1 j** was briefly explored. Tetracyclic scaffolds **8 h**–**j**, equipped with additional complexity, were easily obtained following reaction of ynones **1 h**–**1 j** with AgOTf and subsequent acid‐mediated protecting group cleavage in one pot (Scheme 6, and see the Supporting Information for details).^16^ The tetracycles were formed as the single diastereoisomers shown, and in addition, **(*S*)‐8 h** was prepared in enantioenriched form (89:11 e.r.) by utilising (*R*)‐CPA silver(I) salt **16** in place of AgOTf.^17^ The e.r. of **(*S*)‐8 h** could be increased to ≈100:0 by recrystallisation from ethanol, and its structure was confirmed by X‐ray crystallography (see the Supporting Information).^15^

**Scheme 6:**
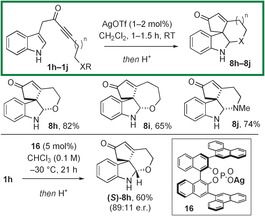
One‐pot spirocyclisation/trapping to form tetracycles **8 h**–**8 j**.

In summary, readily available indolyl ynones have been shown to be versatile starting materials for the synthesis of spirocyclic indolenines **3 a**–**m**, carbazoles **5 a**–**j**, quinolines **7 a**–**m** and tetracyclic compounds **8 h**–**j** using a catalyst‐driven scaffold diversity approach. The reactions are typically high yielding, work on a wide range of indolyl ynone substrates, are operationally simple and can all be performed with no effort to exclude air or moisture. All of the procedures are thought to proceed by an initial dearomatising spirocyclisation to form a key vinyl–metal intermediate before diverging at this point depending on the nature of the catalyst used. The synthetic methods are expected to be of value both in target synthesis projects^18^ and to enable the rapid generation of compound libraries for biological screening.

## Supporting information

As a service to our authors and readers, this journal provides supporting information supplied by the authors. Such materials are peer reviewed and may be re‐organized for online delivery, but are not copy‐edited or typeset. Technical support issues arising from supporting information (other than missing files) should be addressed to the authors.

Supplementary

## References

[1a] M. D. Burke , S. L. Schreiber , Angew. Chem. Int. Ed. 2004, 43, 46–58; 10.1002/anie.200300626 14694470

[1b] F. Lovering , J. Bikker , C. Humblet , J. Med. Chem. 2009, 52, 6752–6756; 19827778 10.1021/jm901241e

[1c] A. W. Hung , A. Ramek , Y. Wang , T. Kaya , J. A. Wilson , P. A. Clemons , D. W. Young , Proc. Natl. Acad. Sci. USA 2011, 108, 6799–6804; 21482811 10.1073/pnas.1015271108 PMC3084099

[1d] M. Aldeghi , S. Malhotra , D. L. Selwood , A. W. E. Chan , Chem. Biol. Drug Des. 2014, 83, 450–461; 24472495 10.1111/cbdd.12260 PMC4233953

[1e] A. Karawajczyk , F. Giordanetto , J. Benningshof , D. Hamza , T. Kalliokoski , K. Pouwer , R. Morgentin , A. Nelson , G. Muller , A. Pierchot , D. Tzalis , Drug Discovery Today 2015, 20, 1310–1316. 26429298 10.1016/j.drudis.2015.09.009

[2] For a review on ‘Catalytic Selective Synthesis’, see: J. Mahatthananchai , A. M. Dumas , J. W. Bode , Angew. Chem. Int. Ed. 2012, 51, 10954–10990; 10.1002/anie.201201787 23011639

[3] For more recent examples, see:

[3a] J. D. Dooley , S. Reddy Chidipudi , H. W. Lam , J. Am. Chem. Soc. 2013, 135, 10829–10836; 23806064 10.1021/ja404867k

[3b] D. S. B. Daniels , A. S. Jones , A. L. Thompson , R. S. Paton , E. A. Anderson , Angew. Chem. Int. Ed. 2014, 53, 1915–1920; 10.1002/anie.201309162 24505010

[3c] P. A. Donets , N. Cramer , Angew. Chem. Int. Ed. 2015, 54, 633–637; 10.1002/anie.201409669 25378295

[3d] Y.-S. Zhang , X.-Y. Tang , M. Shi , Org. Chem. Front. 2015, 2, 1516–1520;

[3e] L. Xu , H. Li , Z. Liao , K. Lou , H. Xie , H. Li , W. Wang , Org. Lett. 2015, 17, 3434–3437; 26146935 10.1021/acs.orglett.5b01435

[3f] J.-Y. Liao , P.-L. Shao , Y. Zhao , J. Am. Chem. Soc. 2015, 137, 628–631; 25555127 10.1021/ja511895q

[3g] A. Galván , J. Calleja , A. B. González-Pérez , R. Álvarez , A. R. de Lera , F. J. Fañanás , F. Rodríguez , Chem. Eur. J. 2015, 21, 16769–16774; 26440995 10.1002/chem.201503044

[3h] D. Y. Li , H. J. Chen , P. N. Liu , Angew. Chem. Int. Ed. 2016, 55, 373–377; 10.1002/anie.201508914 26531133

[3i] Q.-Q. Cheng , J. Yedoyan , H. Arman , M. P. Doyle , J. Am. Chem. Soc. 2016, 138, 44–47. 26699516 10.1021/jacs.5b10860

[4] For reviews on dearomatising spirocyclisation reactions, see:

[4a] C.-X. Zhuo , W. Zhang , S.-L. You , Angew. Chem. Int. Ed. 2012, 51, 12662; 10.1002/anie.201204822 23208999

[4b] S. P. Roche , J.-J. Y. Tendoung , T. Tréguier , Tetrahedron 2015, 71, 3549.

[5] M. J. James , J. D. Cuthbertson , P. O'Brien , R. J. K. Taylor , W. P. Unsworth , Angew. Chem. Int. Ed. 2015, 54, 7640–7643; 10.1002/anie.201501812 25960013

[6] For a review on spirocyclic indolenine synthesis, see: M. J. James , P. O'Brien , R. J. K. Taylor , W. P. Unsworth , Chem. Eur. J. 2016, 22, 2856–2881. 26661053 10.1002/chem.201503835

[7] For examples of related 1,2-migration reactions, see:

[7a] Q. F. Wu , C. Zheng , S.-L. You , Angew. Chem. Int. Ed. 2012, 51, 1680–1683; 10.1002/anie.201107677 22223488

[7b] V. A. Peshkov , O. P. Pereshivko , E. V. Van der Eycken , Adv. Synth. Catal. 2012, 354, 2841–2848;

[7c] C. Zheng , Q.-F. Wu , S.-L. You , J. Org. Chem. 2013, 78, 4357–4365; 23578142 10.1021/jo400365e

[7d] S. J. Heffernan , J. P. Tellam , M. E. Queru , A. C. Silvanus , D. Benito , M. F. Mahon , A. J. Hennessy , B. I. Andrews , D. R. Carbery , Adv. Synth. Catal. 2013, 355, 1149–1159.

[8] For other syntheses of carbazoles involving alkyne activation, see:

[8a] L. Wang , G. Li , Y. Liu , Org. Lett. 2011, 13, 3786–3789; 21732593 10.1021/ol2012154

[8b] A. S. K. Hashmi , W. Yang , F. Rominger , Chem. Eur. J. 2012, 18, 6576–6580; 22517669 10.1002/chem.201200314

[8c] Y. Qiu , C. Fu , X. Zhang , S. Ma , Chem. Eur. J. 2014, 20, 10314–10322. 25056816 10.1002/chem.201402423

[9a] C. Nieto-Oberhuber , M. P. Muñoz , E. Buñuel , C. Nevado , D. J. Cárdenas , A. M. Echavarren , Angew. Chem. Int. Ed. 2004, 43, 2402–2406; 10.1002/anie.200353207 15114573

[9b] C. Nieto-Oberhuber , S. López , M. P. Muñoz , E. Jiménez-Núñez , E. Buñuel , D. J. Cárdenas , A. M. Echavarren , Chem. Eur. J. 2006, 12, 1694–1702; 16358352 10.1002/chem.200501089

[9c] V. López-Carrillo , N. Huguet , Á. Mosquera , A. M. Echavarren , Chem. Eur. J. 2011, 17, 10972–10978; 22022709 10.1002/chem.201101749

[9d] K. Wittstein , K. Kumar , H. Waldmann , Angew. Chem. Int. Ed. 2011, 50, 9076–9080; 10.1002/anie.201103832 21866577

[9e] R. Meiss , K. Kumar , H. Waldmann , Chem. Eur. J. 2015, 21, 13526–13530; 26356499 10.1002/chem.201502843

[9f] A. Zhdanko , M. E. Maier , ACS Catal. 2015, 5, 5994–6004;

[9g] R. Dorel , A. M. Echavarren , Chem. Rev. 2015, 115, 9028–9072. 25844920 10.1021/cr500691k PMC4580024

[10] These reaction conditions were originally developed for the cleavage of silyl protecting groups, see: D. González-Calderón , L. J. Benitez-Puebla , C. A. Gonzalez-Gonzalez , M. A. Garcia-Eleno , A. Fuentes-Benitez , E. Cuevas-Yañez , D. Corona-Becerril , C. González-Romero , Synth. Commun. 2014, 44, 1258–1265.

[11] M. J. James , R. E. Clubley , K. Y. Palate , T. J. Procter , A. C. Wyton , P. O'Brien , R. J. K. Taylor , W. P. Unsworth , Org. Lett. 2015, 17, 4372. 26293968 10.1021/acs.orglett.5b02216

[12] Ynones **1 a**–**g** and **1 k**–**l** were prepared as described in reference [5], while ynones **1 h**–**j** and **1 m** were synthesised for the first time in this work (see Supporting Information for preparative details).

[13] For related carbazole syntheses, see: J. Wang , H.-T. Zhu , Y.-F. Qiu , Y. Niu , S. Chen , Y.-X. Li , X.-Y. Liu , Y.-M. Liang , Org. Lett. 2015, 17, 3186–3189, and references [8a–c]. 26061269 10.1021/acs.orglett.5b01590

[14] Ynone **1 a** (3.8 mmol) was converted into quinoline **7 a** in 82 % yield upon conventional heating with AlCl_3_⋅6 H_2_O at reflux under an air atmosphere for 8 h (see the Supporting Information for full details).

[15] CCDC 1453490 (**7 f**) and 1453491 (**(*S*)-8 h**) contain the supplementary crystallographic data for this paper. These data are provided free of charge by The Cambridge Crystallographic Data Centre.

[16] For a related reaction involving in situ indolenine formation and intramolecular trapping, see: S. G. Modha , A. Kumar , D. D. Vachhani , J. Jacobs , S. K. Sharma , V. S. Parmar , L. van Meervelt , E. V. van der Eycken , Angew. Chem. Int. Ed. 2012, 51, 9572–9575; 10.1002/anie.201205052 22907654

[17a] G. L. Hamilton , E. J. Kang , M. Mba , F. D. Toste , Science 2007, 317, 496–499; 17656720 10.1126/science.1145229 PMC13591957

[17b] Y. Wang , K. Zheng , R. Hong , J. Am. Chem. Soc. 2012, 134, 4096–4099; 22329839 10.1021/ja300453u

[17c] M. Terada , F. Li , Y. Toda , Angew. Chem. Int. Ed. 2014, 53, 235–239; 10.1002/anie.201307371 24273203

[18] For details of potential natural product targets, see:

[18a] P. Magnus , B. Mugrage , M. R. Deluca , G. A. Cain , J. Am. Chem. Soc. 1990, 112, 5220–5230;

[18b] T. S. Kam , K. M. Sim , T. M. Lim , Tetrahedron Lett. 2001, 42, 4721–4723;

[18c] M. Mori , M. Nakanishi , D. Kajishima , Y. Sato , J. Am. Chem. Soc. 2003, 125, 9801–9807; 12904045 10.1021/ja029382u

[18d] D. Crich , S. Rumthao , Tetrahedron 2004, 60, 1513–1516;

[18e] H.-J. Knölker , W. Fröhner , R. Heinrich , Synlett 2004, 2705–2708;

[18f] H. Yang , J. Feng , Y. Tang , Chem. Commun. 2013, 49, 6442–6444; 10.1039/c3cc42686f 23756469

[18g] W. P. Unsworth , J. D. Cuthbertson , R. J. K. Taylor , Org. Lett. 2013, 15, 3306–3309; 23786419 10.1021/ol4013958

[18h] N. Ramkumar , R. Nagarajan , J. Org. Chem. 2013, 78, 2802–2807; 23421392 10.1021/jo302821v

[18i] Y. Hieda , T. Choshi , H. Fujioka , S. Hibino , Eur. J. Org. Chem. 2013, 7391–7401.

