# Supplementary material for: Catalyst‐Driven Scaffold Diversity: Selective Synthesis of Spirocycles, Carbazoles and Quinolines from Indolyl Ynones
Source: Chemistry. 2016 May 19;22(26):8777–80. doi: 10.1002/chem.201601836 (PMC5084754; doi:10.1002/chem.201601836)
Supplement: Supplementary file 1 — Supplementary [file CHEM-22-8777-s001.pdf]

CHEMISTRY
A European Journal

Supporting Information

Catalyst-Driven Scaffold Diversity: Selective Synthesis of Spirocycles, Carbazoles and Quinolines from Indolyl Ynones

John T. R. Liddon, Michael J. James, Aimee K. Clarke, Peter O'Brien, Richard J. K. Taylor,* and William P. Unsworth*[a]

chem_201601836_sm_miscellaneous_information.pdf

Table of Contents **Page**

Table of Contents	Page
General information	2
General procedures	3
Characterisation data and procedures	5
Optimisation tables for quinoline rearrangement	21
^{1}H and ^{13}C NMR spectra	22
HPLC data	57
X-ray Crystal Images	58
References	59

General Information

Except where stated, all reagents were purchased from commercial sources and used without further purification. Anhydrous CH_2Cl_2, toluene and DMF were obtained from an Innovative Technology Inc. PureSolv® solvent purification system. Anhydrous THF was obtained by distillation over sodium benzophenone immediately before use. Microwave reactions were conducted on a CEM Discover-S microwave reactor, Model 908860. ^{1}H NMR and ^{13}C NMR spectra were recorded on a JEOL ECX400 or JEOL ECS400 spectrometer, operating at 400 MHz and 100 MHz, respectively. All spectral data was acquired at 295 K. Chemical shifts (δ) are quoted in parts per million (ppm). The residual solvent peak, δ_H 7.25 and δ_C 77.0 for $CDCl_3$, δ_H 2.50 and δ_C 39.5 for d6-DMSO was used as a reference. Coupling constants (*J*) are reported in Hertz (Hz) to the nearest 0.5 Hz. The multiplicity abbreviations used are: s singlet, d doublet, t triplet, q quartet, pent pentet, sext sextet, m multiplet. Signal assignment was achieved by analysis of DEPT, COSY, HMBC and HSQC experiments where required. Infrared (IR) spectra were recorded on a PerkinElmer UATR 2 spectrometer as a thin film dispersed from either CH_2Cl_2 or $CDCl_3$. Mass-spectra (low and high-resolution) were obtained by the University of York Mass Spectrometry Service, using electrospray ionisation (ESI) on a Bruker Daltonics, Micro-tof spectrometer. Melting points were determined using Gallenkamp apparatus. Thin layer chromatography was carried out on Merck silica gel $60F_{254}$ pre-coated aluminium foil sheets and were visualised using UV light (254 nm) and stained with basic aqueous potassium permanganate. Flash column chromatography was carried out using slurry packed Fluka silica gel (SiO_2), 35–70 µm, 60 Å, under a light positive pressure, eluting with the specified solvent system. Petrol refers to petroleum ether 40–60 °C.

Compounds **S1**, **1a–1g, 1k–1l**, **3a–3g, 3k–3l**,and **16** prepared according to literature.[1] **S6** prepared according to literature.[2]

General procedure A: Ynone synthesis

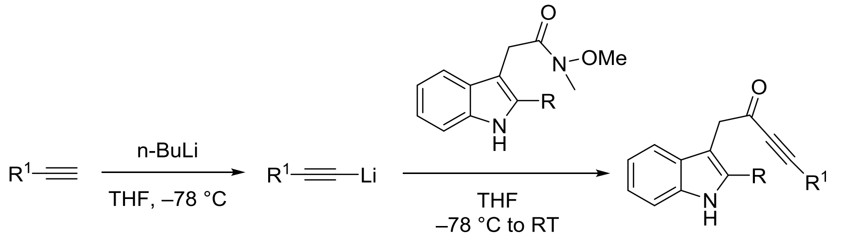

Amide: **S1** - R = H
S5 - R = SPh

To an oven-dried round-bottom flask containing the relevant alkyne (3 eq.) in THF (1 mL/ mmol) at –78 °C under argon was added n-BuLi (2.5 eq.) dropwise. After stirring at –78 °C for 1 h, the alkyne solution was transferred via cannula into an oven-dried round-bottom flask containing the relevant amide (**S1** or **S5**) (1 eq.) in THF at –78 °C. After stirring for 1 h, the resulting suspension was warmed to RT, during which time it became homogenous - indicating the end of the reaction. The reaction was quenched with saturated aqueous NH_4Cl, extracted successively with EtOAc (3 x), and the combined organics washed with brine, dried, and concentrated by vacuum, to yield a crude product. The product was purified by column chromatography (silica) eluting with 10–50% EtOAc in hexane, yielding the desired ynone compound and recovered alkyne where relevant.

General procedure B: Spirocyclisation

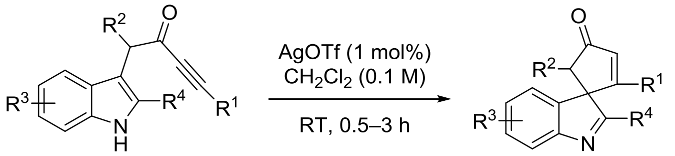

To a 0.1 M CH_2Cl_2 solution of the relevant ynone compound was added AgOTf (1–2 mol%) and the reaction stirred under air at RT. After completion as judged by TLC (0.5–3 h), the reaction was concentrated by vacuum and purified by column chromatography (silica) eluting with 20% to 50% EtOAc in hexane, affording the desired spirocycle.

General procedure C: Rearrangement to carbazoles

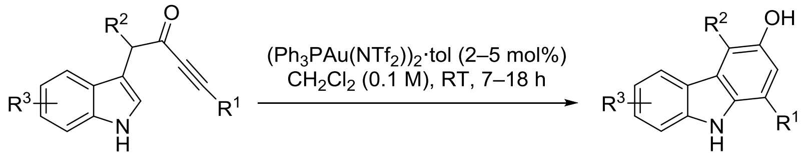

To a 0.1 M CH_2Cl_2 solution of the relevant ynone compound was added $(Ph_3PAu(NTf_2))_2$·tol (2–5 mol%) and the reaction stirred under air at RT until complete as judged by TLC (7–18 h). The reaction was concentrated by vacuum and purified by column chromatography (silica) eluting with 20–50% EtOAc in hexane, affording the desired carbazole compound.

General procedure D: One-pot rearrangement to quinoline with one solvent

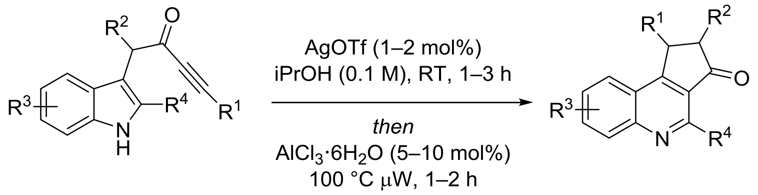

To a 2–5 mL Biotage microwave reaction tube was added a 0.1 M isopropanol solution of the relevant ynone compound, AgOTf (1–2 mol%), and the reaction stirred under air at RT. After completion as judged by TLC (1–3 h), $AlCl_3{\cdot}6H_2O$ (5–10 mol%) was added, and the reaction heated using a microwave under an air atmosphere for at 100 °C until completion (1–2 h). The reaction solution was concentrated by vacuum and purified by column chromatography (silica), eluting with 20–50% EtOAc in hexane, yielding the desired quinoline compound.

General procedure E: One-pot rearrangement to quinoline with solvent swap

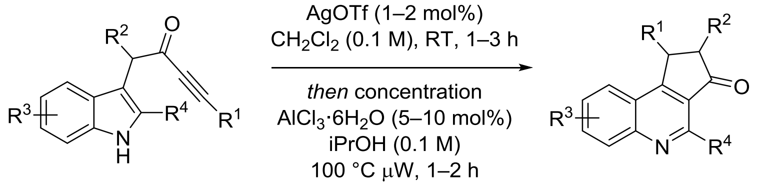

To a 2–5 mL Biotage microwave reaction tube was added a 0.1 M CH_2Cl_2 solution of the relevant ynone compound, AgOTf (1–2 mol%), and the reaction stirred under air at RT. After completion as judged by TLC (0.5–2 h), the reaction was concentrated and the solvent swapped for isopropanol (0.1 M). $AlCl_3{\cdot}6H_2O$ (5–10 mol%) was added and the reaction heated using a microwave under an air atmosphere at 100 °C until completion (1–2 h). The reaction solution was concentrated by vacuum and purified by column chromatography (silica), eluting with 20–50% EtOAc in hexane, yielding the desired quinoline compound.

(But-3-yn-1-yloxy)(tert-butyl)dimethylsilane (S2)

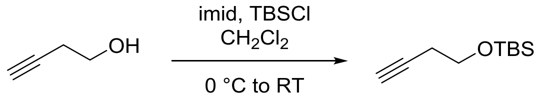

A literature procedure was used.[3,4] To a 250 mL round-bottom flask containing imidazole (4.00 g, 58.7 mmol), 3-butyn-1-ol (3.00 mL, 40.0 mmol), and CH_2Cl_2 (30 mL) at 0 °C under argon was added TBSCl (6.50 g, 43.6 mmol). The reaction was warmed to RT, stirred for 2 h, then filtered through a plug of silica and washed with EtOAc. The filtrate was concentrated and purified by column chromatography (silica), eluting with 10% EtOAc in hexane, affording the title compound as a colourless oil (7.68 g, 100%) with data consistent with the literature data.[3,4] R_f 0.82 (20% EtOAc in hexane); δ_H (400 MHz, $CDCl_3$) 0.06 (6H, s, Me_2Si), 0.89 (9H, s, *t*-BuSi), 1.94–1.96 (1H, m), 2.37–2.41 (2H, m), 3.73 (2H, dt, $^3J_{HH}$ 7.0, $^4J_{HH}$ 1.5).

tert-Butyldimethyl(pent-4-yn-1-yloxy)silane (S3)

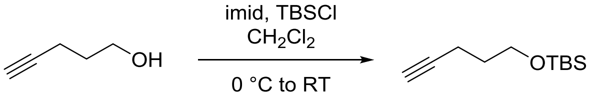

A modified literature procedure was used.[3,4] To a 100 mL round-bottom flask containing imidazole (1.60 g, 24.2 mmol), 4-pentyn-1-ol (1.50 mL, 16.1 mmol), and CH_2Cl_2 (15 mL) at 0 °C under argon was added TBSCl (2.70 g, 17.8 mmol). The reaction was warmed to RT, stirred for 1 h, then filtered through a plug of silica and washed with EtOAc. The filtrate was concentrated and purified by column chromatography (silica), eluting with 10% EtOAc in hexane, affording the title compound as a colourless oil (3.20 g, 100%) with data consistent with the literature data.[5] R_f 0.66 (25% EtOAc in hexane); δ_H (400 MHz, $CDCl_3$) 0.06 (6H, s, Me_2Si), 0.89 (9H, s, *t*-BuSi), 1.73 (2H, app. pent, $^3J_{HH}$ 6.5), 1.93 (1H, t, $^4J_{HH}$ 2.5), 2.27 (2H, dt, $^3J_{HH}$ 7.0, $^4J_{HH}$ 2.5, 3.70 (2H, t, $^3J_{HH}$ 6.0).

tert-Butyl but-3-yn-1-yl(methyl)carbamate (S4)

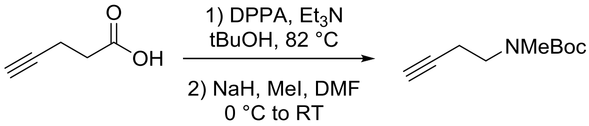

A modified literature procedure was used.[6] To a 100 mL round-bottom flask containing pentynoic acid (908 mg, 9.25 mmol), DPPA (2.60 mL, 12.0 mmol), and *t*BuOH (9 mL) was added triethylamine (1.70 mL, 12.0 mmol) and the reaction heated at reflux for 16 h. The reaction was removed from heat, concentrated, and diluted with EtOAc (80 mL). The solution was washed successively with saturated aqueous NH_4Cl (40 mL), saturated aqueous $NaHCO_3$ (40 mL), brine (40 mL), dried ($MgSO_4$), and concentrated to yield a crude oil that was purified by column chromatography (silica) eluting with 10% EtOAc in hexane. The resulting amine was dissolved in DMF (8 mL) and treated with sodium hydride (554 mg, 60% in mineral oil, 13.9 mmol) at 0°C under argon. After 10 min, methyl iodide was added (0.92 mL, 14.8 mmol) and the reaction warmed to RT. After 1 h, the reaction was quenched with saturated aqueous $NaHCO_3$ (100 mL), extracted with EtOAc (100 mL), then washed with brine (50 mL). The combined aqueous washes were back-extracted with EtOAc (20 mL), then the combined organics dried ($MgSO_4$), concentrated, and purified by column chromatography (silica) eluting with 10% to 20% EtOAc in hexane, yielding the title compound as a colourless oil (894 mg, 50%). R_f 0.80 (50% EtOAc in hexane); ν_{max} (thin film)/cm^{-1} 3308 (w), 3249 (w), 2976 (w), 1690 (s), 1479 (m), 1391 (s), 1365 (s), 1141 (s); δ_H at 55 °C (400 MHz, $CDCl_3$) 1.45 (9H, s), 1.93 (1H, t, $^4J_{HH}$ 3.0)

2.39 (2H, dt, $^3J_{HH}$ 7.0, $^4J_{HH}$ 3.0), 2.89 (3H, s), 3.36 (2H, t, $^3J_{HH}$ 7.0). δ_C at 55 °C (100 MHz, $CDCl_3$) 18.0 (CH_2), 28.5 ($(CH_3)_3$), 34.9 (CH_3), 48.0 (CH_2), 69.5 (CH), 79.6 (C), 81.8 (C), 155.5 (C=O); HRMS (ESI$^+$): Found: 206.1153; $C_{10}H_{17}NNaO_2$ (MNa^+) Requires 206.1151 (–0.6 ppm error).

***N*-Methoxy-*N*-methyl-2-(2-(phenylthio)-1*H*-indol-3-yl)acetamide (S5)**

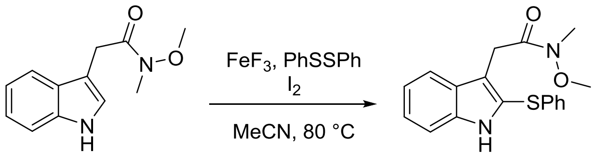

Synthesised according to a modified literature procedure.[7] Weinreb amide **S1** (1.09 g, 5.00 mmol), FeF_3 (113 mg, 1.00 mmol), Ph_2S_2 (546 mg, 2.50 mmol) and iodine (13 mg, 0.05 mmol) were combined in MeCN (38 mL) and the mixture was stirred for 4 days at 80 °C. The mixture was diluted with EtOAc (40 mL) and washed with saturated aqueous $Na_2S_2O_3$ (5 × 10 mL). The aqueous was extracted with EtOAc (2 × 50 mL) and the organics were combined, dried ($MgSO_4$) and concentrated under vacuum. The crude material was purified by column chromatography (CH_2Cl_2) then triturated with EtOAc to afford the title compound as an off-white solid (710 mg, 44%); R_f 0.40 (50% EtOAc in hexane); mp 175–176 °C; ν_{max} (thin film)/cm^{-1} 3266, 1648, 1478, 1439, 1340, 1000, 741, 690; δ_H (400 MHz, $CDCl_3$) 3.18 (3H, s), 3.67 (3H, s), 4.06 (2H, s), 7.09–7.18 (4H, m), 7.19–7.30 (4H, m), 7.72 (1H, d, $^3J_{HH}$ = 8.0 Hz), 8.21 (1H, br s); δ_C (100 MHz, $CDCl_3$) 29.3 (CH_2), 32.3 (CH_3), 61.2 (CH_3), 110.9 (CH), 116.9 (C), 120.0 (CH), 120.1 (CH), 123.3 (C), 123.5 (CH), 125.9 (CH), 127.0 (2 x CH), 127.9 (C), 129.1 (2 x CH), 136.6 (C), 136.9 (C), 171.8 (C); HRMS (ESI$^+$): Found: 349.0978; $C_{18}H_{18}N_2NaO_2S$ (MNa^+) Requires 349.0981 (0.8 ppm error), Found: 327.1160; $C_{18}H_{19}N_2O_2S$ (MH^+) Requires 327.1162 (0.6 ppm error).

5-((tert-Butyldimethylsilyl)oxy)-1-(1*H*-indol-3-yl)pent-3-yn-2-one (1h)

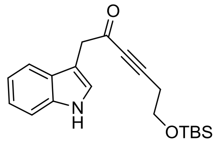

Synthesised using general procedure A with (but-3-yn-1-yloxy)(tert-butyl)dimethylsilane **S2** (7.00 g, 38 mmol), THF (45 + 30 mL), Weinreb amide **S1** (2.76 g, 12.7 mmol), n-BuLi (12.6 mL, 31.0 mmol, 2.5 M in hexanes). Purification by silica column chromatography (10–30% EtOAc in hexane) afforded the title compound as an orange oil (3.46 g, 80%); R_f 0.36 (25% EtOAc in hexane); ν_{max} (thin film)/cm^{-1} 3409 (br, NH), 2212 (m, C≡C), 1667 (s, C=O), 1253 (s), 1098 (s), 738 (s); δ_H (400 MHz, $CDCl_3$) 0.07 (6H, s, Me_2Si), 0.90 (9H, s, *t*-BuSi), 2.50 (2H, t, $^3J_{HH}$ 7.0), 3.67 (2H, t, $^3J_{HH}$ 7.0), 3.98 (2H, s), 7.08 (1H, d, $^5J_{HH}$ 2.5), 7.13 (1H, app. t, $^3J_{HH}$ 7.0), 7.20 (1H, app. t, $^3J_{HH}$ 7.0), 7.33 (1H, d, $^3J_{HH}$ 8.5), 7.58 (1H, d, $^3J_{HH}$ 7.5), 8.26 (1H, br, NH); δ_C (100 MHz, $CDCl_3$) –5.2 ($Si(CH_3)_2$), 18.4 (SiC), 23.5 (CH_2), 25.9 (Si*t*-Bu), 42.1 (CH_2), 60.8 (CH_2), 81.8 (C), 92.5 (C), 107.4 (C), 111.4 (CH), 118.9 (CH), 119.8 (CH), 122.3 (CH), 123.8 (CH), 127.3 (C), 136.2 (C), 185.7 (C=O); HRMS (ESI$^+$): Found: 364.1697; $C_{20}H_{27}NNaO_2Si$ (MNa^+) Requires 364.1703 (1.8 ppm error), Found: 342.1875; $C_{20}H_{28}NO_2Si$ (MH^+) Requires 342.1884 (2.7 ppm error).

7-((tert-Butyldimethylsilyl)oxy)-1-(1*H*-indol-3-yl)hept-3-yn-2-one (1i)

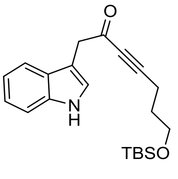

Synthesised using general procedure A with tert-butyldimethyl(pent-4-yn-1-yloxy)silane **S3** (2.10 g, 10.6 mmol), THF (15 + 15 mL), Weinreb amide **S1** (770 mg, 3.50 mmol), n-BuLi (3.50 mL, 8.8 mmol, 2.5 M in hexanes). Purification by silica column chromatography (10–30% EtOAc in hexane) afforded the title compound as an orange oil (1.03 g, 82%); R_f 0.42 (25% EtOAc in hexane); ν_{max} (thin film)/cm^{-1} 3410 (br, NH), 2210 (m, C≡C), 1665 (s, C=O), 1253 (s), 1099 (s), 832 (s), 738 (s); δ_H (400 MHz, $CDCl_3$) 0.07 (6H, s, Me_2Si), 0.90 (9H, s, *t*-BuSi), 1.64 (2H, app. pent, $^3J_{HH}$ 6.5), 2.38 (2H, t, $^3J_{HH}$ 7.5), 3.56 (2H, t, $^3J_{HH}$ 6.0), 3.97 (2H, s), 7.08 (1H, d, $^5J_{HH}$ 2.5), 7.13 (1H, app. t, $^3J_{HH}$ 7.0), 7.20 (1H, app. t, $^3J_{HH}$ 7.0), 7.33 (1H, d, $^3J_{HH}$ 8.5), 7.58 (1H, d, $^3J_{HH}$ 7.5), 8.27 (1H, br, NH); δ_C (100 MHz, $CDCl_3$) –5.3 ($Si(CH_3)_2$), 15.6 (CH_2), 18.4 (SiC), 26.0 (Si*t*-Bu), 30.7 (CH_2), 42.2 (CH_2), 61.2 (CH_2), 81.1 (C), 95.5 (C), 107.6 (C), 111.4 (CH), 118.9 (CH), 119.8 (CH), 122.3 (CH), 123.8 (CH), 127.5 (C), 136.2 (C), 185.9 (C=O); HRMS (ESI^+): Found: 378.1848; $C_{21}H_{29}NNaO_2Si$ (MNa^+) Requires 378.1860 (3.1 ppm error), Found: 356.2029; $C_{21}H_{30}NO_2Si$ (MH^+) Requires 356.2040 (3.1 ppm error).

tert-Butyl (6-(1*H*-indol-3-yl)-5-oxohex-3-yn-1-yl)(methyl)carbamate (1j)

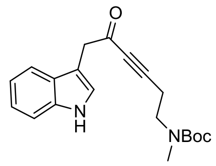

Synthesised using general procedure A with tert-butyl but-3-yn-1-yl(methyl)carbamate **S4** (1.11 g, 6.1 mmol), THF (6 + 5 mL), Weinreb amide **S1** (335 mg, 2.00 mmol), n-BuLi (2.00 mL, 5.00 mmol, 2.5 M in hexanes). Purification by silica column chromatography (10–30% EtOAc in hexane) afforded the title compound as an orange oil (700 mg, 85%); R_f 0.62 (50% EtOAc in hexane); ν_{max} (thin film)/cm^{-1} 3317 (br, NH), 2211 (m, C≡C), 1666 (s, C=O), 1143 (s), 738 (s); *Note: ^{1}H–NMR featured broad signals due to rotamers.* δ_H (400 MHz, $CDCl_3$) 1.44 (9H, s), 2.44–2.52 (2H, br m), 2.75 (3H, s), 3.21–3.31 (2H, br m), 3.94 (2H, s) 7.10–7.13 (2H, m), 7.56 (1H, t, $^3J_{HH}$ 7.5), 7.33 (1H, d, $^3J_{HH}$ 8.5), 7.57 (1H, d, $^3J_{HH}$ 8.5), 8.43 (1H, br, NH); *Note: ^{13}C–NMR featured broad signals due to rotamers.* δ_C (100 MHz, $CDCl_3$) 18.8 (CH_2), 28.5 ($(CH_3)_3$), 34.9 (CH_3), 42.1 (CH_2), 47.2 (CH_2), 80.1 (C), 81.7 (C), 92.0 (C), 92.6 (C), 107.3 (C), 111.4 (CH), 118.8 (CH), 119.8 (CH), 120.5 (C), 122.3 (CH), 123.9 (CH), 127.4 (CH), 130.0 (C), 136.2 (C), 155.4 (C=O), 185.6 (C=O); HRMS (ESI^+): Found: 363.1690; $C_{20}H_{24}N_2NaO_3$ (MNa^+) Requires 363.1679 (–2.9 ppm error).

4-Phenyl-1-(2-(phenylthio)-1*H*-indol-3-yl)but-3-yn-2-one (1m)

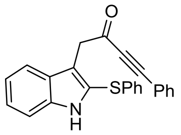

Synthesised using general procedure A with phenylacetylene (0.25 mL, 2.30 mmol), THF (2.5 + 7 mL), Weinreb **S5** (250 mg, 0.766 mmol), and n-BuLi (0.77 mL, 1.92 mmol, 2.5 M in hexanes). Purification by silica column chromatography (20% EtOAc in hexane) afforded the title compound as a yellow oil (224 mg, 80%); R_f 0.80 (50% EtOAc in hexane); ν_{max} (thin film)/cm^{-1} 3391, 3058, 2200, 1656, 1443, 1078, 738, 687; δ_H (400 MHz, $CDCl_3$) 4.24 (2H, s), 7.10–7.24 (6H, m), 7.25–7.38 (6H, m), 7.39–7.45 (1H, m), 7.68 (1H, br d, *J* = 8.0 Hz), 8.33 (1H, br s); δ_C (100 MHz, $CDCl_3$) 41.7 (CH_2) , 87.9 (C), 92.3 (C), 111.1 (CH), 115.2 (C), 119.6 (CH), 119.8 (C), 120.4 (CH), 123.8 (CH), 124.4 (C), 126.1 (CH), 127.1 (2 x CH), 127.7 (C), 128.4 (2 x CH), 129.1 (2CH), 130.6 (CH), 133.1 (2 x CH), 136.2 (C), 136.9 (C), 184.8 (C=O); HRMS (ESI^+): Found: 390.0907; $C_{24}H_{17}NNaOS$ (MNa^+) Requires 390.0929 (5.6 ppm error), Found: 368.1087; $C_{24}H_{17}NOS$ (MH^+) Requires 368.1109 (6.0 ppm error).

2-(2-((tert-Butyldimethylsilyl)oxy)ethyl)spiro[cyclopentane-1,3'-indol]-2-en-4-one (3h)

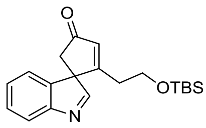

Synthesised using general procedure B with 6-((tert-butyldimethylsilyl)oxy)-1-(1*H*-indol-3-yl)hex-3-yn-2-one **1h** (1.00 g, 2.90 mmol), CH_2Cl_2 (29 mL), AgOTf (7.5 mg, 0.03 mmol) at RT for 1.5 h. Purification by column silica chromatography (20–50% EtOAc in hexane) afforded the title compound as an orange oil (858 mg, 86%); R_f 0.58 (50% EtOAc in hexane); ν_{max} (thin film)/cm^{-1} 2856 (w), 1719 (s, C=O), 1614 (m, C=N), 1252 (m), 1094 (s), 832 (s); δ_H (400 MHz, $CDCl_3$) –0.05 (3H, s, MeSi), –0.04 (3H, s, MeSi), 0.83 (9H, s, *t*-BuSi), 1.82–1.94 (2H, m), 2.63 (1H, d, $^2J_{HH}$ 18.5), 2.90 (1H, d, $^2J_{HH}$ 18.5), 3.56 (2H, t, $^3J_{HH}$ 6.0), 6.40 (1H, s), 7.21 (1H, d, $^3J_{HH}$ 7.0), 7.29 (1H, t, $^3J_{HH}$ 7.5), 7.41 (1H, t, $^3J_{HH}$ 6.0), 7.69 (1H, d, $^3J_{HH}$ 7.5), 7.96 (1H, s); δ_C (100 MHz, $CDCl_3$) –5.4 ($Si(CH_3)_2$), 18.2 (SiC), 25.9 (Si*t*-Bu), 32.1 (CH_2), 40.2 (CH_2), 60.5 (CH_2), 67.9 (C), 121.8 (CH x 2), 127.4 (CH), 129.2 (CH), 132.5 (CH), 139.2 (C), 155.8 (C), 173.2 (CH), 176.8 (C), 205.8 (C=O); HRMS (ESI^+): Found: 364.1695; $C_{20}H_{27}NNaO_2Si$ (MNa^+) Requires 364.1703 (2.4 ppm error), Found: 342.1875; $C_{20}H_{28}NO_2Si$ (MH^+) Requires 342.1884 (2.5 ppm error).

2-(3-((tert-Butyldimethylsilyl)oxy)propyl)spiro[cyclopentane-1,3'-indol]-2-en-4-one (3i)

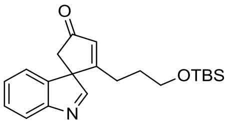

Synthesised using general procedure B with 7-((tert-butyldimethylsilyl)oxy)-1-(1*H*-indol-3-yl)hept-3-yn-2-one **1i** (310 mg, 0.870 mmol), CH_2Cl_2 (8.7 mL), AgOTf (2.2 mg, 0.009 mmol) at RT for 1 h. Purification by silica column chromatography (20–50% EtOAc in hexane) afforded the title compound as an off-white solid (266 mg, 85%); Mp 79–81 °C; R_f 0.55 (50% EtOAc in hexane); ν_{max} (thin film)/cm^{-1} 2951 (w), 1715 (s, C=O), 1690 (s), 1612 (m, C=N), 1254 (m), 1100 (s), 832 (s); δ_H (400 MHz, $CDCl_3$) –0.09 (3H, s, MeSi), –0.08 (3H, s, MeSi), 0.77 (9H, s, *t*-BuSi), 1.50–1.65 (2H, m), 1.74–1.79 (2H, m), 2.64 (1H, d, $^2J_{HH}$ 18.5), 2.91 (1H, d, $^2J_{HH}$ 18.5), 3.44 (2H, t, $^3J_{HH}$ 6.0), 6.29 (1H, s), 7.19 (1H, d, $^3J_{HH}$ 7.5), 7.29 (1H, t, $^3J_{HH}$ 7.5), 7.41 (1H, t, $^3J_{HH}$ 7.5), 7.68 (1H, d, $^3J_{HH}$ 7.5), 7.94 (1H, s); δ_C (100 MHz, $CDCl_3$) –5.4 ($Si(CH_3)_2$), 18.2 (SiC), 25.3 (CH_2), 25.9 (Si*t*-Bu), 30.5 (CH_2), 40.5 (CH_2), 61.8 (CH_2), 67.8 (C), 121.6 (CH), 121.8 (CH), 127.4 (CH), 129.1 (CH), 131.1 (CH), 139.5 (C), 155.8 (C), 173.2 (CH), 180.1 (C), 205.7 (C=O); HRMS (ESI^+): Found: 378.1845; $C_{21}H_{29}NNaO_2Si$ (MNa^+) Requires 378.1860 (3.9 ppm error), Found: 356.2026; $C_{21}H_{30}NO_2Si$ (MH^+) Requires 356.2040 (4.0 ppm error).

tert-Butyl methyl(2-(4-oxospiro[cyclopentane-1,3'-indol]-2-en-2-yl)ethyl)carbamate (3j)

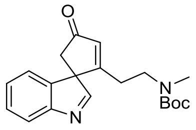

Synthesised using general procedure B with tert-butyl (6-(1*H*-indol-3-yl)-5-oxohex-3-yn-1-yl)(methyl)carbamate **1j** (215 mg, 0.63 mmol), CH_2Cl_2 (6.3 mL), AgOTf (3.2 mg, 0.012 mmol) at RT for 1 h. Purification by silica column chromatography (20–60% EtOAc in hexane) afforded the title compound as an orange oil (190 mg, 88%); R_f 0.28 (50% EtOAc in hexane); ν_{max} (thin film)/cm^{-1} 2975 (w), 1720 (s, C=O), 1691 (s, C=O), 1613 (m, C=N), 1392 (m), 1166 (s), 761 (s); *Note: ^1H–NMR featured broad signals due to rotamers.* δ_H (400 MHz, $CDCl_3$) 1.38 (9H, s), 1.80–1.94 (2H, m), 2.57–2.7 (4H, m),

2.91 (1H, d, $^2J_{HH}$ 19.0), 3.16–3.35 (2H, m), 6.29 (1H, s), 7.18–7.24 (1H, m), 7.31 (1H, t, $^3J_{HH}$ 7.5), 7.42 (1H, t, $^3J_{HH}$ 7.5), 7.70 (1H, d, $^3J_{HH}$ 7.5), 7.95 (0.5H, br), 8.09 (0.5H, br); *Note: ^{13}C–NMR featured broad signals due to rotamers.* δ_C (100 MHz, $CDCl_3$) 26.7 (CH_2), 28.4 (tBu), 34.0 (CH_3), 40.2 (CH_3), 46.5 & 47.3 (CH_2), 67.9 (C), 80.0 (C), 121.7 (CH), 121.8 (CH), 127.8 (CH), 127.8 (CH), 129.3 (CH), 139.1 (C), 155.8 (C=O), 172.7 & 176.2 (C), 176.2 (C), 205.4 (C=O); HRMS (ESI$^+$): Found: 363.1675; $C_{20}H_{24}N_2NaO_3$ (MNa$^+$) Requires 363.1679 (1.3 ppm error).

2-Phenyl-2'-(phenylthio)spiro[cyclopent[2]ene-1,3'-indol]-4-one (3m)

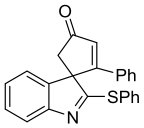

Synthesised using general procedure B with 4-phenyl-1-(2-(phenylthio)-1*H*-indol-3-yl)but-3-yn-2-one **1m** (69 mg, 0.188 mmol), AgOTf (0.5 mg, 0.002 mmol) in CH_2Cl_2 (1.8 mL) at RT for 0.5 h. Purification by column chromatography (30% EtOAc in hexane) afforded the title compound as a white solid (67 mg, 97%); R_f 0.66 (50% EtOAc in hexane); mp 177–179 °C; ν_{max} (thin film)/cm^{-1} 3063, 1694, 1509, 1447, 1261, 952, 762, 747, 728, 686; δ_H (400 MHz, $CDCl_3$) 2.85 (1H, d, *J* = 19.0 Hz), 3.20 (1H, d, *J* = 19.0 Hz), 6.87 (1H, s), 7.08–7.13 (2H, m), 7.13–7.20 (2H, m), 7.22–7.29 (2H, m), 7.32–7.39 (2H, m), 7.39–7.46 (3H, m), 7.50–7.57 (3H, m); δ_C (100 MHz, $CDCl_3$) 48.0 (CH_2) , 67.3 (C), 120.1 (CH), 121.2 (CH), 125.7 (CH), 127.0 (C), 127.2 (CH), 128.8 (CH), 128.9 (CH), 129.3 (CH), 129.5 (CH), 130.9 (CH), 131.2 (CH), 132.1 (C), 134.3 (CH), 141.5 (C), 154.5 (C), 171.8 (C), 182.9 (C), 204.5 (C=O); HRMS (ESI$^+$): Found: 390.0923; $C_{24}H_{17}NNaOS$ (MNa$^+$) Requires 390.0923 (0 ppm error), Found: 368.1105; $C_{24}H_{18}NOS$ (MH$^+$) Requires 368.1104 (−0.4 ppm error).

1-Phenyl-9*H*-carbazol-3-ol (5a)

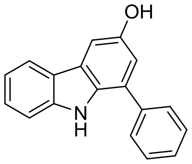

Synthesised using general procedure C with 1-(1*H*-indol-3-yl)-4-phenylbut-3-yn-2-one **1a** (93 mg, 0.35 mmol), $(Ph_3PAu(NTf_2))_2$·tol (12 mg, 0.009 mmol), CH_2Cl_2 (3.5 mL), at RT for 16 h. Purification by silica column chromatography (20–30% EtOAc in hexane) afforded the title compound as an orange solid (88 mg, 94%); Mp 60–65 °C; R_f 0.32 (25% EtOAc in hexane); ν_{max} (thin film)/cm^{-1} 3417 (br), 3055 (s), 1595 (s), 1488 (m), 1450 (m), 1411 (s), 1151 (s), 746 (s); δ_H (400 MHz, $CDCl_3$) 4.88 (1H, br), 7.01 (1H, d, $^4J_{HH}$ 3.0), 7.20 (1H, app. t, $^3J_{HH}$ 6.5), 7.35–7.45 (3H, m), 7.49 (1H, d, $^4J_{HH}$ 3.5), 7.53 (1H, t, $^3J_{HH}$ 7.5), 7.64–7.66 (2H, m), 8.0 (1H, d, $^3J_{HH}$ 8.5), 8.13 (1H, br); δ_C (100 MHz, $CDCl_3$) 105.1 (CH), 110.9 (CH), 114.7 (CH), 119.3 (CH), 120.6 (CH), 123.3 (C), 124.6 (C), 125.8 (C), 126.2 (CH), 127.8 (CH), 238.4 (2 x CH), 129.4 (2 x CH), 132.4 (C), 138.7 (C), 140.4 (C), 149.7 (C); HRMS (ESI$^+$): Found: 282.0890; $C_{18}H_{13}NNaO$ (MNa$^+$) Requires 282.0889 (–0.3 ppm error), Found: 260.1062; $C_{18}H_{14}NO$ (MH$^+$) Requires 260.1070 (3.0 ppm error).

1-(4-Bromophenyl)-9*H*-carbazol-3-ol (5b)

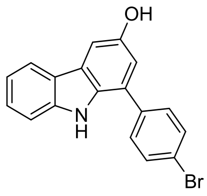

Synthesised using general procedure C with 4-(4-bromophenyl)-1-(1*H*-indol-3-yl)but-3-yn-2-one **1b** (60 mg, 0.18 mmol), $(Ph_3PAu(NTf_2))_2$·tol (7 mg, 0.004 mmol), CH_2Cl_2 (1.7 mL), at RT for 16 h. Purification by silica column chromatography (20–30% EtOAc in hexane) afforded the title compound as an orange solid (50 mg, 83%); Mp 185–187 °C; R_f 0.70 (50% EtOAc in hexane); ν_{max} (thin film)/cm^{-1} 3459 (m), 3186 (br), 3033 (w), 1625 (m), 1482 (s), 1237 (s), 1172 (s), 815 (s), 746 (s); δ_H (400 MHz, DMSO-d$_6$) 6.86 (1H, d, $^4J_{HH}$ 2.5), 7.05 (1H, t, $^3J_{HH}$ 7.0), 7.29 (1H, t, $^3J_{HH}$ 7.0), 7.40 (1H, d, $^3J_{HH}$ 8.5), 7.44 (1H, d, $^4J_{HH}$ 2.5), 7.59 (2H, d, $^3J_{HH}$ 8.5), 7.71 (2H, d, $^3J_{HH}$ 8.5), 7.98 (1H, d, $^3J_{HH}$ 7.5), 9.09 (1H, s), 10.75 (1H, s); δ_C (100 MHz, DMSO-d$_6$) 105.3 (CH), 111.9 (CH), 115.0 (CH), 118.6 (CH), 120.7 (C), 121.1 (CH), 122.8 (C), 124.3 (C), 124.7 (C), 126.0 (CH), 131.0 (2 x CH), 131.5 (C), 132.4 (2 x CH), 138.3 (C), 141.5 (C), 151.4 (C); HRMS (ESI$^+$): Found: 359.9965; $C_{18}H_{12}{}^{79}BrNNaO$ (MNa$^+$) Requires 359.9994 (8.3 ppm error).

1-(4-Methoxyphenyl)-9*H*-carbazol-3-ol (5c)

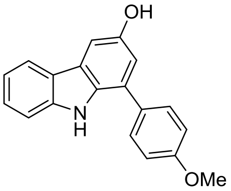

Synthesised using general procedure C with 1-(1*H*-indol-3-yl)-4-(4-methoxyphenyl)but-3-yn-2-one **1c** (74 mg, 0.25 mmol), $(Ph_3PAu(NTf_2))_2$·tol (10 mg, 0.006 mmol), CH_2Cl_2 (1.7 mL), at RT for 18 h. Purification by silica column chromatography (20–30% EtOAc in hexane) afforded the title compound as an orange solid (45 mg, 60%); Mp 133–138 °C; R_f 0.40 (33% EtOAc in hexane); ν_{max} (thin film)/cm^{-1} 3564 (m), 3335 (s), 1605 (m), 1501 (s), 1233 (s), 1148 (s), 1016 (s), 742 (s); δ_H (400 MHz, $CDCl_3$) 3.88 (3H, s), 4.82 (1H, s), 6.96 (1H, d, $^4J_{HH}$ 3.0), 7.06 (2H, d, $^3J_{HH}$ 9.0), 7.19 (1H, ddd, $^3J_{HH}$ 7.5, $^3J_{HH}$ 6.0, $^4J_{HH}$ 1.5), 7.35–7.41 (2H, m), 7.46 (1H, d, $^4J_{HH}$ 2.5), 7.58 (2H, d, $^3J_{HH}$ 8.5), 8.00 (1H, d, $^3J_{HH}$ 8.5), 8.09 (1H, s); δ_C (100 MHz, $CDCl_3$) 55.5 (CH_3), 104.5 (CH), 110.9 (CH), 114.5 (CH), 114.8 (2 x CH), 119.2 (CH), 120.6 (CH), 123.4 (C), 124.5 (CH), 125.6 (C), 126.1 (C), 129.5 (2 x CH), 131.0 (C), 132.5 (C), 140.4 (C), 149.7 (C), 159.3 (C); HRMS (ESI$^+$): Found: 312.0990; $C_{19}H_{15}NNaO_2$ (MNa$^+$) Requires 312.0995 (1.7 ppm error), Found: 290.1162; $C_{19}H_{16}NO_2$ (MH$^+$) Requires 290.1176 (4.5 ppm error).

4-Benzyl-1-(4-methoxyphenyl)-9*H*-carbazol-3-ol (5e)

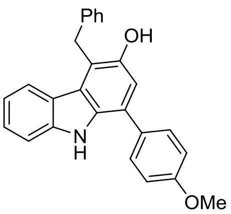

Synthesised using general procedure C with 4-(1*H*-indol-3-yl)-1-(4-methoxyphenyl)-5-phenylpent-1-yn-3-one **1e** (100 mg, 0.260 mmol), $(Ph_3PAu(NTf_2))_2$·tol (10 mg, 0.006 mmol), CH_2Cl_2 (2.6 mL), at RT for 18 h. Purification by silica column chromatography (20–30% EtOAc in hexane) afforded the title compound as a yellow solid (67 mg, 67%), as well as spirocycle **3e** (32 mg, 32%); Mp 62–68 °C; R_f

0.50 (33% EtOAc in hexane); ν_{max} (thin film)/cm^{-1} 3412 (br), 2931 (w), 1608 (m), 1505 (m), 1243 (s), 1174 (m), 832 (s), 735 (s); δ_H (400 MHz, $CDCl_3$) 3.88 (3H, s), 4.62 (1H, s), 4.70 (2H, s), 6.98 (1H, s), 7.05–7.13 (3H, m), 7.18 (1H, d, $^3J_{HH}$ 7.0), 7.24–7.28 (2H, m), 7.33–7.37 (4H, m), 7.58 (2H, d, $^3J_{HH}$ 9.0), 8.07 (1H, d, $^3J_{HH}$ 8.5), 8.18 (1H, s); δ_C (100 MHz, $CDCl_3$) 32.5 (CH_2), 55.5 (CH_3), 110.8 (CH), 114.8 (2 x CH), 115.1 (CH), 119.3 (CH), 122.6 (CH), 123.4 (C), 123.6 (C), 123.7 (C), 125.6 (CH), 126.3 (2 x CH), 128.3 (2 x CH), 128.8 (2 x CH), 129.6 (C), 131.0 (C), 132.8 (C), 139.5 (C), 140.5 (C), 147.8 (C), 159.2 (C); HRMS (ESI$^+$): Found: 418.1194; $C_{26}H_{21}NKO_2$ (MK^+) Requires 418.1204 (2.3 ppm error), Found: 402.1453; $C_{26}H_{21}NNaO_2$ (MNa^+) Requires 402.1465 (2.9 ppm error), Found: 380.1634; $C_{26}H_{22}NO_2$ (MH^+) Requires 380.1645 (3.0 ppm error).

6-Bromo-1-(4-methoxyphenyl)-9*H*-carbazol-3-ol (5f)

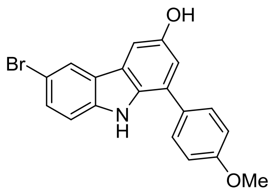

Synthesised using general procedure C with 1-(5-bromo-1*H*-indol-3-yl)-4-(4-methoxyphenyl)but-3-yn-2-one **1f** (50 mg, 0.13 mmol), $(Ph_3PAu(NTf_2))_2$·tol (5.3 mg, 0.003 mmol), toluene (1.4 mL), at RT for 16 h. Purification by silica column chromatography (20–30% EtOAc in hexane) afforded the title compound as an yellow solid (25 mg, 50%); Mp 70–75 °C; R_f 0.42 (33% EtOAc in hexane); ν_{max} (thin film)/cm^{-1} 3350 (br), 2929 (w), 2835 (w), 1607 (m), 1517 (m), 1451 (s), 1172 (s), 1236 (s), 1161 (s), 829 (s); δ_H (400 MHz, $CDCl_3$) 3.88 (3H, s), 4.96 (1H, br), 6.98 (1H, d, $^4J_{HH}$ 2.5), 7.05 (2H, d, $^3J_{HH}$ 8.5), 7.23 (1H, d, $^3J_{HH}$ 8.5), 7.37 (1H, d, $^4J_{HH}$ 2.5), 7.44 (1H, dd, $^3J_{HH}$ 8.5, $^4J_{HH}$ 1.5), 7.55 (2H, d, $^3J_{HH}$ 8.5), 8.08–8.11 (2H, m); δ_C (100 MHz, $CDCl_3$) 55.5 (CH_3), 104.5 (CH), 111.9 (C), 112.3 (CH), 114.8 (2 x CH), 115.4 (CH), 123.3 (CH), 123.4 (C), 125.1 (C), 125.9 (C), 128.7 (CH), 129.4 (2 x CH), 130.6 (C), 132.9 (C), 138.9 (C), 149.9 (C), 159.4 (C); HRMS (ESI$^+$): Found: 390.0073; $C_{19}H_{14}{}^{79}BrNNaO_2$ (MNa^+) Requires 390.0100 (6.9 ppm error).

1-Butyl-9*H*-carbazol-3-ol (5g)

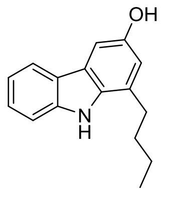

Synthesised using general procedure C with 1-(1*H*-indol-3-yl)oct-3-yn-2-one **1g** (60 mg, 0.25 mmol) $(Ph_3PAu(NTf_2))_2$·tol (4 mg, 0.002 mmol), CH_2Cl_2 (2.5 mL), at RT for 15 h. Purification by silica column chromatography (20–30% EtOAc in hexane) afforded the title compound as an off-white solid (56 mg, 93%); Mp 131–135 °C; R_f 0.68 (50% EtOAc in hexane); ν_{max} (thin film)/cm^{-1} 3458 (m), 3411 (s), 2952 (m), 2868 (m), 1599 (m), 1498 (s), 1436 (s), 1163 (s), 736 (s); δ_H (400 MHz, $CDCl_3$) 0.96 (3H, t, $^3J_{HH}$ 7.0), 1.44 (2H, app. sext, $^3J_{HH}$ 7.0), 1.74 (2H, app. pent, $^3J_{HH}$ 7.5), 2.83 (2H, t, $^3J_{HH}$ 8.0), 4.70 (1H, br), 6.81 (1H, d, $^4J_{HH}$ 1.5), 7.17 (1H, t, $^3J_{HH}$ 7.0), 7.33 (1H, d, $^4J_{HH}$ 1.5), 7.36–7.43 (3H, m), 7.83 (1H, br), 7.96 (1H, d, $^3J_{HH}$ 7.5); δ_C (100 MHz, $CDCl_3$) 14.1 (CH_3), 22.7 (CH_2), 31.2 (CH_2), 31.7 (CH_2), 103.2 (CH), 110.9 (CH), 114.7 (CH), 119.1 (CH), 120.5 (CH), 123.6 (C), 123.8 (C), 125.8 (CH), 125.9 (C), 133.5 (C), 140.2 (C), 149.4 (C); HRMS (ESI$^+$): Found: 262.1204; $C_{16}H_{17}NNaO$ (MNa^+) Requires 262.1202 (–0.7 ppm error), Found: 240.1379; $C_{16}H_{18}NO$ (MH^+) Requires 240.1383 (1.7 ppm error).

1-(2-((tert-Butyldimethylsilyl)oxy)ethyl)-9*H*-carbazol-3-ol (5h)

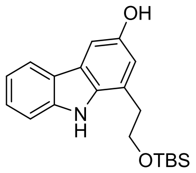

Synthesised using general procedure C with 6-((tert-butyldimethylsilyl)oxy)-1-(1*H*-indol-3-yl)hex-3-yn-2-one **1h** (135 mg, 0.395 mmol), $(Ph_3PAu(NTf_2))_2$·tol (6.2 mg, 0.004 mmol), CH_2Cl_2 (3.9 mL), at RT for 7 h. Purification by silica column chromatography (20–30% EtOAc in hexane) afforded the title compound as an off-white solid (126 mg, 93%); Mp 81–84 °C; R_f 0.78 (25% EtOAc in hexane); ν_{max} (thin film)/cm^{-1} 3337 (br), 2952 (m), 2928 (m), 2856 (m), 1627 (w), 1600 (w), 1502 (m), 1255 (s), 834 (s); δ_H (400 MHz, $CDCl_3$) –0.01 (6H, s), 0.90 (9H, s), 3.08 (2H, t, $^3J_{HH}$ 5.5), 4.00 (2H, t, $^3J_{HH}$ 5.5), 4.89–5.00 (1H, br), 6.77 (1H, d, $^4J_{HH}$ 2.5), 7.16 (1H, ddd, $^3J_{HH}$ 7.5, $^3J_{HH}$ 5.0, $^4J_{HH}$ 2.5), 7.35–7.37 (3H, m), 7.95 (1H, d, $^3J_{HH}$ 8.5), 9.02 (1H, s); δ_C (100 MHz, $CDCl_3$) –5.43 ($Si(CH_3)_2$), 18.5 (SiC), 26.1 ($(CH_3)_3$), 36.5 (CH_2), 64.7 (CH_2), 103.8 (CH), 111.1 (CH), 115.7 (CH), 118.8 (CH), 120.5 (CH), 123.6 (C), 123.9 (C), 124.2 (C), 125.8 (CH), 134.9 (C), 140.6 (C), 149.1 (C); HRMS (ESI$^+$): Found: 364.1698; $C_{20}H_{27}NNaO_2Si$ (MNa^+) Requires 364.1703 (1.5 ppm error), Found: 342.1879 $C_{20}H_{28}NO_2Si$ (MH^+) Requires 342.1884 (1.5 ppm error).

1-(3-((tert-Butyldimethylsilyl)oxy)propyl)-9*H*-carbazol-3-ol (5i)

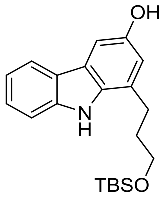

Synthesised using general procedure C with 7-((tert-butyldimethylsilyl)oxy)-1-(1*H*-indol-3-yl)hept-3-yn-2-one **1i** (68 mg, 0.191 mmol), $(Ph_3PAu(NTf_2))_2$·tol (3 mg, 0.002 mmol), CH_2Cl_2 (1.9 mL), at RT for 13 h. Purification by silica column chromatography (20–30% EtOAc in hexane) afforded the title compound as an off-white solid (66 mg, 97%); Mp 94–97 °C; R_f 0.62 (33% EtOAc in hexane); ν_{max} (thin film)/cm^{-1} 3277 (br), 2950 (m), 2927 (m), 1627 (w), 1599 (w), 1507 (m), 1250 (s), 983 (s), 829 (s); δ_H (400 MHz, $CDCl_3$) 0.17 (6H, s), 1.03 (9H, s), 1.91 (2H, app. pent, $^3J_{HH}$ 6.0), 2.99 (2H, t, $^3J_{HH}$ 6.0), 3.65 (2H, t, $^3J_{HH}$ 6.0), 4.86 (1H, s), 6.80 (1H, d, $^4J_{HH}$ 2.5), 7.16 (1H, ddd, $^3J_{HH}$ 7.5, $^3J_{HH}$ 5.0, $^4J_{HH}$ 3.0), 7.35 (1H, d, $^4J_{HH}$ 2.5), 7.36–7.38 (2H, m), 7.95 (1H, d, $^3J_{HH}$ 7.5), 9.06 (1H, s); δ_C (100 MHz, $CDCl_3$) –4.98 ($Si(CH_3)_2$), 18.5 (SiC), 26.2 ($(CH_3)_3$), 27.1 (CH_2), 33.3 (CH_2), 61.5 (CH_2), 103.5 (CH), 111.0 (CH), 115.2 (CH), 118.8 (CH), 120.5 (CH), 123.6 (C), 123.7 (C), 125.5 (CH), 125.7 (C), 134.9 (C), 140.6 (C), 149.1 (C); HRMS (ESI$^+$): Found: 378.1848; $C_{21}H_{29}NNaO_2Si$ (MNa^+) Requires 378.1860 (3.2 ppm error), Found: 356.2034 $C_{21}H_{30}NO_2Si$ (MH^+) Requires 356.2040 (1.7 ppm error).

tert-Butyl (2-(3-hydroxy-9*H*-carbazol-1-yl)ethyl)(methyl)carbamate (5j)

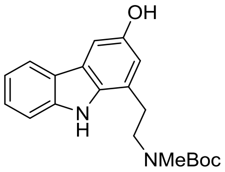

Synthesised using general procedure C with tert-butyl (6-(1*H*-indol-3-yl)-5-oxohex-3-yn-1-yl)(methyl)carbamate **1j** (120 mg, 0.352 mmol), $(Ph_3PAu(NTf_2))_2$·tol (5.5 mg, 0.003 mmol), CH_2Cl_2 (3.5

mL), at RT for 7 h. Purification by silica column chromatography (20–50% EtOAc in hexane) afforded the title compound as a pale pink solid (94 mg, 78%); Mp 71–75 °C; R_f 0.60 (50% EtOAc in hexane); ν_{max} (thin film)/cm^{-1} 3301 (br), 2928 (w), 1659 (s), 1482 (m), 1452 (m), 1365 (m), 1164 (s), 734 (s); *Note: ^{1}H–NMR featured broad signals due to rotamers.* δ_H (400 MHz, $CDCl_3$) 1.57 (9H, s), 2.89 (3H, s), 3.05–3.13 (2H, m), 3.50 (2H, t, $^3J_{HH}$ 8.0), 6.46 (1H, br), 6.84 (1H, s), 7.13 (1H, t, $^3J_{HH}$ 7.0), 7.35 (1H, t, $^3J_{HH}$ 8.5), 7.42–7.46 (2H, m), 7.94 (1H, d, $^3J_{HH}$ 7.5), 10.0 (1H, s); *Note: ^{13}C–NMR featured broad signals due to rotamers.* δ_C (100 MHz, $CDCl_3$) 28.6 ($(CH_3)_3$), 31.2 (CH_2), 35.6 (CH_3), 49.7 (CH_2), 80.5 (C), 104.0 (CH), 111.4 (CH), 115.1 (CH), 118.5 (CH), 120.3 (CH), 122.1 & 122.4 (C), 123.2 & 123.8 (C), 125.6 (CH), 129.3 & 129.6 (C), 134.3 (C), 140.7 (C), 149.2 (C), 156.8 (C=O); HRMS (ESI$^+$): Found: 363.1684; $C_{20}H_{24}N_2NaO_3$ (MNa^+) Requires 363.1679 (–1.2 ppm error).

1-Phenyl-1,2-dihydro-3*H*-cyclopenta[c]quinolin-3-one (7a)

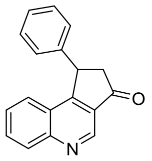

Synthesised using general procedure D with 1-(1*H*-indol-3-yl)-4-phenylbut-3-yn-2-one **1a** (108 mg, 0.410 mmol), AgOTf (2.1 mg, 0.008 mmol), isopropanol (4.1 mL), at RT for 2 h, then $AlCl_3{\cdot}6H_2O$ (5 mg, 0.02 mmol), and heated using a microwave at 100 °C for 1 h. Purification by silica column chromatography (20–50% EtOAc in hexane) afforded the title compound as an orange solid (95 mg, 88%); Mp 90–95 °C; R_f 0.60 (50% EtOAc in hexane); ν_{max} (thin film)/cm^{-1} 3062 (w), 3027 (w), 2919 (w), 1710 (s), 1574 (m), 1235 (m), 762 (m), 700 (s); δ_H (400 MHz, $CDCl_3$) 2.76 (1H, dd, $^2J_{HH}$ 19.0, $^3J_{HH}$ 3.0), 3.40 (1H, dd, $^2J_{HH}$ 19.0, $^3J_{HH}$ 7.5), 5.02 (1H, dd, $^3J_{HH}$ 7.5, $^3J_{HH}$ 3.0), 7.08–7.11 (1H, m), 7.23–7.32 (3H, m), 7.46 (1H, ddd, $^3J_{HH}$ 8.5, $^3J_{HH}$ 7.0, $^4J_{HH}$ 1.5), 7.69 (1H, d, $^3J_{HH}$ 7.5), 7.80 (1H, ddd, $^3J_{HH}$ 8.5, $^3J_{HH}$ 7.0, $^4J_{HH}$ 1.5), 8.21 (1H, d, $^3J_{HH}$ 8.5), 9.29 (1H, s); δ_C (100 MHz, $CDCl_3$) 42.7 (CH), 46.5 (CH_2), 124.4 (CH), 126.1 (2 x CH), 126.2 (CH), 126.5 (C), 128.1 (2 x CH), 129.0 (C), 129.4 (CH), 131.1 (CH), 141.4 (C), 144.4 (CH), 149.7 (C), 164.2 (C), 203.0 (C=O); HRMS (ESI$^+$): Found: 282.0877; $C_{18}H_{13}NNaO$ (MNa^+) Requires 282.0889 (4.3 ppm error), Found: 260.1061; $C_{18}H_{14}NO$ (MH^+) Requires 260.1070 (3.3 ppm error).

Batch heating: To a 100 mL round-bottom flask was added a 0.1 M isopropanol (40 mL) solution of 1-(1*H*-indol-3-yl)-4-phenylbut-3-yn-2-one **1a** (1.0 g, 3.8 mmol), AgOTf (10 mg, 0.038 mmol), and the reaction was stirred for 4 h under air, during which time the orange solution became cloudy. $AlCl_3.6H_2O$ (46 mg, 0.19 mmol) was added and the reaction heated at reflux under an air atmosphere for 8 h. The solution was concentrated and purified by column chromatography (silica), eluting with 20–50% EtOAc in hexane, yielding the title compound as an orange solid (827 mg, 82%).

LHMDS mediated rearrangement: To a 25 mL oven-dried round-bottom flask was added 2-phenylspiro[cyclopentane-1,3'-indol]-2-en-4-one **3a** (100 mg, 0.38 mmol), dry THF (2 mL), and the solution cooled to –78 °C under argon. LHMDS (0.40 mL, 1.0 M in THF, 0.40 mmol) was added dropwise, which caused the orange solution to turn brown, and the reaction was then stirred for 0.5 h, and warmed to RT. The reaction as quenched with saturated aqueous NH_4Cl (10 mL), extracted successively with EtOAc (3 x 10 mL), dried ($MgSO_4$) and the green organic solution concentrated to a crude oil. The product was purified by column chromatography (silica) eluting with 20–60% EtOAc in hexane, yielding the product as an orange solid (81 mg, 81%).

1-(4-Bromophenyl)-1,2-dihydro-3*H*-cyclopenta[c]quinolin-3-one (7b)

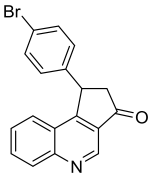

Synthesised using general procedure D with 4-(4-bromophenyl)-1-(1*H*-indol-3-yl)but-3-yn-2-one **1b** (100 mg, 0.296 mmol), AgOTf (1.9 mg, 0.007 mmol), isopropanol (2.9 mL), at RT for 2 h, then $AlCl_3{\cdot}6H_2O$ (3.5 mg, 0.014 mmol), and heated using a microwave at 100 °C for 1 h. Purification by silica column chromatography (20–50% EtOAc in hexane) afforded the title compound as an orange solid (87 mg, 87%); Mp 45–49 °C; R_f 0.39 (50% EtOAc in hexane); ν_{max} (thin film)/cm^{-1} 2924 (w), 1714 (s), 1570 (m), 1487 (m), 1275 (m), 1010 (m), 770 (m); δ_H (400 MHz, $CDCl_3$) 2.70 (1H, dd, $^2J_{HH}$ 19.0, $^3J_{HH}$ 3.0), 3.39 (1H, dd, $^2J_{HH}$ 19.0, $^3J_{HH}$ 7.5), 4.99 (1H, dd, $^3J_{HH}$ 7.5, $^3J_{HH}$ 3.0), 6.97 (2H, d, $^3J_{HH}$ 8.5), 7.42 (2H, d, $^3J_{HH}$ 8.5), 7.49 (1H, t, $^3J_{HH}$ 7.5), 7.64 (1H, d, $^3J_{HH}$ 8.5), 7.82 (1H, t, $^3J_{HH}$ 8.5), 8.22 (1H, d, $^3J_{HH}$ 8.5), 9.28 (1H, s); δ_C (100 MHz, $CDCl_3$) 43.3 (CH), 47.5 (CH_2), 121.4 (C), 125.2 (C), 125.5 (CH), 127.98 (CH), 129.1 (CH x 2), 130.3 (CH), 130.8 (C), 132.5 (CH), 132.6 (CH x 2), 141.65 (C), 145.7 (CH), 151.0 (C), 164.7 (C), 203.7 (C=O); HRMS (ESI$^+$): Found: Found: 338.0168; $C_{18}H_{13}{}^{79}BrNO$ (MH^+) Requires 338.0175 (2.2 ppm error).

1-(4-Methoxyphenyl)-1,2-dihydro-3*H*-cyclopenta[c]quinolin-3-one (7c)

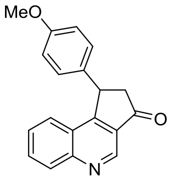

Synthesised using general procedure D with 1-(1*H*-indol-3-yl)-4-(4-methoxyphenyl)but-3-yn-2-one **1c** (80 mg, 0.27 mmol), AgOTf (1.5 mg, 0.005 mmol), isopropanol (2.7 mL), at RT for 2 h, then $AlCl_3{\cdot}6H_2O$ (3.3 mg, 0.014 mmol), and heated using a microwave at 100 °C for 1 h. Purification by silica column chromatography (20–50% EtOAc in hexane) afforded the title compound as a pale orange solid (69 mg, 86%); Mp 126–128 °C; R_f 0.50 (50% EtOAc in hexane); ν_{max} (thin film)/cm^{-1} 3004 (w), 2932 (w), 2835 (w), 1709 (s), 1572 (m), 1509 (s), 1245 (s), 767 (m); δ_H (400 MHz, $CDCl_3$) 2.70 (1H, dd, $^2J_{HH}$ 19.0, $^3J_{HH}$ 3.0), 3.34 (1H, dd, $^2J_{HH}$ 19.0, $^3J_{HH}$ 7.5), 3.74 (3H, s), 4.96 (1H, dd, $^3J_{HH}$ 7.5, $^3J_{HH}$ 3.0), 6.80 (2H, d, $^3J_{HH}$ 8.5), 6.69 (2H, d, $^3J_{HH}$ 8.5), 7.44 (1H, t, $^3J_{HH}$ 7.5), 7.70 (1H, d, $^3J_{HH}$ 8.5), 7.77 (1H, t, $^3J_{HH}$ 7.5), 8.18 (1H, d, $^3J_{HH}$ 8.5), 9.25 (1H, s); δ_C (100 MHz, $CDCl_3$) 43.2 (CH), 47.9 (CH_2), 55.3 (CH_3), 114.7 (2 x CH), 125.4 (C), 125.8 (CH), 127.7 (CH), 128.5 (2 x CH), 130.2 (C), 130.6 (CH), 132.3 (CH), 134.7 (C), 145.6 (CH), 151.0 (C), 158.8 (C), 165.7 (C), 204.5 (C=O); HRMS (ESI$^+$): Found: 312.0987; $C_{19}H_{15}NNaO_2$ (MNa^+) Requires 312.0995 (2.6 ppm error), Found: 290.1171; $C_{19}H_{16}NO_2$ (MH^+) Requires 290.1176 (1.4 ppm error).

1-(4-(Dimethylamino)phenyl)-1,2-dihydro-3*H*-cyclopenta[c]quinolin-3-one (7d)

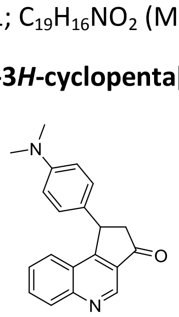

Synthesised using general procedure D with 4-(4-(dimethylamino)phenyl)-1-(1*H*-indol-3-yl)but-3-yn-2-one **1d** (85 mg, 0.28 mmol), AgOTf (1.5 mg, 0.005 mmol), isopropanol (2.8 mL), at RT for 1 h, then $AlCl_3{\cdot}6H_2O$ (6.8 mg, 0.028 mmol), and heated using a microwave at 100 °C for 1.5 h. Purification by

silica column chromatography (20–50% EtOAc in hexane) afforded the title compound as a pale brown solid (57 mg, 67%); Mp 146–150 °C; R_f 0.47 (50% EtOAc in hexane); ν_{max} (thin film)/cm^{-1} 2891 (w), 2807 (w), 1701 (s), 1612 (s), 1519 (s), 1513 (s), 1351 (m), 814 (s), 756 (s); δ_H (400 MHz, $CDCl_3$) 2.72 (1H, dd, $^2J_{HH}$ 19.0, $^3J_{HH}$ 3.0), 2.90 (6H, s), 3.34 (1H, dd, $^2J_{HH}$ 19.0, $^3J_{HH}$ 7.5), 4.93 (1H, dd, $^3J_{HH}$ 7.5, $^3J_{HH}$ 3.0), 6.62 (2H, d, $^3J_{HH}$ 9.0), 6.94 (2H, d, $^3J_{HH}$ 9.0), 7.46 (1H, app. t, $^3J_{HH}$ 7.0), 7.74–7.79 (2H, m), 8.18 (1H, d, $^3J_{HH}$ 7.5), 9.26 (1H, s); δ_C (100 MHz, $CDCl_3$) 40.5 ($(CH_3)_2$), 43.2 (CH), 48.0 (CH_2) 113.1 (2 x CH), 125.6 (C), 125.9 (CH), 127.7 (CH), 128.1 (2 x CH), 130.1 (CH), 130.15 (C), 130.5 (C), 132.2 (CH), 145.6 (CH), 149.7 (C), 150.9 (C), 166.2 (C), 205.0 (C=O); HRMS (ESI$^+$): Found: 325.1310; $C_{20}H_{18}N_2NaO$ (MNa^+) Requires 325.1311 (0.4 ppm error), Found: 303.1493; $C_{20}H_{19}N_2O$ (MH^+) Requires 303.1492 (–0.2 ppm error).

2-Benzyl-1-(4-methoxyphenyl)-1,2-dihydro-3*H*-cyclopenta[c]quinolin-3-one (7e)

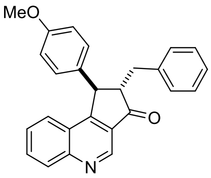

Synthesised using general procedure C with 4-(1*H*-indol-3-yl)-1-(4-methoxyphenyl)-5-phenylpent-1-yn-3-one **1e** (100 mg, 0.26 mmol), AgOTf (1 mg, 0.004 mmol), isopropanol (2.6 mL), at RT for 3 h, then $AlCl_3·6H_2O$ (6.3 mg, 0.026 mmol), and heated using a microwave at 100 °C for 1.5 h. Purification by silica column chromatography (20–50% EtOAc in hexane) afforded the title compound as an orange solid (79 mg, 79%); Mp 40–45 °C; R_f 0.57 (50% EtOAc in hexane); ν_{max} (thin film)/cm^{-1} 3062 (w), 3027 (w), 2929 (w), 2836 (w), 1710 (s), 1609 (m), 1572 (m), 1509 (s), 1248 (s), 1031 (m), 731 (m); δ_H (400 MHz, $CDCl_3$) 2.91 (1H, dd, $^2J_{HH}$ 14.0, $^3J_{HH}$ 9.0), 3.04 (1H, ddd, $^3J_{HH}$ 9.0, $^3J_{HH}$ 5.0, $^3J_{HH}$ 3.0), 3.38 (1H, dd, $^2J_{HH}$ 14.0, $^3J_{HH}$ 5.0), 3.72 (3H, s), 4.59 (1H, d, $^3J_{HH}$ 3.0), 6.57 (2H, d, $^3J_{HH}$ 8.5), 6.67 (2H, d, $^3J_{HH}$ 8.5), 7.22–7.31 (5H, m), 7.39 (1H, ddd, $^3J_{HH}$ 8.5, $^3J_{HH}$ 7.0, $^4J_{HH}$ 1.5), 7.50 (1H, dd, $^3J_{HH}$ 8.5, $^4J_{HH}$ 1.5), 7.76 (1H, app. t, $^3J_{HH}$ 7.5), 8.17 (1H, d, $^3J_{HH}$ 8.5), 9.28 (1H, s); δ_C (100 MHz, $CDCl_3$) 36.7 (CH_2), 48.5 (CH), 55.3 (CH_3), 60.4 (CH), 114.4 (2 x CH), 125.4 (C), 125.9 (CH), 126.9 (C), 127.6 (CH), 128.5 (CH), 128.8 (2 x CH), 129.5 (2 x CH), 130.6 (2 x CH), 132.3 (CH), 134.3 (C), 138.5 (C), 145.8 (CH), 151.1 (C), 158.6 (C), 164.7 (C), 205.8 (C=O); HRMS (ESI$^+$): Found: 402.1454; $C_{26}H_{21}NNaO_2$ (MNa^+) Requires 402.1465 (2.6 ppm error), Found: 380.1631; $C_{26}H_{22}NO_2$ (MH^+) Requires 380.1645 (3.6 ppm error).

8-Bromo-1-(4-methoxyphenyl)-1,2-dihydro-3*H*-cyclopenta[c]quinolin-3-one (7f)

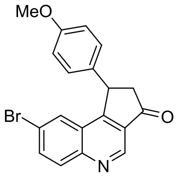

Synthesised using general procedure E with 1-(5-bromo-1*H*-indol-3-yl)-4-(4-methoxyphenyl)but-3-yn-2-one **1f** (104 mg, 0.283 mmol), AgOTf (1.5 mg, 0.006 mmol), CH_2Cl_2 (2.8 mL), at RT for 2 h, then $AlCl_3·6H_2O$ (3.5 mg, 0.014 mmol), isopropanol (2.8 mL), and heated using a microwave at 100 °C for 1 h. Purification by silica column chromatography (20–50% EtOAc in hexane) afforded the title compound as an orange solid (78 mg, 75%); Mp 167–180 °C; R_f 0.56 (50% EtOAc in hexane); ν_{max} (thin film)/cm^{-1} 3053 (w), 1703 (s), 1567 (m), 1510 (s), 1251 (s), 854 (s), 555 (m); δ_H (400 MHz, $CDCl_3$) 2.72 (1H, dd, $^2J_{HH}$ 19.0, $^3J_{HH}$ 3.0), 3.36 (1H, dd, $^2J_{HH}$ 19.0, $^3J_{HH}$ 7.5), 3.78 (3H, s), 4.92 (1H, dd, $^3J_{HH}$ 7.5, $^3J_{HH}$ 3.0), 6.83 (2H, d, $^3J_{HH}$ 8.5), 6.99 (2H, d, $^3J_{HH}$ 8.5), 7.83–7.86 (1H, m), 8.07 (1H, d, $^3J_{HH}$ 10.0), 9.26 (1H, s); δ_C (100 MHz, $CDCl_3$) 43.1 (CH), 47.9 (CH_2), 55.4 (CH_3), 114.9 (2 x CH), 121.9 (C), 126.8 (C), 127.9 (CH),

128.4 (2 x CH), 130.6 (C), 132.3 (CH), 133.9 (C), 135.6 (CH), 145.9 (CH), 149.6 (C), 159.0 (C), 164.6 (C), 204.1 (C=O); HRMS (ESI$^+$): Found: 390.0081; $C_{19}H_{14}{}^{79}BrNNaO_2$ (MNa$^+$) Requires 390.0100 (4.9 ppm error), Found: 368.0269; $C_{19}H_{15}{}^{79}BrNO_2$ (MH$^+$) Requires 368.0281 (3.1 ppm error).

1-Butyl-1,2-dihydro-3*H*-cyclopenta[c]quinolin-3-one (7g)

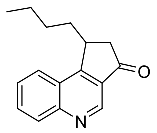

Synthesised using general procedure D with 1-(1*H*-indol-3-yl)oct-3-yn-2-one **1g** (98 mg, 0.41 mmol), AgOTf (1 mg, 0.004 mmol), isopropanol (4 mL), at RT for 2 h, then $AlCl_3{\cdot}6H_2O$ (5 mg, 0.02 mmol), and heated using a microwave at 100 °C for 1 h. Purification by silica column chromatography (20–50% EtOAc in hexane) afforded the title compound as an orange solid (80 mg, 81%); R_f 0.60 (50% EtOAc in hexane); ν_{max} (thin film)/cm^{-1} 2956 (m), 2929 (m), 2856 (w), 1712 (s), 1573 (m), 1234 (m), 769 (m); δ_H (400 MHz, $CDCl_3$) 0.88 (3H, t, $^3J_{HH}$ 7.0), 1.20–1.45 (5H, m), 1.50–1.60 (1H, m), 2.10–2.19 (1H, m), 2.60 (1H, dd, $^2J_{HH}$ 19.0, $^3J_{HH}$ 2.5), 2.97 (1H, dd, $^2J_{HH}$ 19.0, $^3J_{HH}$ 7.0), 3.87–3.93 (1H, m), 7.68 (1H, t, $^3J_{HH}$ 7.5), 7.87 (1H, app. t, $^3J_{HH}$ 7.5), 8.10 (1H, d, $^3J_{HH}$ 8.5), 8.23 (1H, d, $^3J_{HH}$ 8.5) 9.16 (1H, s); δ_C (100 MHz, $CDCl_3$) 12.7 (CH_3), 21.5 (CH_2), 28.4 (CH_2), 34.7 (CH_2), 36.4 (CH), 42.2 (CH_2), 123.4 (CH), 124.1 (C), 126.4 (CH), 128.3 (C), 129.6 (CH), 131.1 (CH), 145.5 (CH), 149.3 (C), 166.6 (C), 203.6 (C=O); HRMS (ESI$^+$): Found: 262.1204; $C_{16}H_{17}NNaO$ (MNa$^+$) Requires 262.1202 (–0.8 ppm error), Found: 240.1385; $C_{16}H_{18}NO$ (MH$^+$) Requires 240.1383 (–0.9 ppm error).

1-(2-((tert-Butyldimethylsilyl)oxy)ethyl)-1,2-dihydro-3*H*-cyclopenta[c]quinolin-3-one (7h)

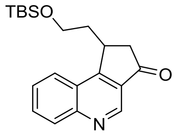

Synthesised using general procedure E with 6-((tert-butyldimethylsilyl)oxy)-1-(1*H*-indol-3-yl)hex-3-yn-2-one **1h** (86 mg, 0.25 mmol), AgOTf (1.3 mg, 0.005 mmol), CH_2Cl_2 (2.5 mL), at RT for 1 h, then $AlCl_3{\cdot}6H_2O$ (3 mg, 0.012 mmol), isopropanol (2.5 mL), and heated using a microwave at 100 °C for 1 h. Purification by silica column chromatography (20–50% EtOAc in hexane) afforded the title compound as an orange oil (65 mg, 75%); R_f 0.63 (50% EtOAc in hexane); ν_{max} (thin film)/cm^{-1} 2952 (m), 2928 (m), 2856 (m), 1713 (s), 1254 (m), 1101 (m), 831 (s), 770 (s); δ_H (400 MHz, $CDCl_3$) 0.06 (3H, s), 0.07 (3H, s), 0.91 (9H, s), 1.62 (1H, dddd, $^2J_{HH}$ 14.5, $^3J_{HH}$ 10.0, $^3J_{HH}$ 4.0, $^3J_{HH}$ 4.0), 2.41 (1H, dddd, $^2J_{HH}$ 14.5, $^3J_{HH}$ 8.5, $^3J_{HH}$ 5.0, $^3J_{HH}$ 2.5), 2.67 (1H, dd, $^2J_{HH}$ 19.0, $^3J_{HH}$ 1.5), 2.98 (1H, dd, $^2J_{HH}$ 19.0, $^3J_{HH}$ 7.5), 3.70 (1H, td, $^2J_{HH}$ 10.0, $^3J_{HH}$ 3.5), 3.82 (1H, dt, $^2J_{HH}$ 10.0, $^3J_{HH}$ 5.5), 4.09–4.14 (1H, m), 7.67 (1H, app. t, $^3J_{HH}$ 7.0), 7.87 (1H, ddd, $^3J_{HH}$ 10.0, $^3J_{HH}$ 7.0, $^4J_{HH}$ 1.5), 8.16 (1H, dd, $^3J_{HH}$ 8.5, $^4J_{HH}$ 1.5), 8.22 (1H, d, $^3J_{HH}$ 8.5), 9.16 (1H s); δ_C (100 MHz, $CDCl_3$) –5.3 ($SiMe_2$), 18.3 (SiC), 26.0 (Si*t*Bu), 34.6 (CH), 39.5 (CH_2), 43.7 (CH_2), 61.2 (CH_2), 124.9 (CH), 125.3 (C), 127.6 (CH), 129.6 (C), 130.8 (CH), 132.3 (CH), 145.8 (CH), 150.6 (C), 168.2 (C), 204.8 (C=O); HRMS (ESI$^+$): Found: 364.1692; $C_{20}H_{27}NNaO_2Si$ (MNa$^+$) Requires 364.1703 (3.0 ppm error), Found: 342.1875; $C_{20}H_{28}NO_2Si$ (MH$^+$) Requires 342.1884 (2.6 ppm error).

1-(3-((tert-Butyldimethylsilyl)oxy)propyl)-1,2-dihydro-3*H*-cyclopenta[c]quinolin-3-one (7i)

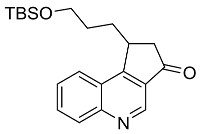

Synthesised using general procedure E with 7-((tert-butyldimethylsilyl)oxy)-1-(1*H*-indol-3-yl)hept-3-yn-2-one **1i** (64 mg, 0.18 mmol), AgOTf (1 mg, 0.004 mmol), CH_2Cl_2 (1.8 mL), at RT for 1 h, then $AlCl_3{\cdot}6H_2O$ (3 mg, 0.012 mmol), isopropanol (1.8 mL), and heated using a microwave at 100 °C for 1 h. Purification by silica column chromatography (20–50% EtOAc in hexane) afforded the title compound as a yellow oil (46 mg, 71%); R_f 0.75 (50% EtOAc in hexane); ν_{max} (thin film)/cm^{-1} 2952 (m), 2928 (m), 2856 (m), 1714 (s), 1254 (m), 1100 (m), 834 (s), 773 (s); δ_H (400 MHz, $CDCl_3$) –0.01 (3H, s), 0.86 (3H, s), 1.47–1.67 (3H, m), 2.30–2.36 (1H, m), 2.58 (1H, dd, $^2J_{HH}$ 19.0, $^3J_{HH}$ 1.5), 2.98 (1H, dd, $^2J_{HH}$ 19.0, $^3J_{HH}$ 7.5), 3.59–3.70 (2H, m), 3.92–3.98 (1H, m), 7.66 (1H, app. t, $^3J_{HH}$ 7.0), 7.86 (1H, app. t, $^3J_{HH}$ 7.0), 8.14 (1H, d, $^3J_{HH}$ 8.5), 8.22 (1H, d, $^3J_{HH}$ 8.5) 9.16 (1H, s); δ_C (100 MHz, $CDCl_3$) –5.3 ($SiMe_2$), 18.3 (SiC), 26.0 (Si*t*Bu), 30.4 (CH_2), 32.4 (CH_2), 37.2 (CH), 43.3 (CH_2), 62.2 (CH_2), 124.9 (CH), 125.4 (C), 127.6 (CH), 129.5 (C), 130.8 (CH), 132.3 (CH), 145.8 (CH), 150.6 (C), 167.8 (C), 204.7 (C=O); HRMS (ESI$^+$): Found: 378.1851; $C_{21}H_{29}NNaO_2Si$ (MNa^+) Requires 378.1860 (2.3 ppm error), Found: 356.2030; $C_{21}H_{30}NO_2Si$ (MH^+) Requires 356.2040 (2.8 ppm error).

tert-Butyl methyl(2-(3-oxo-2,3-dihydro-1*H*-cyclopenta[c]quinolin-1-yl)ethyl)carbamate (7j)

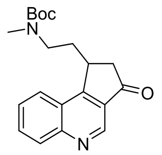

Synthesised using general procedure E with tert-butyl (6-(1*H*-indol-3-yl)-5-oxohex-3-yn-1-yl)(methyl)carbamate **1j** (88 mg, 0.26 mmol), AgOTf (1 mg, 0.004 mmol), CH_2Cl_2 (2.5 mL), at RT for 1 h, then $AlCl_3{\cdot}6H_2O$ (6.2 mg, 0.025 mmol), isopropanol (2.5 mL), and heated using a microwave at 100 °C for 2 h. Purification by silica column chromatography (20–80% EtOAc in hexane) afforded the title compound as an orange oil (58 mg, 65%); R_f 0.34 (50% EtOAc in hexane); ν_{max} (thin film)/cm^{-1} 2974 (w), 2929 (w), 1712 (s), 1687 (s), 1588 (m), 1392 (m), 1156 (s), 767 (m); *Note: ^{1}H–NMR featured broad signals due to rotamers.* δ_H (400 MHz, $CDCl_3$) 1.43 (9H, s), 1.51–1.62 (1H, m), 2.38–2.50 (1H, m), 2.63 (1H, d, $^2J_{HH}$ 17.0), 2.89 (3H, br), 2.99 (1H, dd, $^2J_{HH}$ 19.0, $^3J_{HH}$ 7.5), 3.10–3.60 (2H, br), 3.87 (1H, t, $^3J_{HH}$ 9.0), 7.67 (1H, t, $^3J_{HH}$ 8.5), 7.87 (1H, t, $^3J_{HH}$ 7.5), 8.05 (1H, br), 8.21 (1H, d, $^3J_{HH}$ 8.5), 9.14 (1H, s); *Note: ^{13}C–NMR featured broad signals due to rotamers.* δ_C (100 MHz, $CDCl_3$) 28.5 (*t*Bu), 34.6 (CH_3), 35.0 (CH_2), 35.6 (CH), 43.4 (CH_2), 47.5 (CH_2), 80.0 (C). 124.5 (CH), 125.1 (C), 127.9 (CH), 129.4 (C), 131.0 (CH), 132.4 (CH), 145.8 (CH), 150.6 (C), 155.6 (C=O), 167.3 (C), 204.1 (C=O); HRMS (ESI$^+$): Found: 363.1677; $C_{20}H_{24}N_2NaO_3$ (MNa^+) Requires 363.1679 (0.7 ppm error), Found: 341.1855; $C_{20}H_{25}N_2O_3$ (MH^+) Requires 341.1860 (1.4 ppm error).

1-(4-Methoxyphenyl)-4-methyl-1,2-dihydro-3*H*-cyclopenta[c]quinolin-3-one (7k)

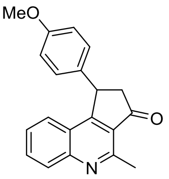

Synthesised using general procedure D with 4-(4-methoxyphenyl)-1-(2-methyl-1*H*-indol-3-yl)but-3-yn-2-one **1k** (91 mg, 0.30 mmol), AgOTf (1.5 mg, 0.005 mmol), isopropanol (3 mL), at RT for 1 h, then $AlCl_3{\cdot}6H_2O$ (7.2 mg, 0.03 mmol), and heated using a microwave at 100 °C for 1.5 h. Purification by silica column chromatography (20–50% EtOAc in hexane) afforded the title compound as a yellow solid (80 mg, 87%); Mp 127–132 °C; R_f 0.58 (50% EtOAc in hexane); ν_{max} (thin film)/cm^{-1} 2998 (w), 2955 (w), 2926 (w), 1705 (s), 1503 (m), 1249 (s), 1029 (s), 772 (s); δ_H (400 MHz, $CDCl_3$) 2.67 (1H, dd,

$^{2}J_{HH}$ 19.0, $^{3}J_{HH}$ 3.0), 3.01 (3H, s), 3.31 (1H, dd, $^{2}J_{HH}$ 19.0, $^{3}J_{HH}$ 8.5), 3.74 (3H, s), 4.90 (1H, dd, $^{3}J_{HH}$ 8.5, $^{3}J_{HH}$ 3.0), 6.79 (2H, d, $^{3}J_{HH}$ 8.5), 6.68 (2H, d, $^{3}J_{HH}$ 8.5), 7.36 (1H, app. t, $^{3}J_{HH}$ 7.5), 7.65 (1H, d, $^{3}J_{HH}$ 7.5), 7.73 (1H, app. t, $^{3}J_{HH}$ 7.5), 8.08 (1H, d, $^{3}J_{HH}$ 8.5); δ_C (100 MHz, $CDCl_3$) 22.8 (CH_3), 42.4 (CH), 48.1 (CH_2), 55.3 (CH_3), 114.7 (2 x CH), 124.5 (C), 125.5 (CH), 126.6 (CH), 128.4 (2 x CH), 128.8 (C), 129.5 (CH), 132.4 (CH), 135.1 (C), 150.1 (C), 157.1 (C), 158.7 (C), 166.7 (C), 205.1 (C=O); HRMS (ESI^+): Found: 326.1329; $C_{20}H_{17}NNaO_2$ (MNa^+) Requires 326.1151 (3.9 ppm error), Found: 304.1329; $C_{20}H_{18}NO_2$ (MH^+) Requires 304.1332 (0.9 ppm error).

1-(4-Methoxyphenyl)-4-phenyl-1,2-dihydro-3*H*-cyclopenta[c]quinolin-3-one (7l)

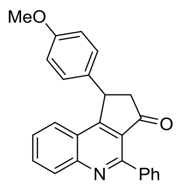

Synthesised using general procedure D with 4-(4-methoxyphenyl)-1-(2-phenyl-1*H*-indol-3-yl)but-3-yn-2-one **1l** (50 mg, 0.14 mmol), AgOTf (1 mg, 0.004 mmol), isopropanol (1.4 mL), at RT for 1.5 h, then $AlCl_3{\cdot}6H_2O$ (3.3 mg, 0.014 mmol), and heated using a microwave at 100 °C for 1 h. Purification by silica column chromatography (20–50% EtOAc in hexane) afforded the title compound as a yellow solid (46 mg, 92%); Mp 160–164 °C; R_f 0.70 (50% EtOAc in hexane); ν_{max} (thin film)/cm^{-1} 3064 (w), 2989 (w), 2905 (w), 2835 (w), 1713 (s), 1610 (m), 1508 (s), 1246 (s), 834 (s), 771 (m), 702 (s); δ_H (400 MHz, $CDCl_3$) 2.73 (1H, dd, $^{2}J_{HH}$ 19.0, $^{3}J_{HH}$ 3.0), 3.35 (1H, dd, $^{2}J_{HH}$ 19.0, $^{3}J_{HH}$ 8.5), 3.76 (3H, s), 4.97 (1H, dd, $^{3}J_{HH}$ 8.5, $^{3}J_{HH}$ 3.0), 6.83 (2H, d, $^{3}J_{HH}$ 8.5), 7.05 (2H, d, $^{3}J_{HH}$ 8.5), 7.43 (1H, t, $^{3}J_{HH}$ 7.5), 7.48–7.54 (3H, m), 7.75–7.81 (2H, m), 7.86–7.88 (2H, m), 8.22 (1H, d, $^{3}J_{HH}$ 8.5); δ_C (100 MHz, $CDCl_3$) 43.3 (CH), 48.2 (CH_2), 55.4 (CH_3), 114.7 (2 x CH), 124.7 (C), 125.5 (CH), 127.3 (CH), 127.8 (C), 128.0 (2 x CH), 128.5 (2 x CH), 129.6 (CH), 129.9 (2 x CH), 130.5 (C), 132.6 (CH), 135.2 (C), 137.8 (C), 150.2 (C), 157.0 (C), 158.8 (C), 167.8 (C), 203.0 (C=O); HRMS (ESI^+): Found: 388.1300; $C_{25}H_{19}NNaO_2$ (MNa^+) Requires 388.1308 (2.1 ppm error), Found: 366.1484; $C_{25}H_{20}NO_2$ (MH^+) Requires 366.1489 (1.3 ppm error).

1-Phenyl-4-(phenylthio)-1,2-dihydro-3*H*-cyclopenta[c]quinolin-3-one (7m)

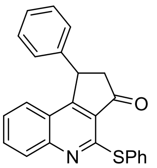

Synthesised using general procedure E with 4-phenyl-1-(2-(phenylthio)-1*H*-indol-3-yl)but-3-yn-2-one **1m** (57 mg, 0.15 mmol), AgOTf (1 mg, 0.004 mmol), CH_2Cl_2 (1.5 mL), at RT for 1.5 h, then $AlCl_3{\cdot}6H_2O$ (3.3 mg, 0.014 mmol), isopropanol (1.5 mL) and heated using a microwave at 100 °C for 1 h. Purification by silica column chromatography (20–50% EtOAc in hexane) afforded the title compound as an off-white solid (44 mg, 77%); Mp 137–142 °C; R_f 0.85 (50% EtOAc in hexane); ν_{max} (thin film)/cm^{-1} 3060 (w), 1703 (s), 1611 (m), 1576 (s), 1546 (s), 1233 (s), 952 (m), 747 (m), 688 (s); δ_H (400 MHz, $CDCl_3$) 2.76 (1H, dd, $^{2}J_{HH}$ 19.0, $^{3}J_{HH}$ 3.0), 3.40 (1H, dd, $^{2}J_{HH}$ 19.0, $^{3}J_{HH}$ 8.5), 4.96 (1H, dd, $^{3}J_{HH}$ 8.5, $^{3}J_{HH}$ 3.0), 7.11 (2H, d, $^{3}J_{HH}$ 7.0), 7.22–7.31 (4H, m), 7.45–7.50 (3H, m), 7.55 (1H, d, $^{3}J_{HH}$ 8.5), 7.60 (1H, d, $^{3}J_{HH}$ 7.0), 7.67–7.71 (3H, m); δ_C (100 MHz, $CDCl_3$) 43.8 (CH), 47.5 (CH_2), 123.5 (C), 125.5 (CH), 126.1 (CH), 127.4 (2 x CH), 127.5 (CH), 128.0 (C), 129.8 (C), 129.1 (2 x CH), 129.4 (2 x CH), 129.4 (CH), 132.5 (CH), 135.9 (2 x CH), 142.7 (C), 150.6 (C), 156.9 (C), 166.8 (C), 203.5 (C=O); HRMS (ESI^+): Found: 390.0923; $C_{24}H_{17}NNaOS$ (MNa^+) Requires 390.0923 (–0.1 ppm error), Found: 368.1108; $C_{24}H_{18}NOS$ (MH^+) Requires 368.1104 (1.4 ppm error).

4,5,6a,7-Tetrahydrocyclopenta[3,4]pyrano[2,3-b]indol-2(1*H*)-one (8h)

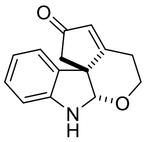

To a 10 mL sample vial containing a 0.1 M CH_2Cl_2 (4.4 mL) solution of 6-((tert-butyldimethylsilyl)oxy)-1-(*1H*-indol-3-yl)hex-3-yn-2-one **1h** (150 mg, 0.44 mmol) was added AgOTf (1 mg, 0.004 mmol) and the reaction stirred under air at RT. After 1 h, the reaction was concentrated under vacuum and the solvent replaced with THF (3 mL). 10% Aqueous HCl (0.25 mL) was added, and the reaction stirred at RT under air for 0.5 h. The reaction was diluted with EtOAc (20 mL), washed sequentially with saturated aqueous $NaHCO_3$ (10 mL) and brine (10 mL), and the combined aqueous washes back-extracted with EtOAc (5 mL). The combined organic layers were dried ($MgSO_4$), concentrated and purified by column chromatography (silica) eluting with 20% to 50% EtOAc in hexane, affording the title compound as an off-white solid (82 mg, 82%); Mp 139–143 °C R_f 0.26 (50% EtOAc in hexane); ν_{max} (thin film)/cm^{-1} 3320 (s, NH), 1702 (s, C=O), 1618 (s), 1463 (s), 1031 (s), 698 (s); δ_H (400 MHz, $CDCl_3$) 2.29 (1H, d, $^2J_{HH}$ 18.5), 2.52 (1H, dddd, $^2J_{HH}$ 14.5, $^3J_{HH}$ 10.5, $^3J_{HH}$ 6.0, $^4J_{HH}$ 1.5), 2.27 (1H, dt, $^2J_{HH}$ 14.5, $^3J_{HH}$ 3.0), 2.74 (1H, d, $^2J_{HH}$ 18.5), 3.50 (1H, ddd, $^2J_{HH}$ 11.5, $^3J_{HH}$ 10.0, $^3J_{HH}$ 3.0), 3.96 (1H, ddd, $^2J_{HH}$ 11.5, $^3J_{HH}$ 5.5, $^3J_{HH}$ 3.0), 4.63 (1H, br, NH), 4.93 (1H, s), 6.20 (1H, d, $^4J_{HH}$ 1.5), 6.74 (1H, d, $^3J_{HH}$ 7.5), 6.79 (1H, t, $^3J_{HH}$ 7.5), 6.87 (1H, d, $^3J_{HH}$ 7.5), 7.13 (1H, d, $^3J_{HH}$ 7.5); δ_C (100 MHz, $CDCl_3$) 29.1 (CH_2), 48.4 (CH_2), 56.5 (C), 62.0 (CH_2), 96.3 (CH), 110.0 (CH), 120.3 (CH), 122.0 (CH), 128.9 (CH), 130.4 (CH), 131.0 (C), 148.9 (C), 176.1 (C), 206.3 (C=O); HRMS (ESI$^+$): Found: 250.0839; $C_{14}H_{13}NNaO_2$ (MNa^+) Requires 250.0838 (–0.2 ppm error), Found: 228.1018; $C_{14}H_{14}NO_2$ (MH^+) Requires 228.1019 (0.7 ppm error).

(6aS,11bS)-4,5,6a,7-Tetrahydrocyclopenta[3,4]pyrano[2,3-b]indol-2(1H)-one ((*S*)-8h)

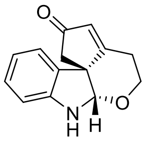

To a 10 mL sample vial containing a 0.1 M CH_3Cl (3 mL) solution of 6-((tert-butyldimethylsilyl)oxy)-1-(1*H*-indol-3-yl)hex-3-yn-2-one **1h** (105 mg, 0.30 mmol) was added chiral silver salt **16** (12 mg, 0.015 mmol) and the reaction stirred under air at –30 °C for 21 h. When complete by TLC, the reaction was concentrated under vacuum and the solvent replaced with THF (3 mL). 10% Aqueous HCl (0.15 mL) was added, and the reaction stirred at RT under air for 0.5 h. The reaction was diluted with EtOAc (20 mL), washed sequentially with saturated aqueous $NaHCO_3$ (10 mL) and brine (10 mL), and the combined aqueous washes back-extracted with EtOAc (5 mL). The combined organic layers were dried ($MgSO_4$), concentrated and purified by column chromatography (silica) eluting with 20–50% EtOAc in hexane, affording the title compound as an off-white solid (42 mg, 60%).

5,6,7a,8-Tetrahydro-1*H*-cyclopenta[3,4]oxepino[2,3-b]indol-2(4*H*)-one (8i)

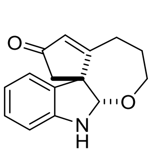

To a 10 mL sample vial containing a 0.1 M CH_2Cl_2 (4.2 mL) solution of 7-((tert-butyldimethylsilyl)oxy)-1-(1*H*-indol-3-yl)hept-3-yn-2-one **1i** (150 mg, 0.42 mmol) was added AgOTf (1.4 mg, 0.005 mmol) and the reaction stirred under air at RT. After 2 h, the reaction was concentrated under vacuum and the

solvent replaced with THF (3 mL) and stirred under air. To the reaction was added a premixed solution of TBAF (1.2 mL, 1.0 M in THF, 1.2 mmol) and acetic acid (72 µL, 1.2 mmol) and the reaction stirred for 21 h. The reaction was diluted with EtOAc (20 mL), washed sequentially with saturated aqueous NH_4Cl (2 x 5 mL), brine (5 mL), dried ($MgSO_4$), and purified by column chromatography (silica) eluting with 50–75% EtOAc in hexane, affording the title compound as an orange oil (66 mg, 65%); R_f 0.50 (66% EtOAc in hexane); ν_{max} (thin film)/cm^{-1} 3356 (br), 2941 (m, NH), 1713 (s), 1686 (s), 1609 (s), 1484 (m), 1045 (m), 747 (s); δ_H (400 MHz, $CDCl_3$) 1.60–1.80 (2H, m), 2.26 (1H, td, $^2J_{HH}$ 12.0, $^3J_{HH}$ 6.0), 2.53 (1H, d, $^2J_{HH}$ 19.0), 2.70–2.77 (3H, m), 3.55 (1H, app. dt, $^2J_{HH}$ 13.0, $^3J_{HH}$ 4.0), 3.87 (1H, ddd, $^2J_{HH}$ 13.0, $^3J_{HH}$ 9.0, $^3J_{HH}$ 3.0), 4.59 (1H, br s), 5.19 (1H, s), 6.17 (1H, s), 6.67 (1H, d, $^3J_{HH}$ 7.5), 6.71 (1H, t, $^3J_{HH}$ 7.5), 6.82 (1H, app. d, $^3J_{HH}$ 7.5), 7.11 (1H, td, $^3J_{HH}$ 7.5, $^4J_{HH}$ 1.5); δ_C (100 MHz, $CDCl_3$) 27.1 (CH_2), 27.4 (CH_2), 51.2 (CH_2), 60.5 (C), 61.0 (CH_2), 96.5 (CH), 107.2 (CH), 118.4 (CH), 121.6 (CH), 127.7 (CH), 129.5 (C), 131.7 (CH), 147.3 (C), 179.3 (C), 206.0 (C=O); HRMS (ESI$^+$): Found: 264.0990; $C_{15}H_{15}NNaO_2$ (MNa^+) Requires 264.0995 (1.8 ppm error), Found: 242.1176; $C_{15}H_{16}NO_2$ (MH^+) Requires 242.1176 (0.0 ppm error).

6-Methyl-5,6,6a,7-tetrahydro-1*H*-cyclopenta[3,4]pyrido[2,3-b]indol-2(4*H*)-one (8j)

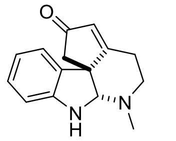

To a 10 mL sample vial containing a 0.1 M CH_2Cl_2 (3 mL) solution of tert-butyl (6-(1*H*-indol-3-yl)-5-oxohex-3-yn-1-yl)(methyl)carbamate **1j** (100 mg, 0.290 mmol) was added AgOTf (2.2 mg, 0.009 mmol) and the reaction stirred under air at RT. After 1 h, TFA (0.3 mL) was added and the reaction stirred for a further 5 h. The reaction was quenched with saturated aqueous $NaHCO_3$ (20 mL), extracted sequentially with CH_2Cl_2 (3 x 10 mL), dried (Na_2SO_4), and purified by column chromatography (silica) eluting with 1–5% MeOH in CH_2Cl_2, affording the title compound as a yellow solid (52 mg, 74%); Mp 117–123 °C R_f 0.15 (3% CH_2Cl_2 in MeOH); ν_{max} (thin film)/cm^{-1} 3356 (Br), 2924 (m), 2852 (m), 1678 (s), 1619 (s), 1603 (s), 1466 (s), 1038 (3), 743 (s); δ_H (400 MHz, $CDCl_3$) 2.38 (3H, s), 2.40–2.48 (1H, m), 2.52 (1H, d, $^2J_{HH}$ 18.5), 2.74 (1H, d, $^2J_{HH}$ 18.5), 2.82–2.92 (1H, m), 4.37 (1H, s), 4.55 (1H, br s), 6.11 (1H, d, (1H, d, $^4J_{HH}$ 1.5), 6.65 (1H, d, $^3J_{HH}$ 7.5), 6.69 (1H, t, $^3J_{HH}$ 7.5), 6.78 (1H, app. d, $^3J_{HH}$ 7.5), 7.07 (1H, td, $^3J_{HH}$ 7.5, $^4J_{HH}$ 1.5); δ_C (100 MHz, $CDCl_3$) 27.4 (CH_2), 41.4 (CH_3), 46.7 (CH_2), 52.6 (CH_2), 56.5 (C), 86.3 (CH), 109.3 (CH), 119.6 (CH), 122.2 (CH), 128.9 (CH), 129.8 (CH), 131.2 (C), 149.7 (C), 178.8 (C), 207.3 (C=O); HRMS (ESI$^+$): Found: 263.1156; $C_{15}H_{16}N_2NaO$ (MNa^+) Requires 263.1155 (–0.3 ppm error), Found: 241.1341; $C_{15}H_{17}N_2O$ (MH^+) Requires 241.1335 (–2.2 ppm error).

1-phenyl-9*H*-carbazole (15)

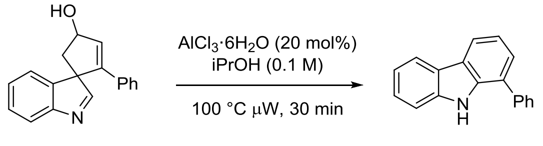

To a 2–5 mL Biotage microwave reaction tube was added a 0.1 M isopropanol solution (1.5 mL) of 2-phenylspiro[cyclopentane-1,3'-indol]-2-en-4-ol **S6** (32 mg, 0.122 mmol), $AlCl_3{\cdot}6H_2O$ (6 mg, 0.024 mmol) and the reaction heated using a microwave under an air atmosphere at 100 °C for 0.5 h. The reaction solution was concentrated by vacuum, yielding the title compound as a dark blue oil (33 mg, 100%) with data consistent with the literature data.[2] R_f 0.90 (50% EtOAc in hexane); δ_H (400 MHz, $CDCl_3$) 7.30 (1H, ddd, $^3J_{HH}$ 7.5, $^3J_{HH}$ 7.5, $^4J_{HH}$ 1.0), 7.38 (1H, dd, $^3J_{HH}$ 7.5, $^3J_{HH}$ 7.5), 7.40–7.52 (4H, m),

7.60 (2H, dd, $^3J_{HH}$ 8.0, $^3J_{HH}$ 7.5), 7.73 (2H, d, $^3J_{HH}$ 7.5), 8.13 (1H, d, $^3J_{HH}$ 7.5), 8.16 (1H, d, $^3J_{HH}$ 7.5), 8.33 (1H, br s).

Table S1 Solvent study on the conversion of **3a** to **7a**.

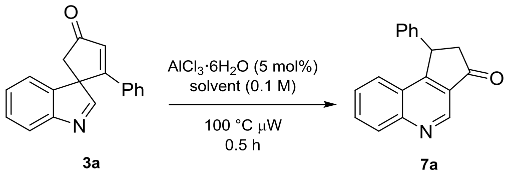

Solvent (0.1 M)	Conversion **3a** to **7a** (%)
isopropanol	100
EtOH	100
MeCN	15
THF	15
Tol	2

% conversion based on ^{1}H-NMR integrations

Table S2 Catalyst study on the conversion of **3a** to **7a**.

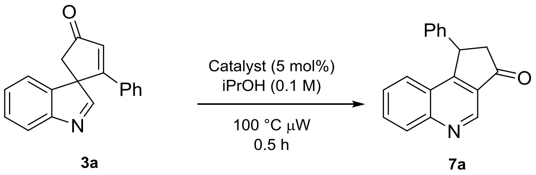

Catalyst (5 mol%)	Conversion **3a** to **7a** (%)
$AlCl_3 \cdot 6H_2O$	100
$Al(iPrO)_3$	10
$Yb(OTf)_3$	100
$Sn(OTf)_2$	90
$SnCl_2 \cdot 2H_2O$	75
$MgCl_2$	40
$Cu(OTf)_2$	75
AgOTf	65
$Sc(OTf)_3$	100

% conversion based on ^{1}H-NMR integrations

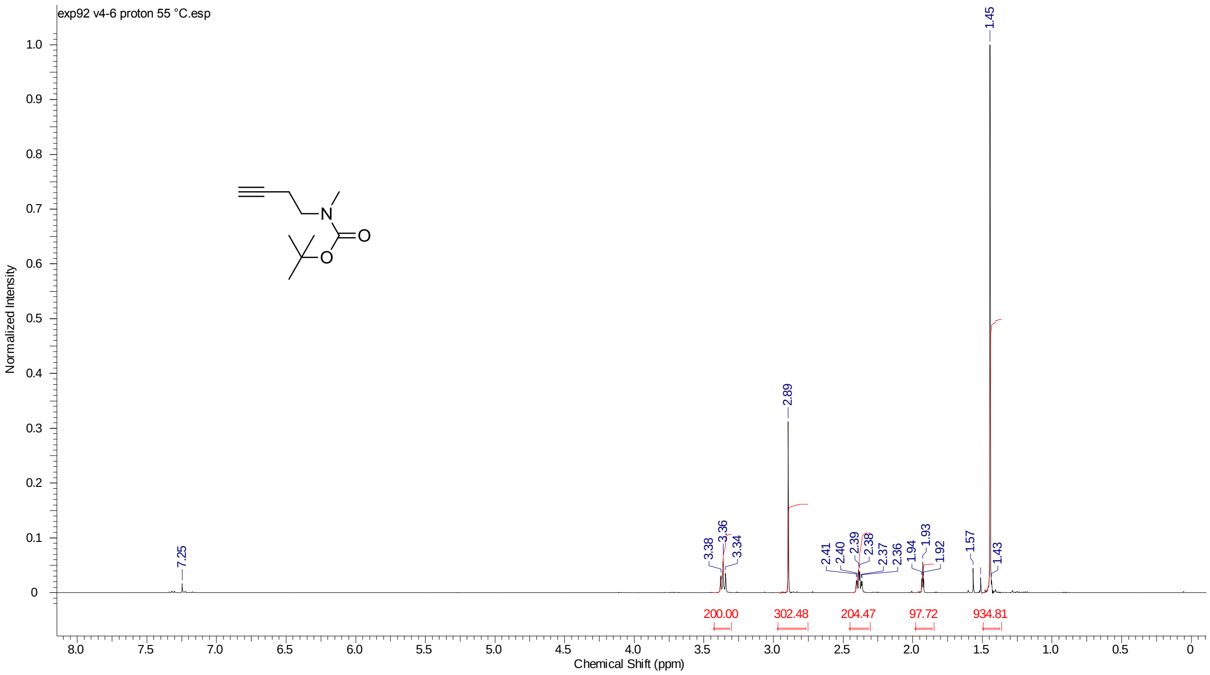
exp92 v4-6 proton 55 °C.esp
N
O
O
Normalized Intensity
Chemical Shift (ppm)
7.25
3.38
3.36
3.34
2.89
2.41
2.40
2.39
2.38
2.37
2.36
1.94
1.93
1.92
1.57
1.45
1.43
200.00
302.48
204.47
97.72
934.81

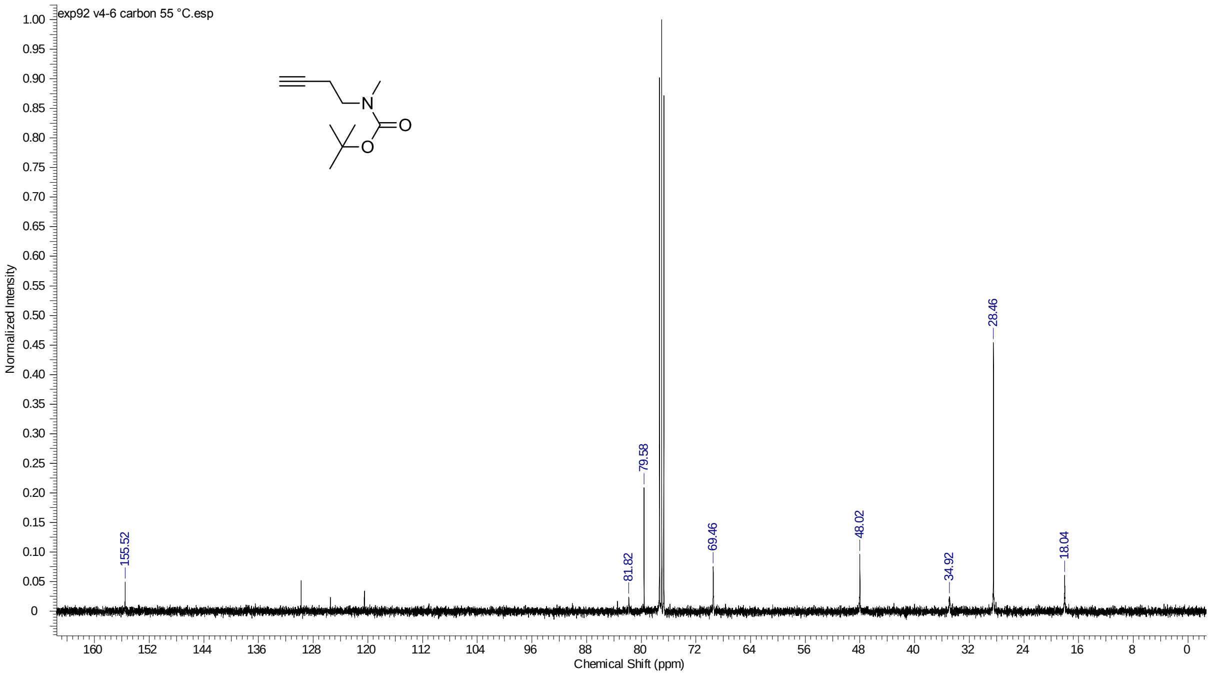
exp92 v4-6 carbon 55 °C.esp
N
O
O
Normalized Intensity
Chemical Shift (ppm)
155.52
81.82
79.58
69.46
48.02
34.92
28.46
18.04

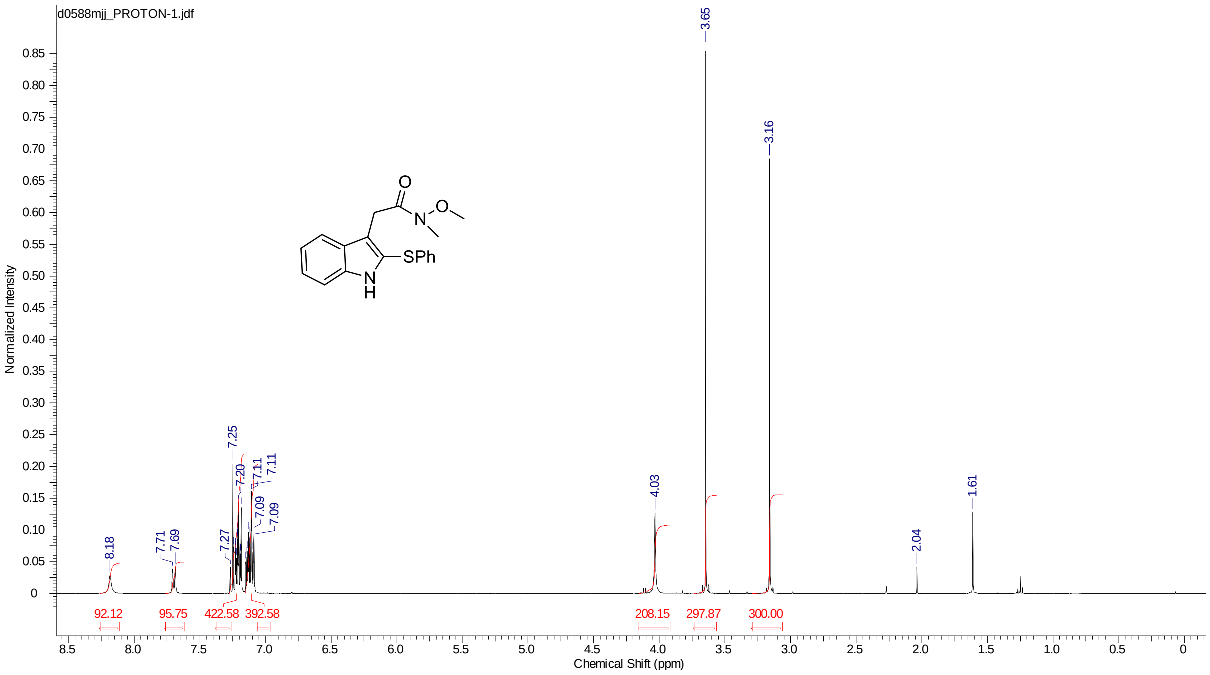
d0588mjj_PROTON-1.jdf
Normalized Intensity
Chemical Shift (ppm)
SPh
N
H
O
N
O
8.18
7.71
7.69
7.27
7.25
7.20
7.11
7.11
7.09
7.09
4.03
3.65
3.16
2.04
1.61
92.12
95.75
422.58
392.58
208.15
297.87
300.00

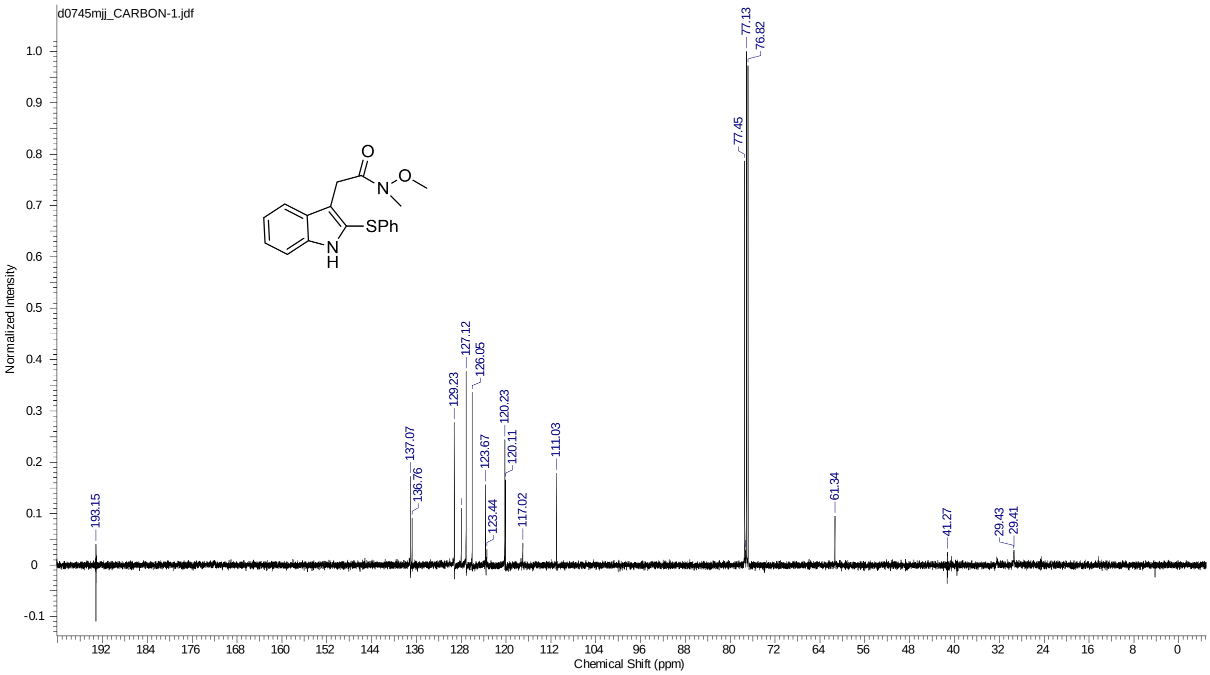
d0745mjj_CARBON-1.jdf
Normalized Intensity
Chemical Shift (ppm)
SPh
N
H
O
N
O
193.15
137.07
136.76
129.23
127.12
126.05
123.67
123.44
120.23
120.11
117.02
111.03
77.45
77.13
76.82
61.34
41.27
29.43
29.41

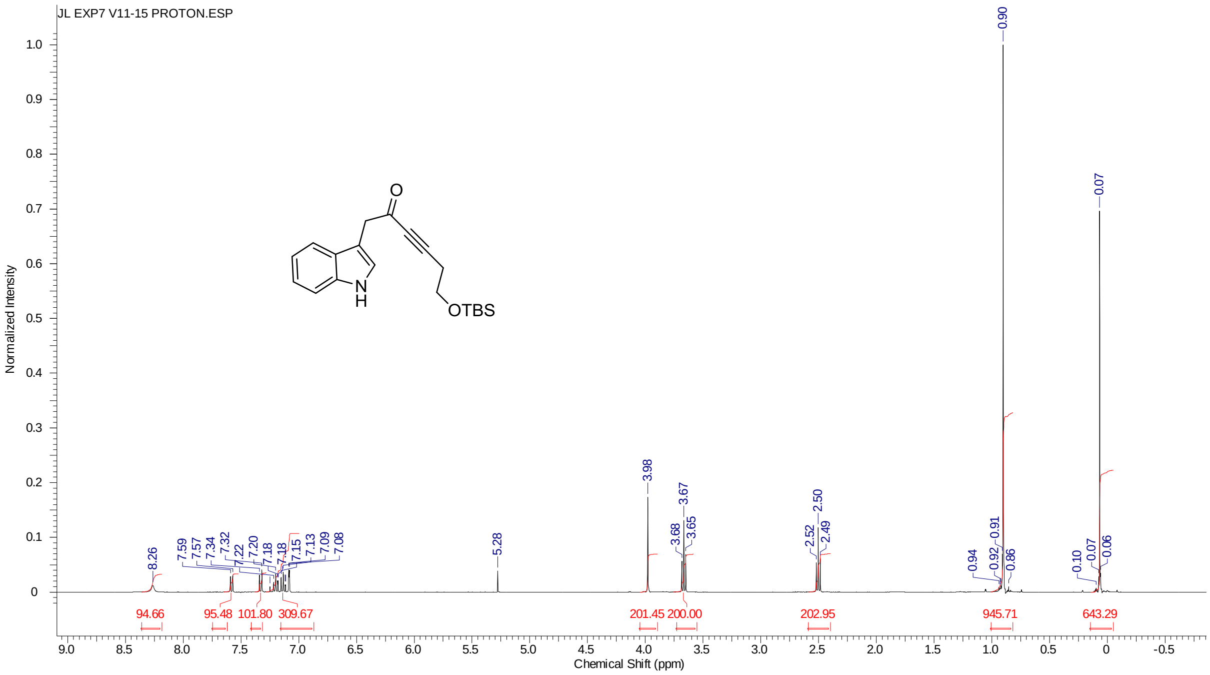
JL EXP7 V11-15 PROTON.ESP
O
N
H
OTBS
Normalized Intensity
Chemical Shift (ppm)
0.90
0.07
3.98
3.67
3.68
3.65
2.50
2.52
2.49
5.28
8.26
7.59
7.57
7.34
7.32
7.22
7.20
7.18
7.18
7.15
7.13
7.09
7.08
0.94
0.92
0.91
0.86
0.10
0.07
0.06
94.66
95.48
101.80
309.67
201.45
200.00
202.95
945.71
643.29

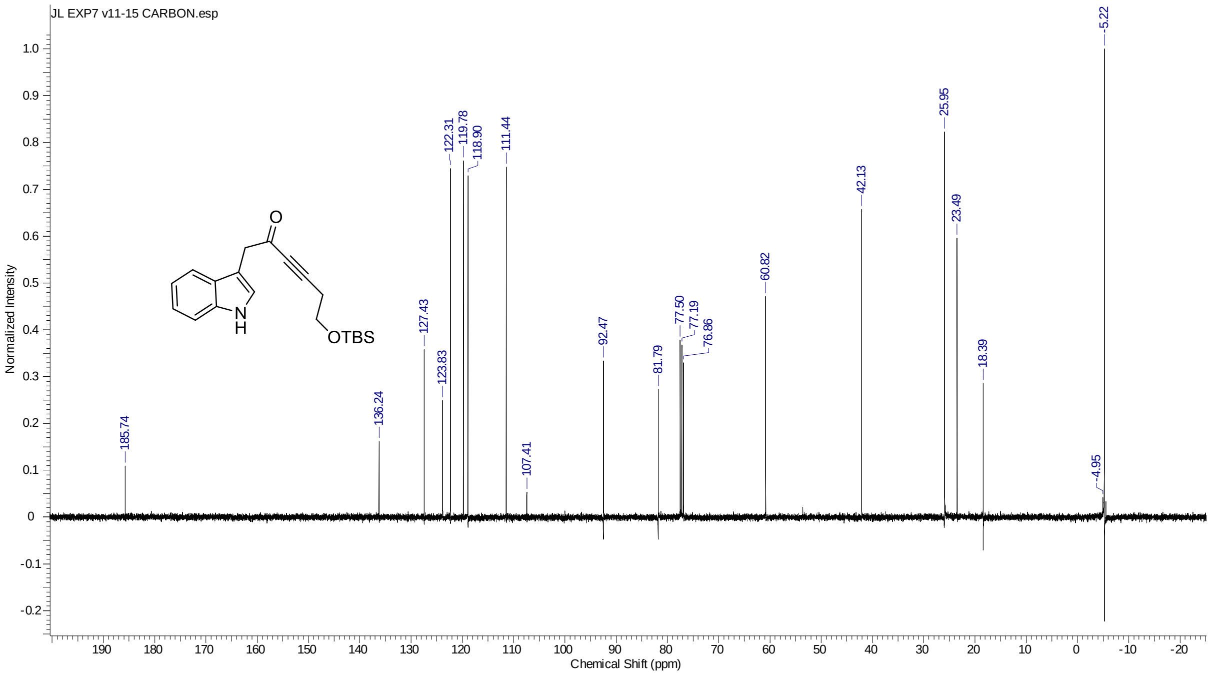
JL EXP7 v11-15 CARBON.esp
O
N
H
OTBS
Normalized Intensity
Chemical Shift (ppm)
185.74
136.24
127.43
123.83
122.31
119.78
118.90
111.44
107.41
92.47
81.79
77.50
77.19
76.86
60.82
42.13
25.95
23.49
18.39
-4.95
-5.22

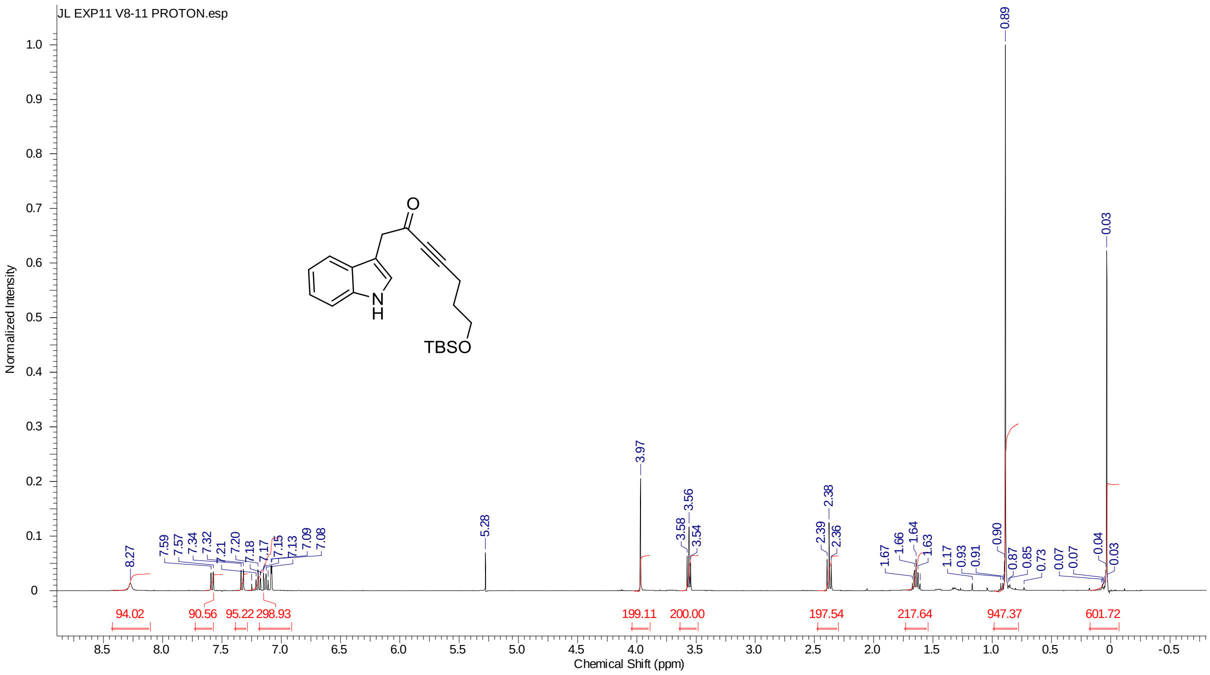
JL EXP11 V8-11 PROTON.esp
Normalized Intensity
Chemical Shift (ppm)
O
N
H
TBSO
8.27
7.59
7.57
7.34
7.32
7.21
7.20
7.18
7.17
7.15
7.13
7.09
7.08
5.28
3.97
3.58
3.56
3.54
2.39
2.38
2.36
1.67
1.66
1.64
1.63
1.17
0.93
0.91
0.90
0.89
0.87
0.85
0.73
0.07
0.07
0.04
0.03
0.03
94.02
90.56
95.22
298.93
199.11
200.00
197.54
217.64
947.37
601.72

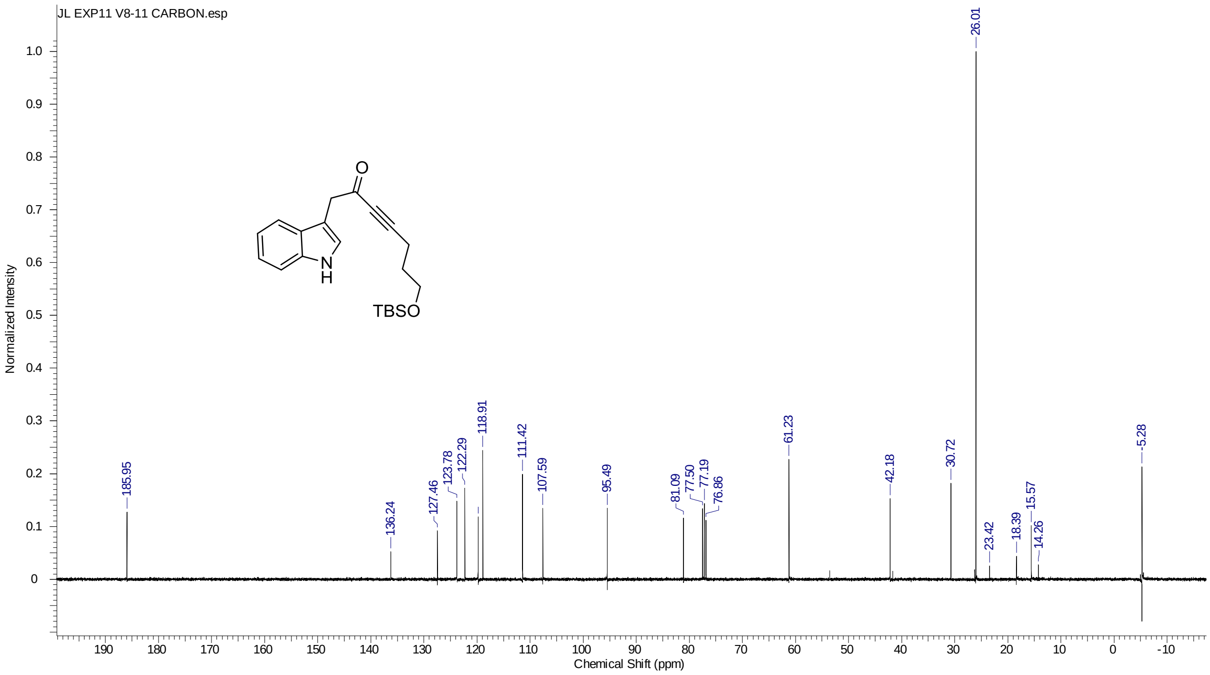
JL EXP11 V8-11 CARBON.esp
Normalized Intensity
Chemical Shift (ppm)
O
N
H
TBSO
185.95
136.24
127.46
123.78
122.29
118.91
111.42
107.59
95.49
81.09
77.50
77.19
76.86
61.23
42.18
30.72
26.01
23.42
18.39
15.57
14.26
-5.28

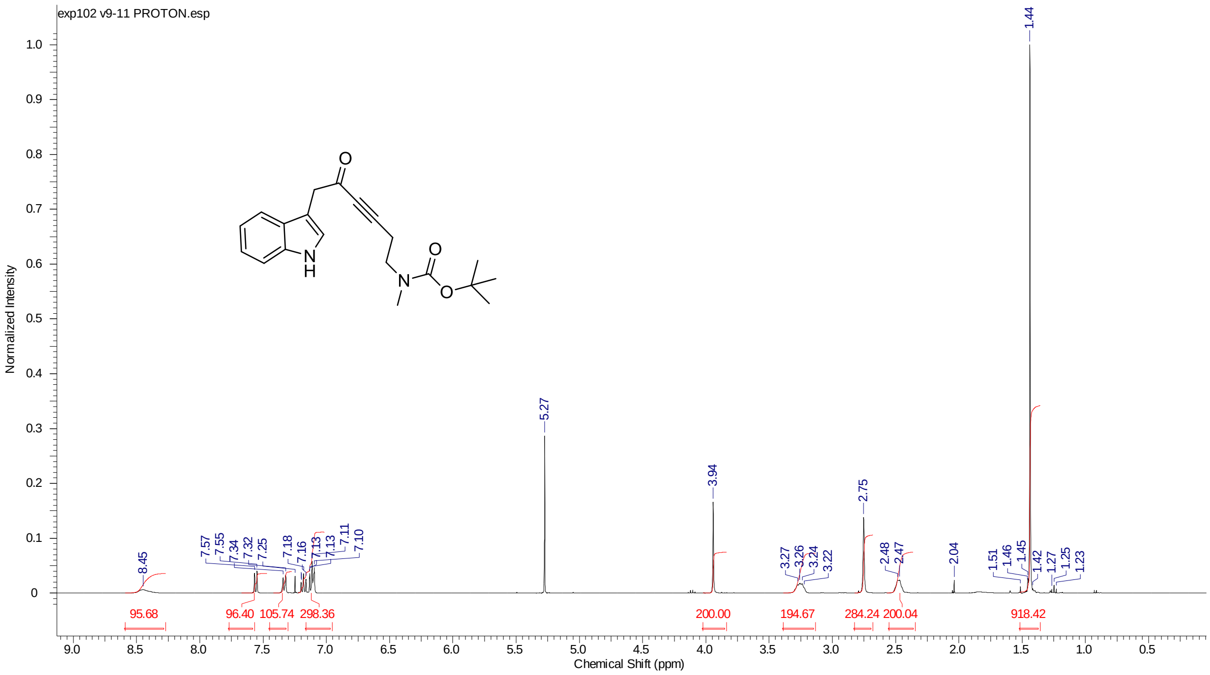
exp102 v9-11 PROTON.esp
Normalized Intensity
Chemical Shift (ppm)
8.45
7.57
7.55
7.34
7.32
7.25
7.18
7.16
7.13
7.13
7.11
7.10
5.27
3.94
3.27
3.26
3.24
3.22
2.75
2.48
2.47
2.04
1.51
1.46
1.45
1.44
1.42
1.27
1.25
1.23
95.68
96.40
105.74
298.36
200.00
194.67
284.24
200.04
918.42
O
N
H
N
O
O

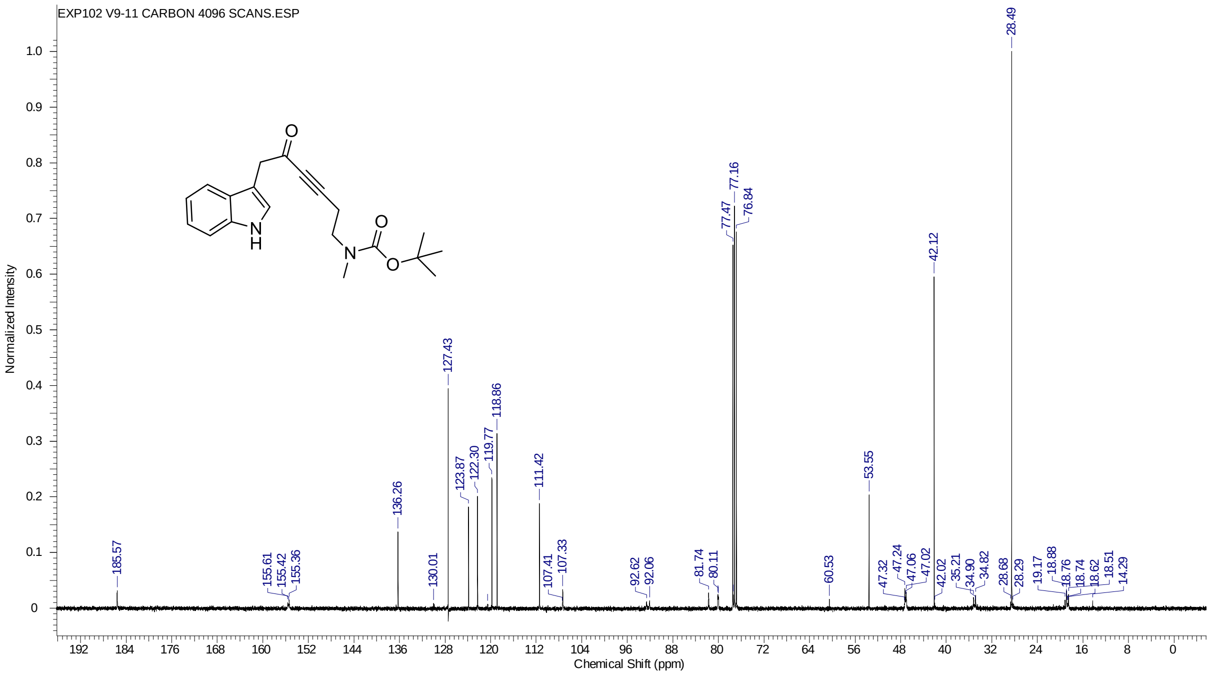
EXP102 V9-11 CARBON 4096 SCANS.ESP
Normalized Intensity
Chemical Shift (ppm)
185.57
155.61
155.42
155.36
136.26
130.01
127.43
123.87
122.30
119.77
118.86
111.42
107.41
107.33
92.62
92.06
81.74
80.11
77.47
77.16
76.84
60.53
53.55
47.32
47.24
47.06
47.02
42.12
42.02
35.21
34.90
34.82
28.68
28.49
28.29
19.17
18.88
18.76
18.74
18.62
18.51
14.29
O
N
H
N
O
O

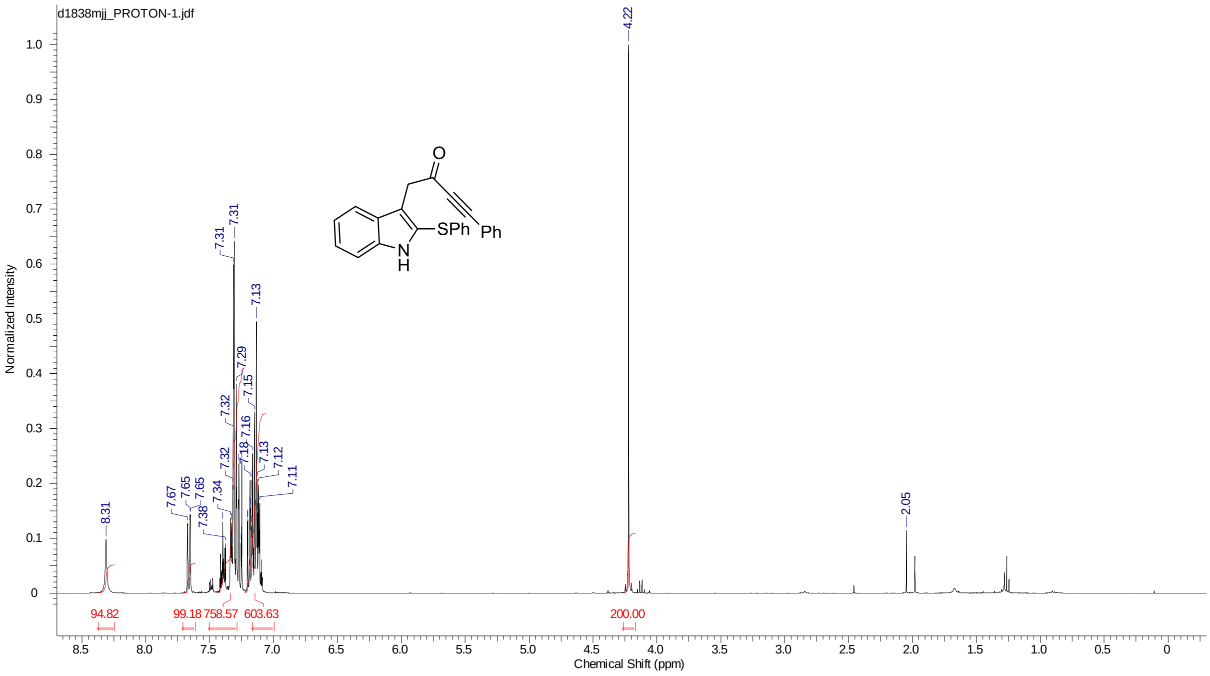
d1838mjj_PROTON-1.jdf
Normalized Intensity
Chemical Shift (ppm)
SPh
Ph
N
H
O

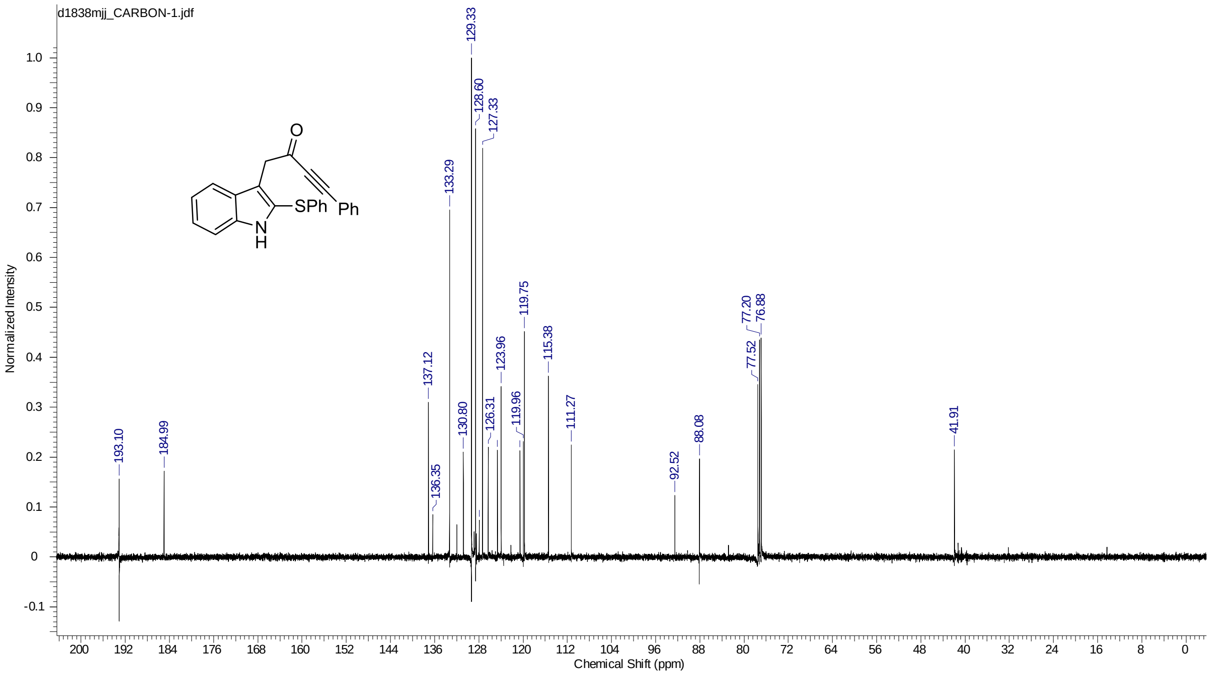
d1838mjj_CARBON-1.jdf
Normalized Intensity
Chemical Shift (ppm)
SPh
Ph
N
H
O

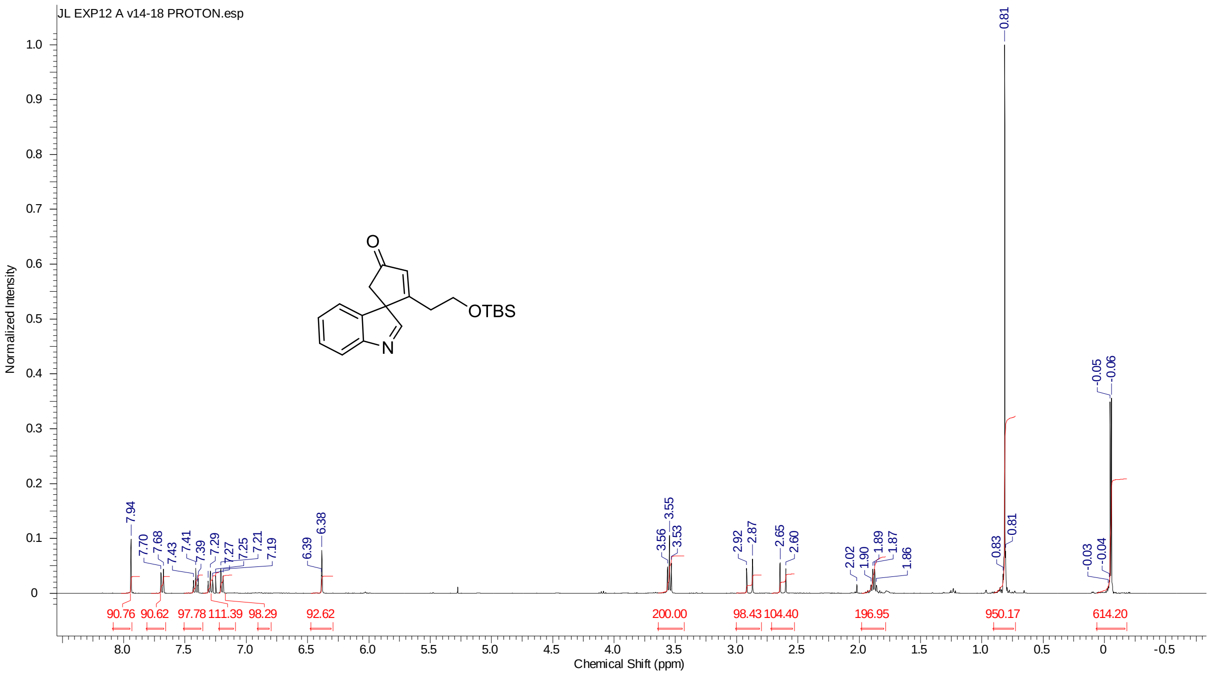
JL EXP12 A v14-18 PROTON.esp
Normalized Intensity
Chemical Shift (ppm)
OTBS

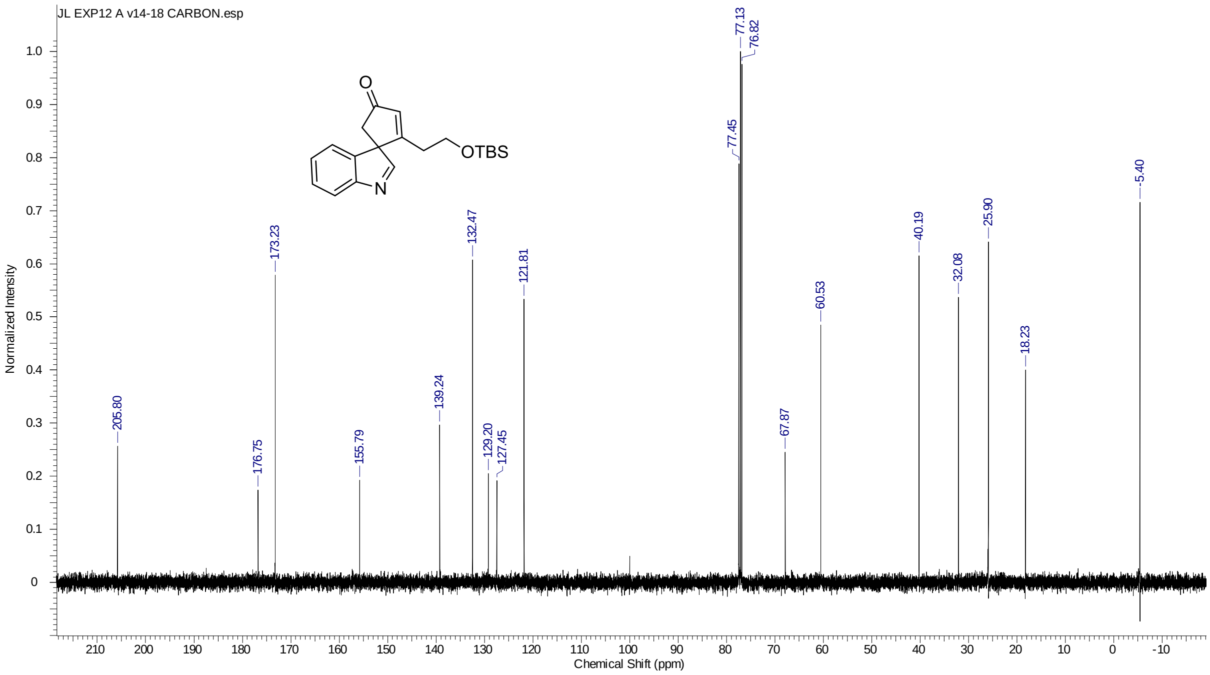
JL EXP12 A v14-18 CARBON.esp
Normalized Intensity
Chemical Shift (ppm)
OTBS

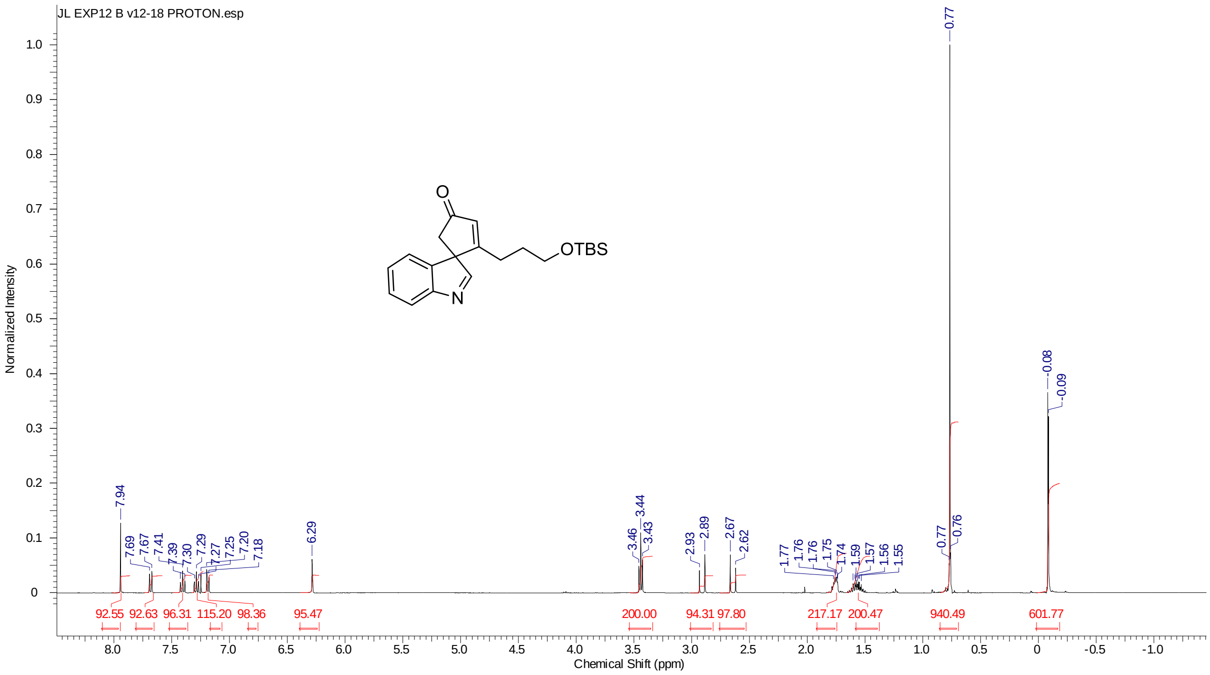
JL EXP12 B v12-18 PROTON.esp
Normalized Intensity
Chemical Shift (ppm)
OTBS
O
N
7.94
7.69
7.67
7.41
7.39
7.30
7.29
7.27
7.25
7.20
7.18
6.29
3.46
3.44
3.43
2.93
2.89
2.67
2.62
1.77
1.76
1.76
1.75
1.74
1.59
1.57
1.56
1.55
0.77
0.77
0.76
-0.08
-0.09
92.55
92.63
96.31
115.20
98.36
95.47
200.00
94.31
97.80
217.17
200.47
940.49
601.77

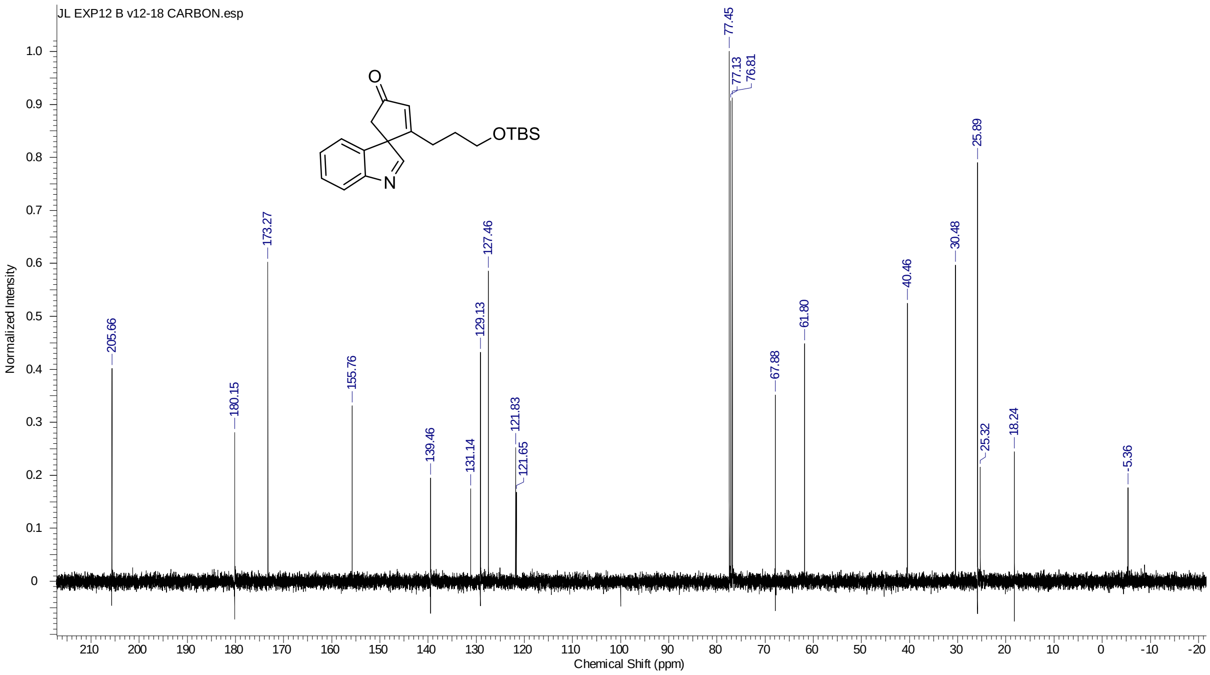
JL EXP12 B v12-18 CARBON.esp
Normalized Intensity
Chemical Shift (ppm)
OTBS
O
N
205.66
180.15
173.27
155.76
139.46
131.14
129.13
127.46
121.83
121.65
77.45
77.13
76.81
67.88
61.80
40.46
30.48
25.89
25.32
18.24
-5.36

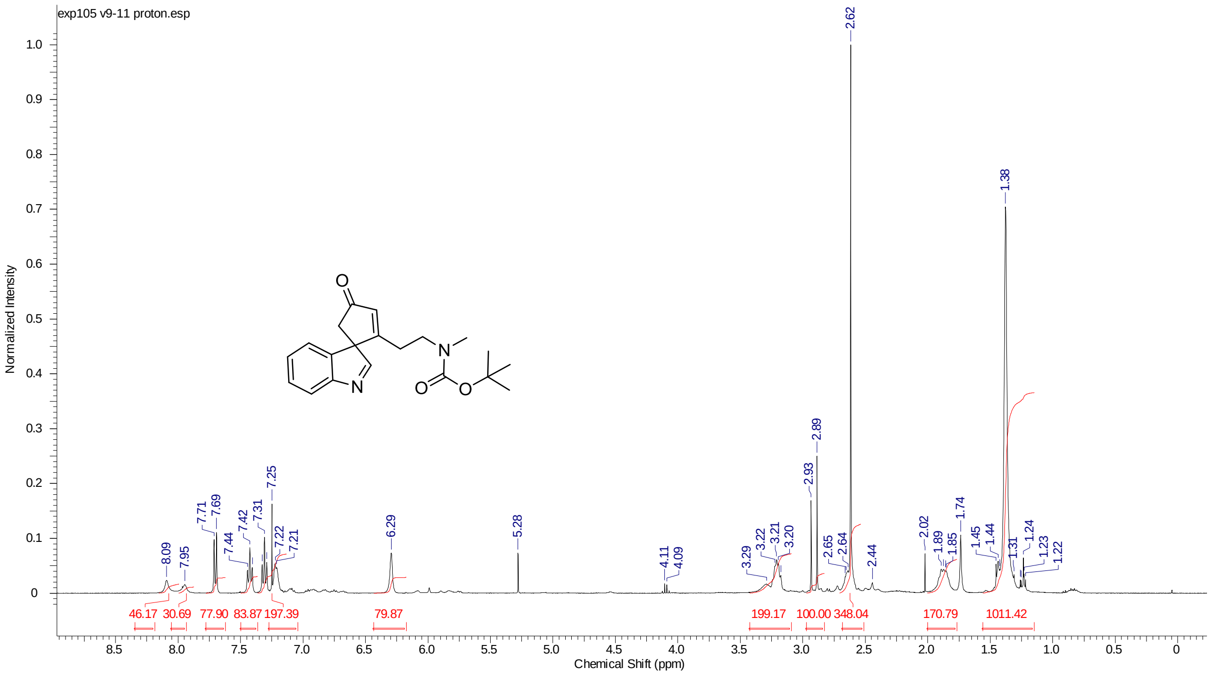
exp105 v9-11 proton.esp
Normalized Intensity
Chemical Shift (ppm)
8.09 7.95 7.71 7.69 7.44 7.42 7.31 7.25 7.22 7.21 6.29 5.28 4.11 4.09 3.29 3.22 3.21 3.20 2.93 2.89 2.65 2.64 2.62 2.44 2.02 1.89 1.85 1.74 1.45 1.44 1.38 1.31 1.24 1.23 1.22
46.17 30.69 77.90 83.87 197.39 79.87 199.17 100.00 348.04 170.79 1011.42
O
N
O
O
N

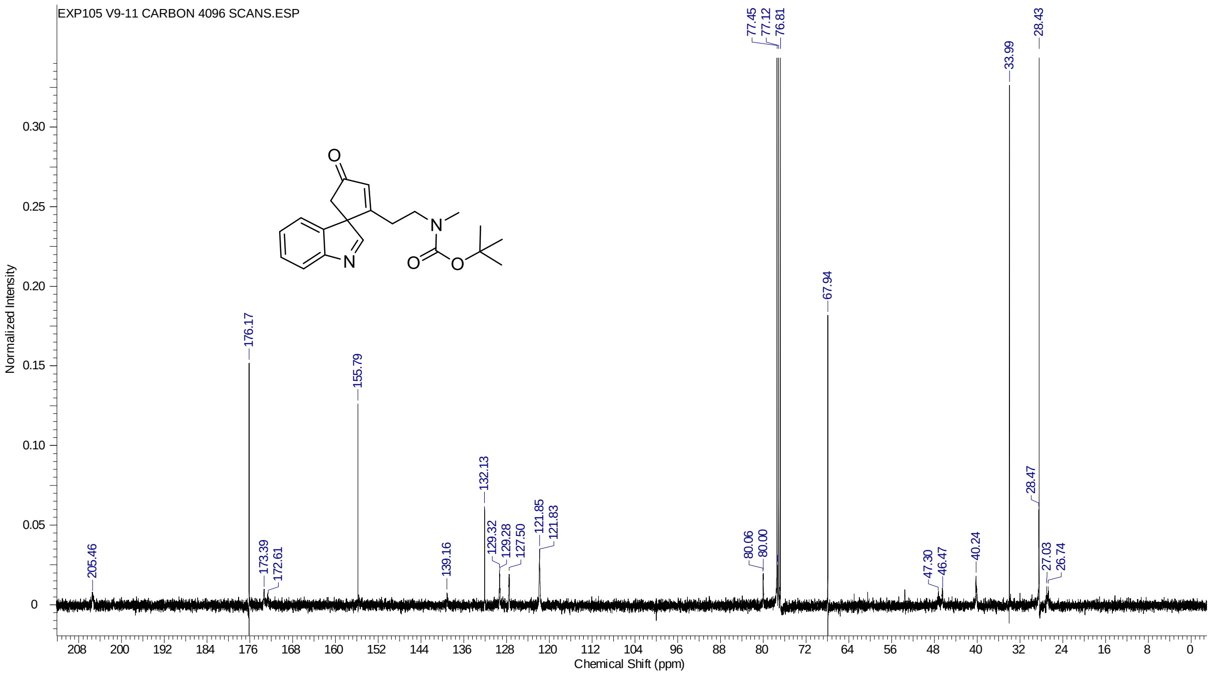
EXP105 V9-11 CARBON 4096 SCANS.ESP
Normalized Intensity
Chemical Shift (ppm)
205.46 176.17 173.39 172.61 155.79 139.16 132.13 129.32 129.28 127.50 121.85 121.83 80.06 80.00 77.45 77.12 76.81 67.94 47.30 46.47 40.24 33.99 28.47 28.43 27.03 26.74
O
N
O
O
N

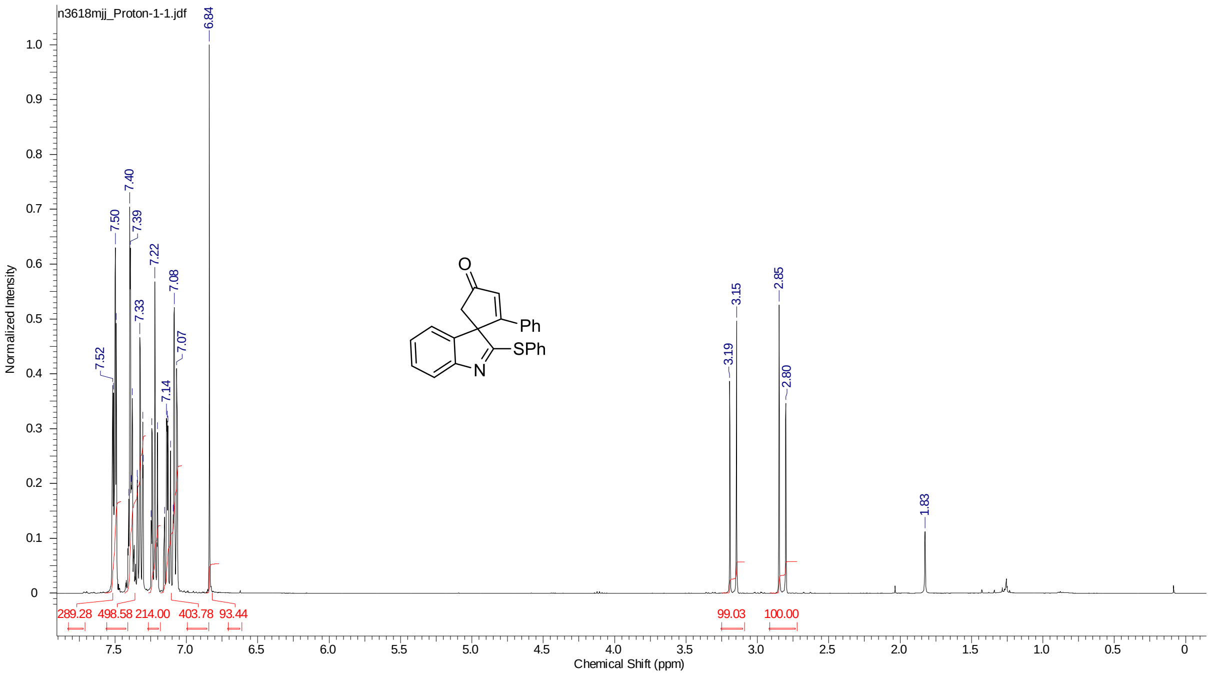
n3618mjj_Proton-1-1.jdf
Normalized Intensity
Chemical Shift (ppm)
7.52
7.50
7.40
7.39
7.33
7.22
7.14
7.08
7.07
6.84
3.19
3.15
2.85
2.80
1.83
289.28
498.58
214.00
403.78
93.44
99.03
100.00
O
Ph
SPh
N

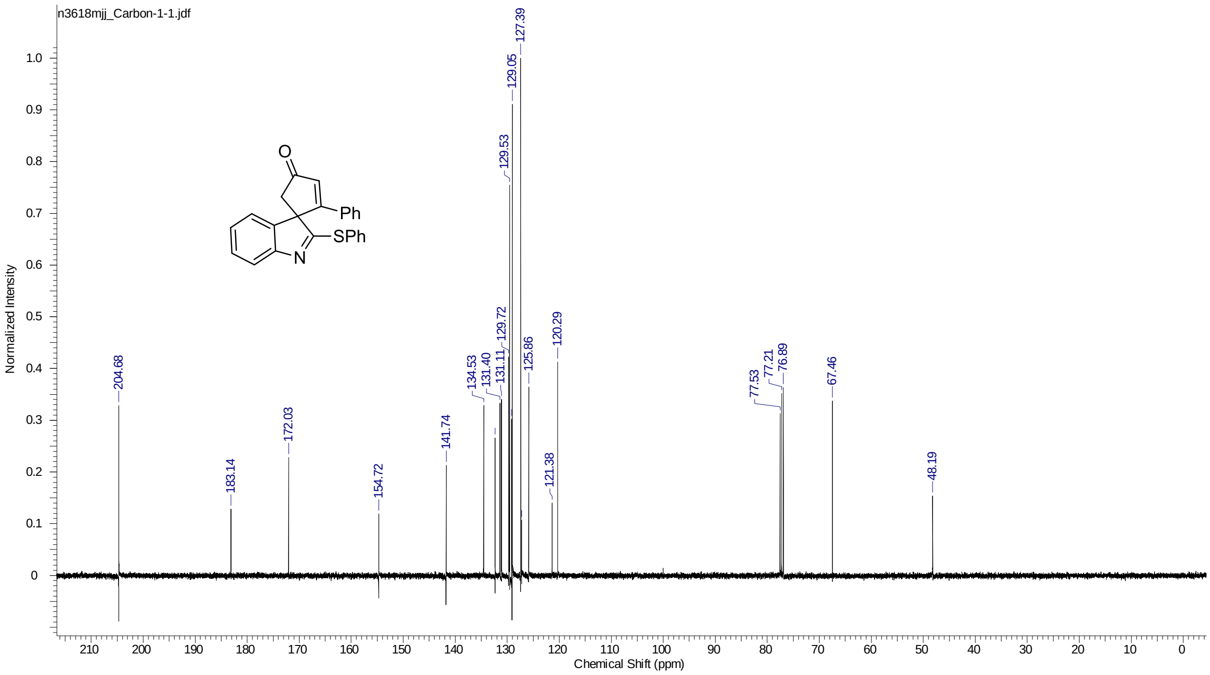
n3618mjj_Carbon-1-1.jdf
Normalized Intensity
Chemical Shift (ppm)
204.68
183.14
172.03
154.72
141.74
134.53
131.40
131.11
129.72
129.53
129.05
127.39
125.86
121.38
120.29
77.53
77.21
76.89
67.46
48.19
O
Ph
SPh
N

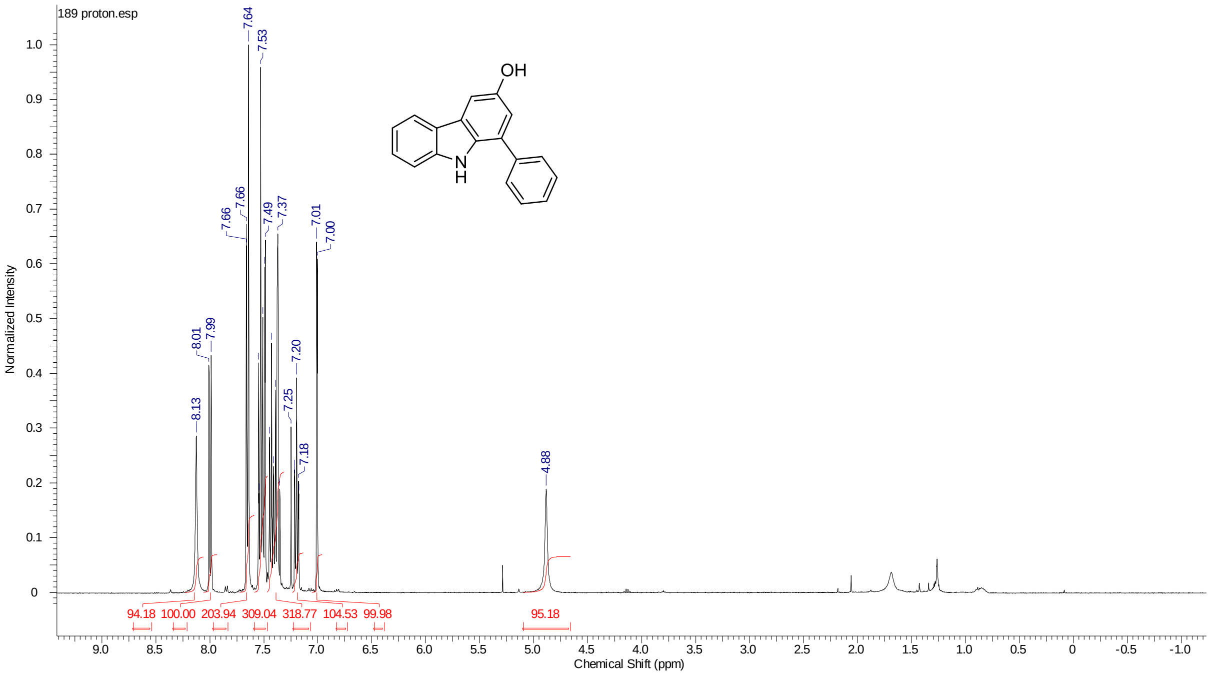
189 proton.esp
OH
N
H
Normalized Intensity
Chemical Shift (ppm)
8.13
8.01
7.99
7.66
7.66
7.64
7.53
7.49
7.37
7.25
7.20
7.18
7.01
7.00
4.88
94.18
100.00
203.94
309.04
318.77
104.53
99.98
95.18

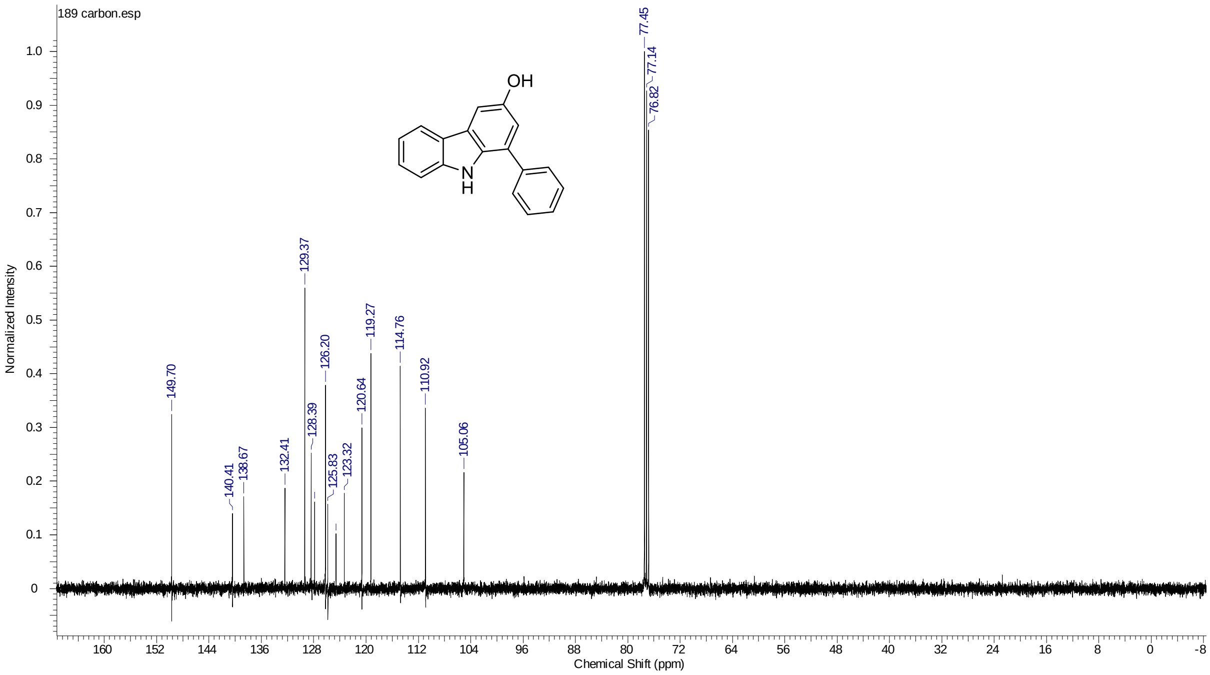
189 carbon.esp
OH
N
H
Normalized Intensity
Chemical Shift (ppm)
149.70
140.41
138.67
132.41
129.37
128.39
126.20
125.83
123.32
120.64
119.27
114.76
110.92
105.06
77.45
77.14
76.82

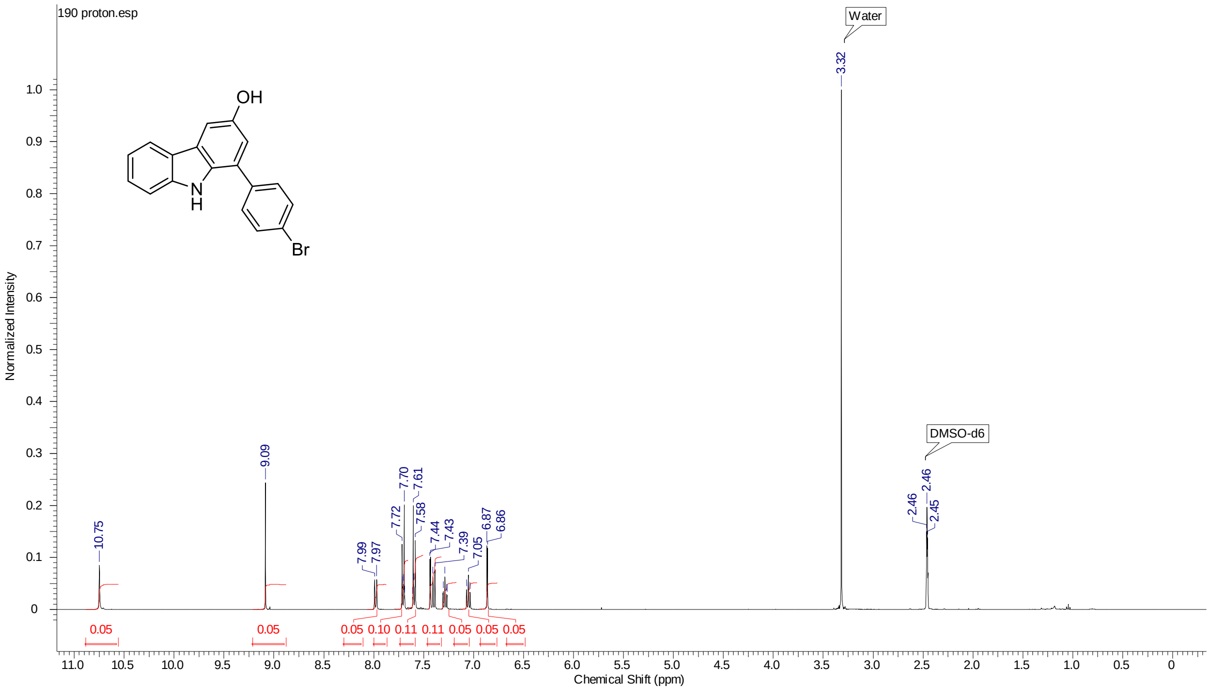
190 proton.esp
Water
DMSO-d6
Normalized Intensity
Chemical Shift (ppm)
OH
N
H
Br
10.75
9.09
7.99
7.97
7.72
7.70
7.61
7.58
7.44
7.43
7.39
7.05
6.87
6.86
3.32
2.46
2.46
2.45
0.05
0.05
0.05
0.10
0.11
0.11
0.05
0.05
0.05

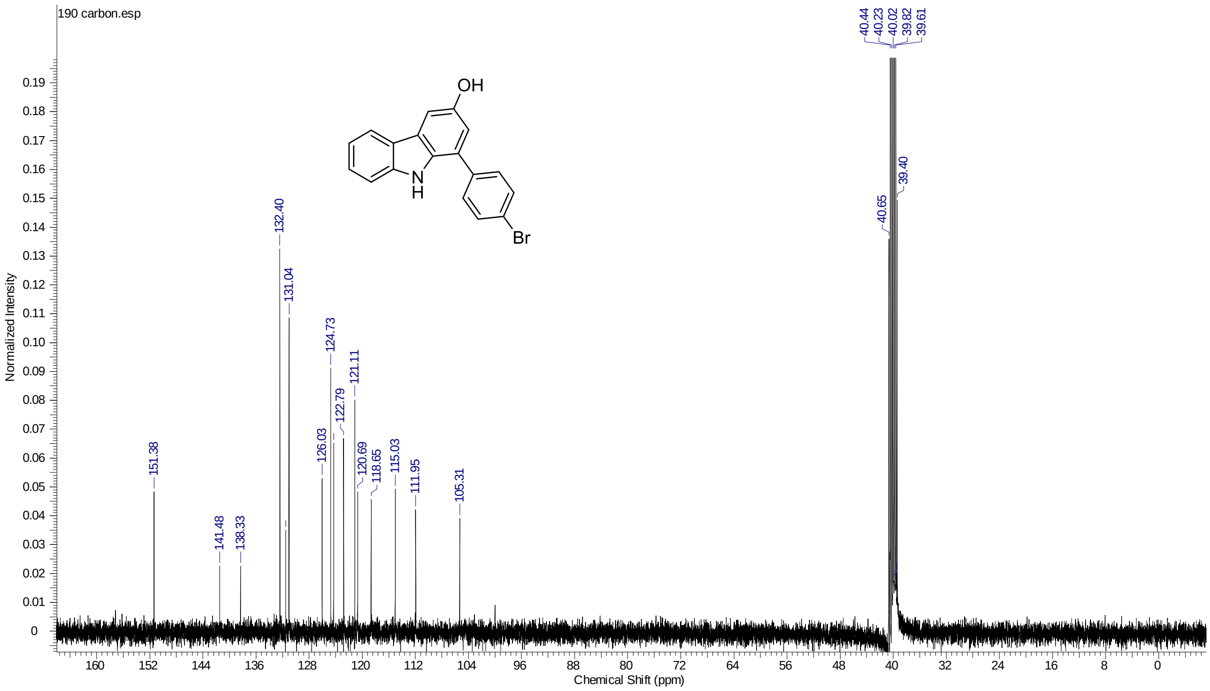
190 carbon.esp
Normalized Intensity
Chemical Shift (ppm)
OH
N
H
Br
151.38
141.48
138.33
132.40
131.04
126.03
124.73
122.79
121.11
120.69
118.65
115.03
111.95
105.31
40.65
40.44
40.23
40.02
39.82
39.61
39.40

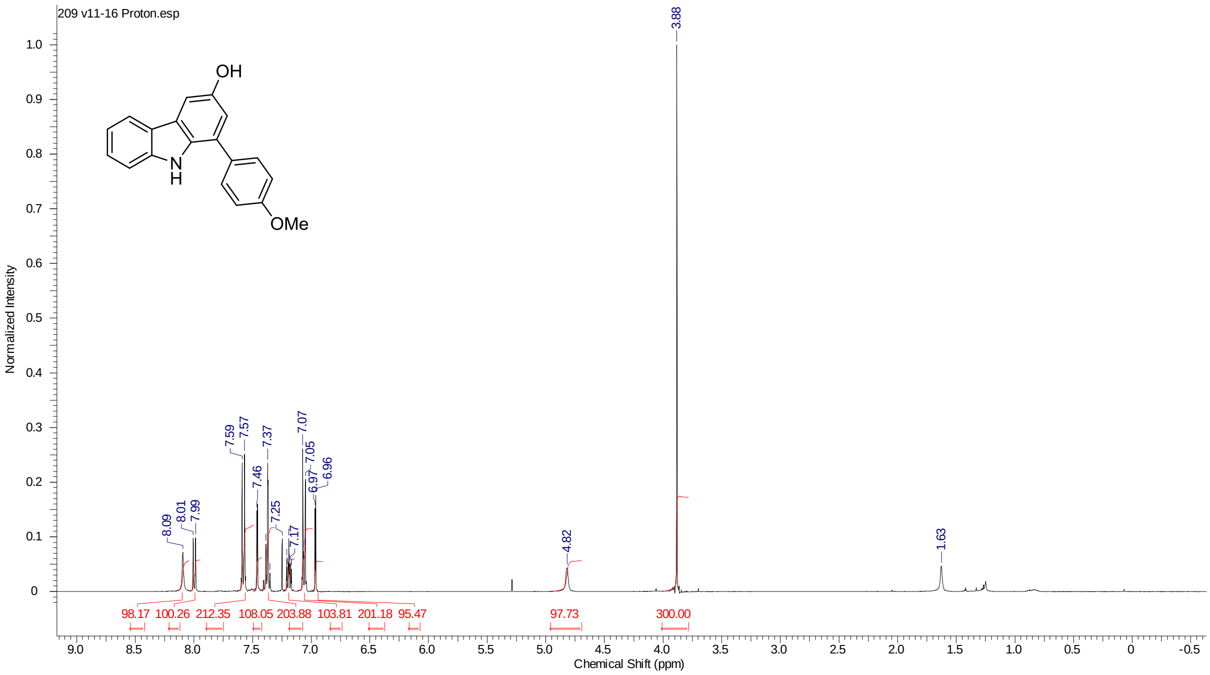
209 v11-16 Proton.esp
OH
N
H
OMe
Normalized Intensity
Chemical Shift (ppm)
3.88
7.59
7.57
7.46
7.37
7.25
7.17
7.07
7.05
6.97
6.96
8.09
8.01
7.99
4.82
1.63
98.17
100.26
212.35
108.05
203.88
103.81
201.18
95.47
97.73
300.00

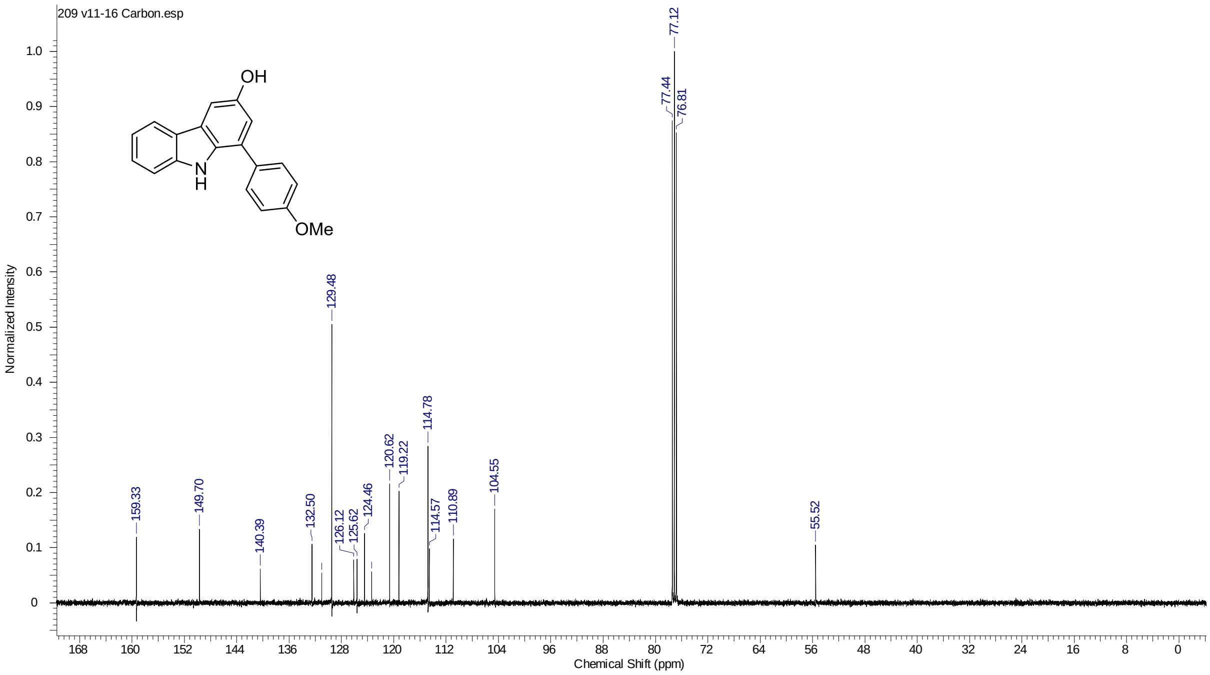
209 v11-16 Carbon.esp
OH
N
H
OMe
Normalized Intensity
Chemical Shift (ppm)
77.12
77.44
76.81
129.48
114.78
120.62
119.22
104.55
159.33
149.70
140.39
132.50
126.12
125.62
124.46
114.57
110.89
55.52

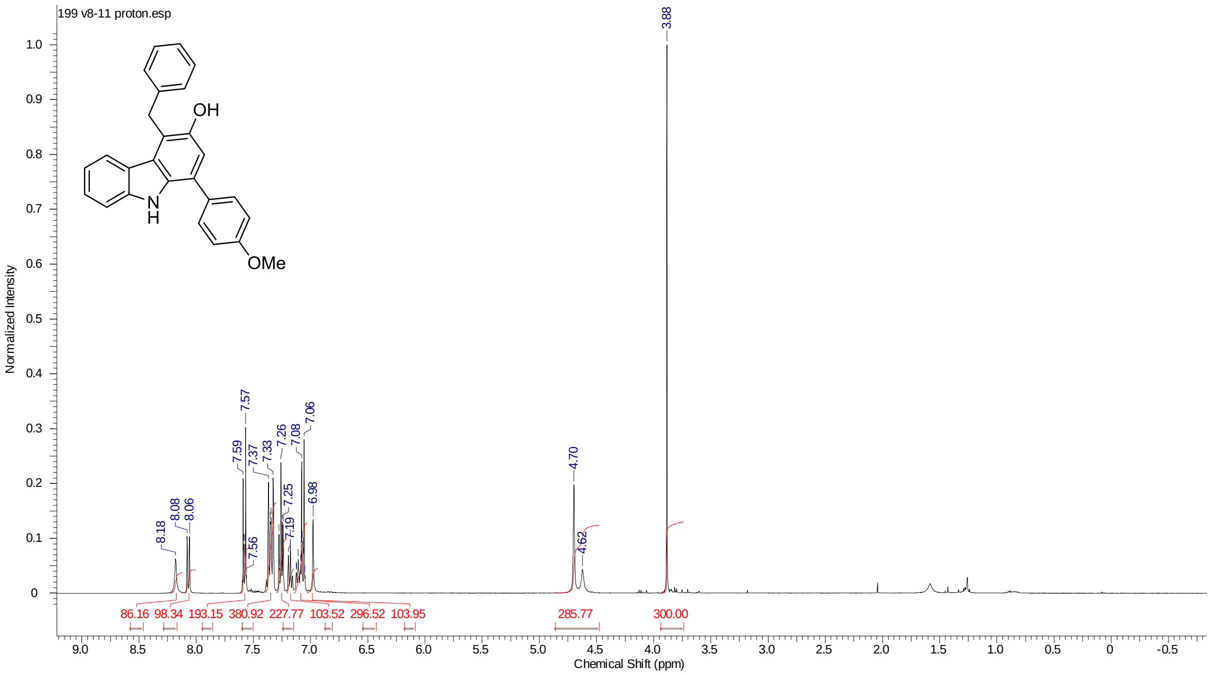
199 v8-11 proton.esp
Normalized Intensity
Chemical Shift (ppm)
OH
N
H
OMe
3.88
7.57
7.59
7.37
7.33
7.26
7.08
7.06
7.25
7.19
6.98
8.18
8.08
8.06
7.56
4.70
4.62
86.16
98.34
193.15
380.92
227.77
103.52
296.52
103.95
285.77
300.00

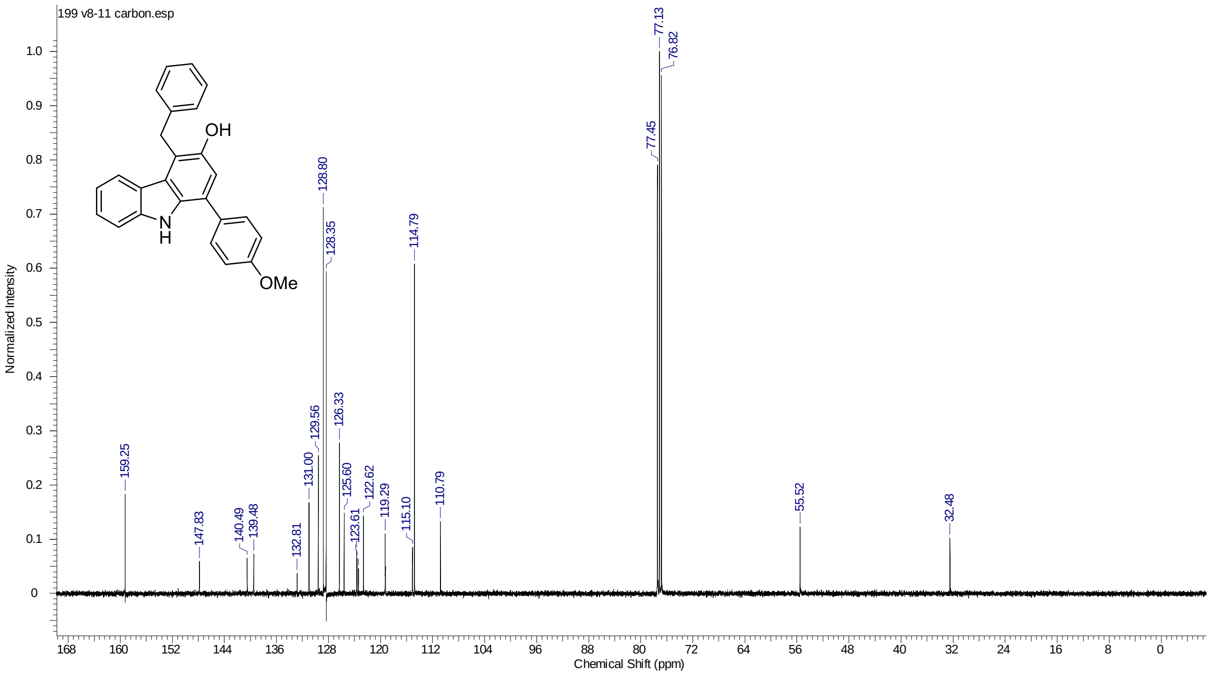
199 v8-11 carbon.esp
Normalized Intensity
Chemical Shift (ppm)
OH
N
H
OMe
159.25
147.83
140.49
139.48
132.81
131.00
129.56
128.80
128.35
126.33
125.60
123.61
122.62
119.29
115.10
114.79
110.79
77.45
77.13
76.82
55.52
32.48

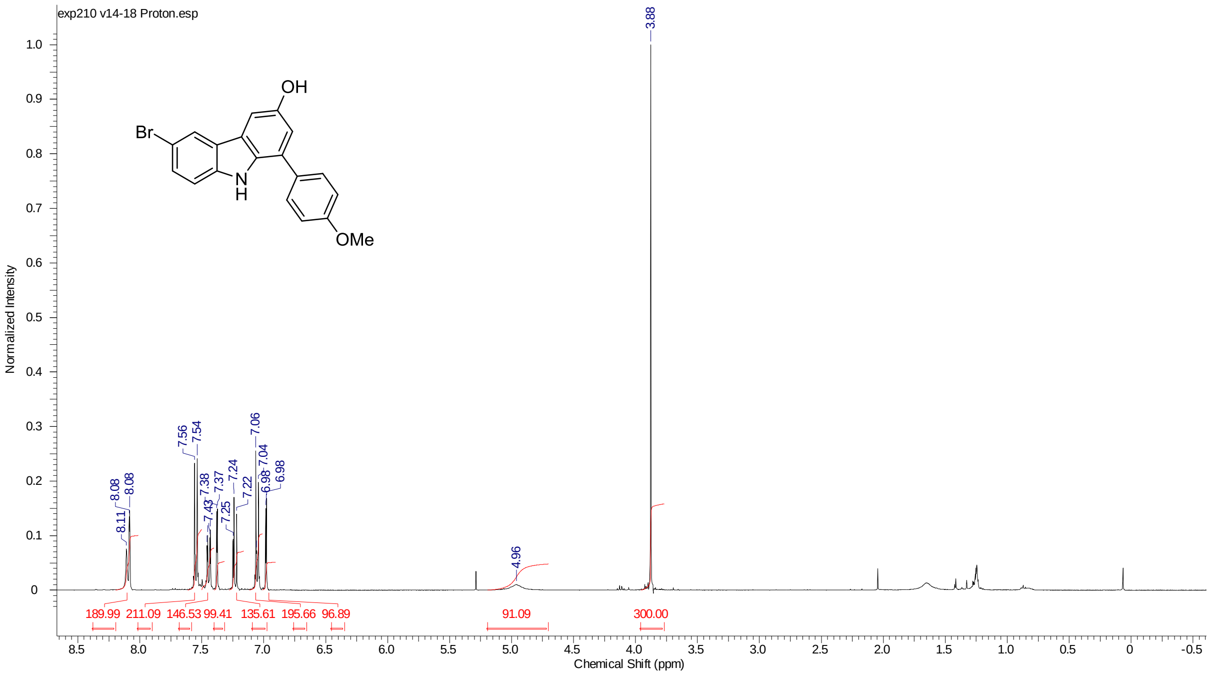
exp210 v14-18 Proton.esp
Normalized Intensity
Chemical Shift (ppm)
OH
Br
N
H
OMe
3.88
8.11
8.08
8.08
7.56
7.54
7.43
7.38
7.37
7.25
7.24
7.22
7.06
7.04
6.98
6.98
4.96
189.99
211.09
146.53
99.41
135.61
195.66
96.89
91.09
300.00

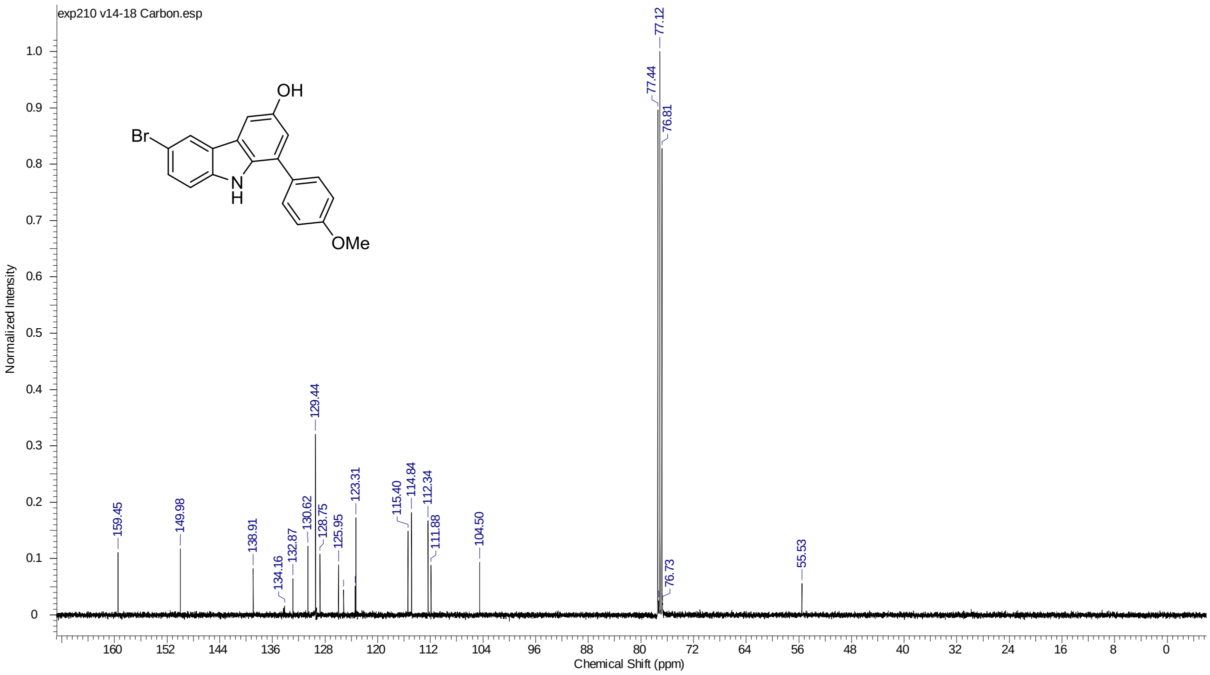
exp210 v14-18 Carbon.esp
Normalized Intensity
Chemical Shift (ppm)
OH
Br
N
H
OMe
77.12
77.44
76.81
76.73
159.45
149.98
138.91
134.16
132.87
130.62
129.44
128.75
125.95
123.31
115.40
114.84
112.34
111.88
104.50
55.53

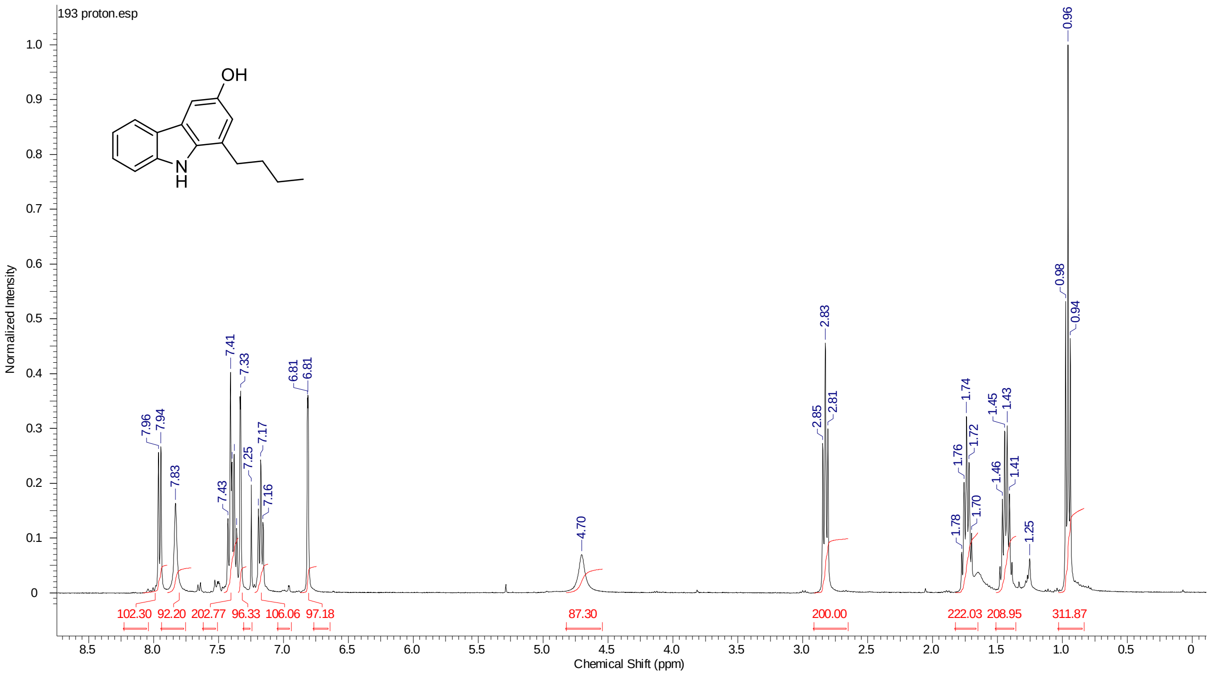
193 proton.esp
OH
N
H
Normalized Intensity
Chemical Shift (ppm)
7.96
7.94
7.83
7.43
7.41
7.33
7.25
7.17
7.16
6.81
6.81
4.70
2.85
2.83
2.81
1.78
1.76
1.74
1.72
1.70
1.46
1.45
1.43
1.41
1.25
0.98
0.96
0.94
102.30
92.20
202.77
96.33
106.06
97.18
87.30
200.00
222.03
208.95
311.87

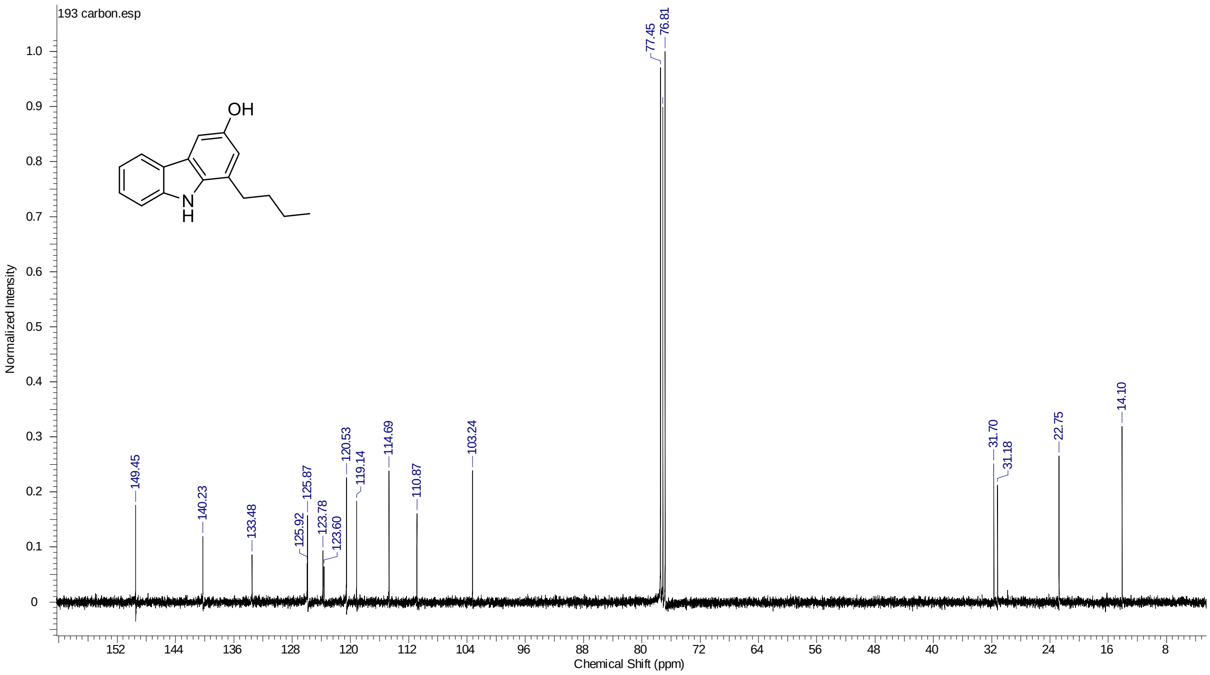
193 carbon.esp
OH
N
H
Normalized Intensity
Chemical Shift (ppm)
149.45
140.23
133.48
125.92
125.87
123.78
123.60
120.53
119.14
114.69
110.87
103.24
77.45
76.81
31.70
31.18
22.75
14.10

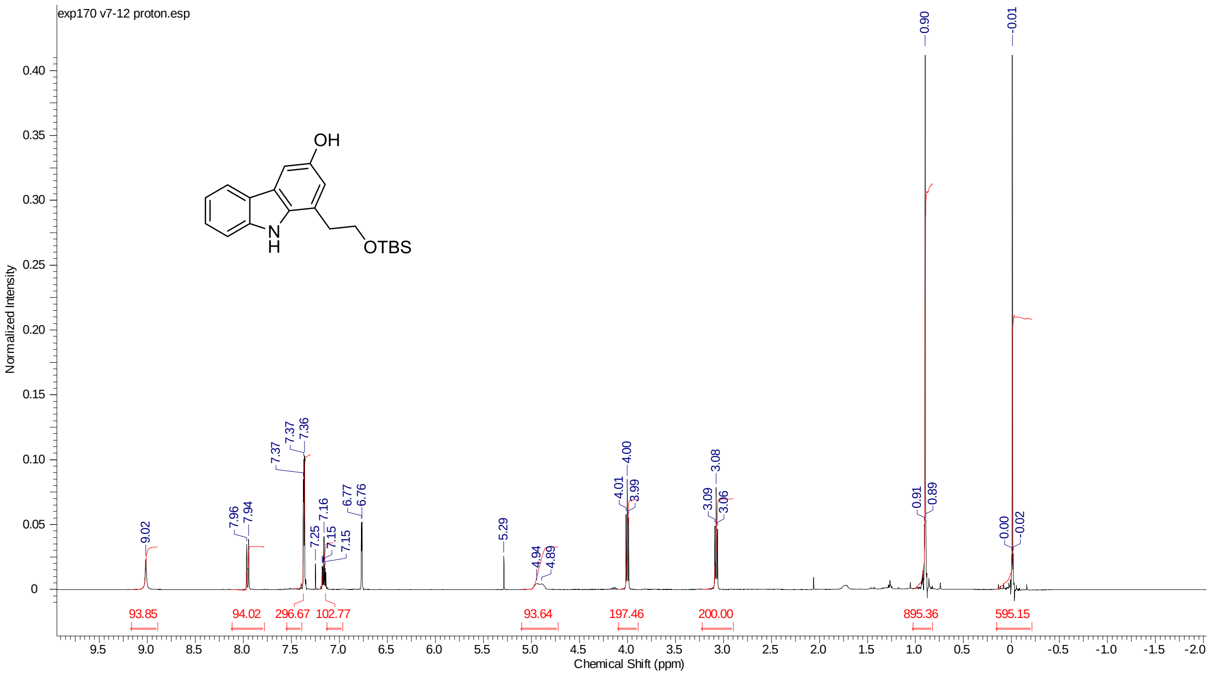
exp170 v7-12 proton.esp
OH
N
H
OTBS
Normalized Intensity
Chemical Shift (ppm)
9.02
7.96
7.94
7.37
7.37
7.36
7.25
7.16
7.15
7.15
6.77
6.76
5.29
4.94
4.89
4.01
4.00
3.99
3.09
3.08
3.06
0.91
0.90
0.89
0.00
-0.01
-0.02
93.85
94.02
296.67
102.77
93.64
197.46
200.00
895.36
595.15

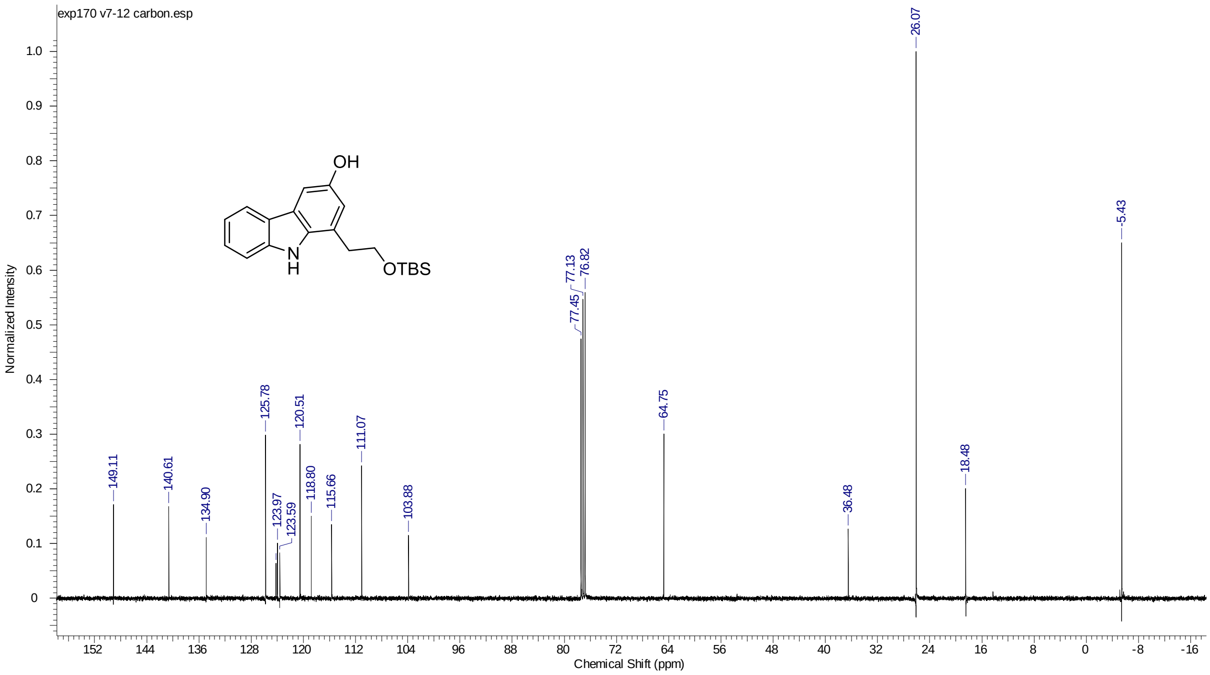
exp170 v7-12 carbon.esp
OH
N
H
OTBS
Normalized Intensity
Chemical Shift (ppm)
149.11
140.61
134.90
125.78
123.97
123.59
120.51
118.80
115.66
111.07
103.88
77.45
77.13
76.82
64.75
36.48
26.07
18.48
-5.43

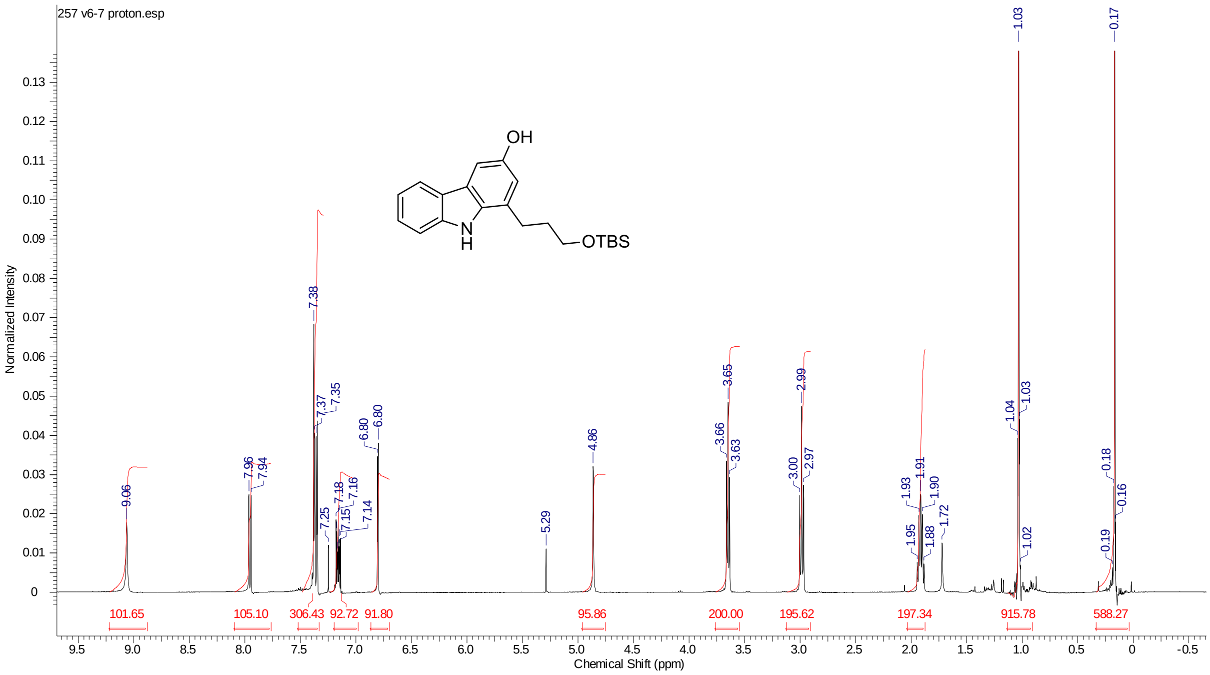

257 v6-7 proton.esp
OH
N
H
OTBS
Normalized Intensity
Chemical Shift (ppm)
9.06
7.96
7.94
7.38
7.37
7.35
7.25
7.18
7.16
7.15
7.14
6.80
6.80
5.29
4.86
3.66
3.65
3.63
3.00
2.99
2.97
1.95
1.93
1.91
1.90
1.88
1.72
1.04
1.03
1.03
1.02
0.19
0.18
0.17
0.16
101.65
105.10
306.43
92.72
91.80
95.86
200.00
195.62
197.34
915.78
588.27

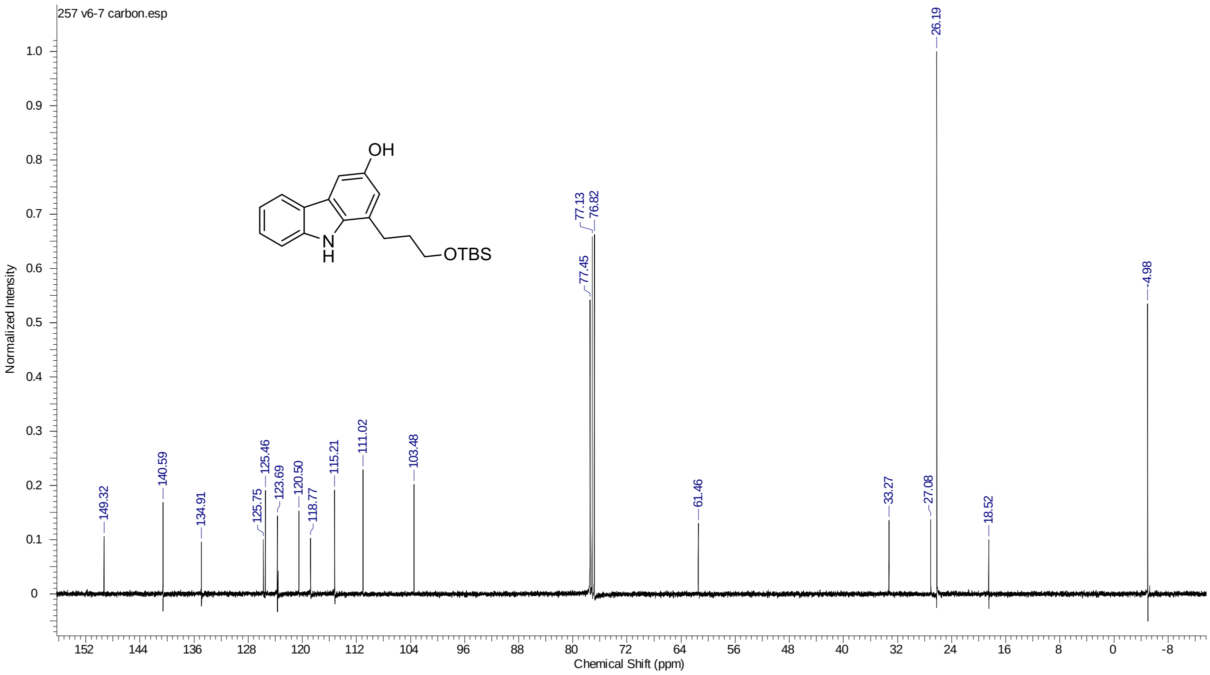

257 v6-7 carbon.esp
OH
N
H
OTBS
Normalized Intensity
Chemical Shift (ppm)
149.32
140.59
134.91
125.75
125.46
123.69
120.50
118.77
115.21
111.02
103.48
77.45
77.13
76.82
61.46
33.27
27.08
26.19
18.52
-4.98

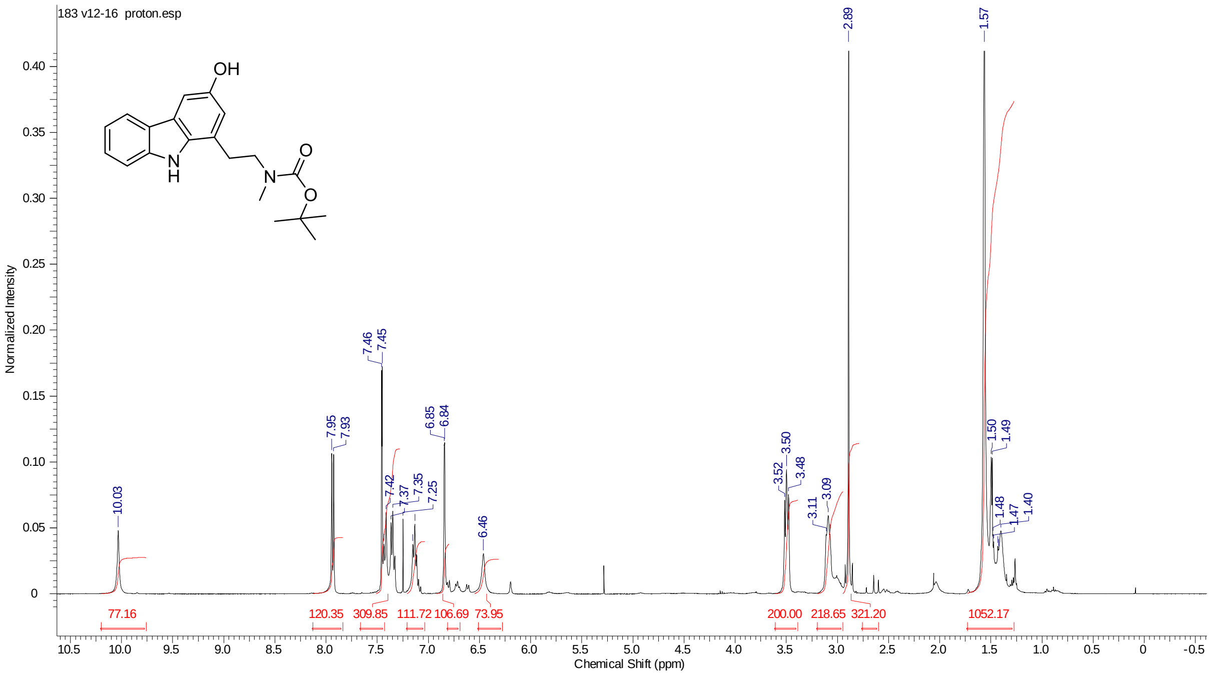
183 v12-16 proton.esp
OH
N
H
N
O
O
Normalized Intensity
Chemical Shift (ppm)
10.03
7.95
7.93
7.46
7.45
7.42
7.37
7.35
7.25
6.85
6.84
6.46
3.52
3.50
3.48
3.11
3.09
2.89
1.57
1.50
1.49
1.48
1.47
1.40
77.16
120.35
309.85
111.72
106.69
73.95
200.00
218.65
321.20
1052.17

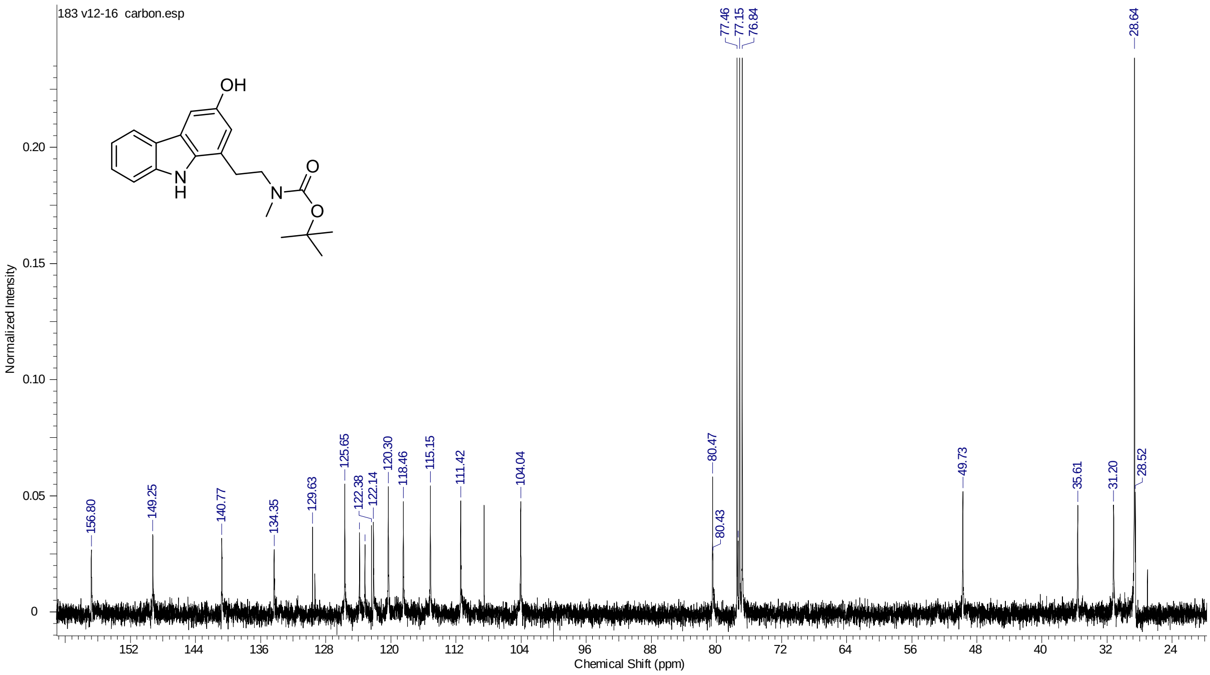
183 v12-16 carbon.esp
OH
N
H
N
O
O
Normalized Intensity
Chemical Shift (ppm)
156.80
149.25
140.77
134.35
129.63
125.65
122.38
122.14
120.30
118.46
115.15
111.42
104.04
80.47
80.43
77.46
77.15
76.84
49.73
35.61
31.20
28.64
28.52

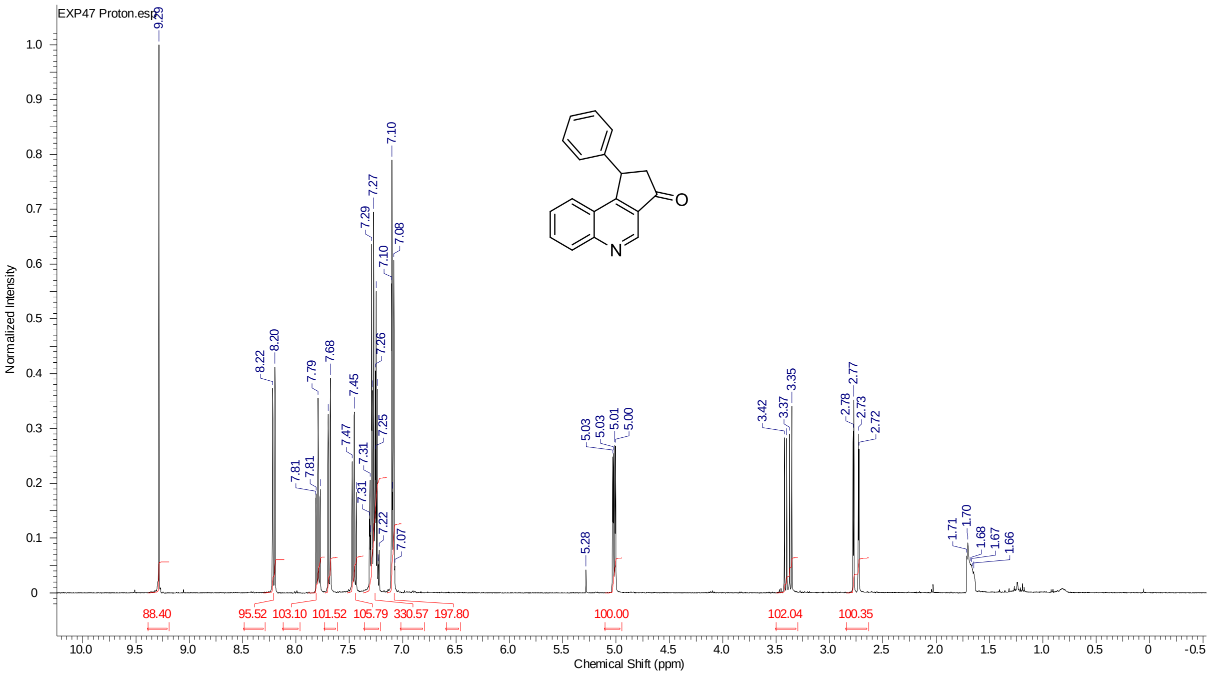
EXP47 Proton.esp
Normalized Intensity
Chemical Shift (ppm)
N
O

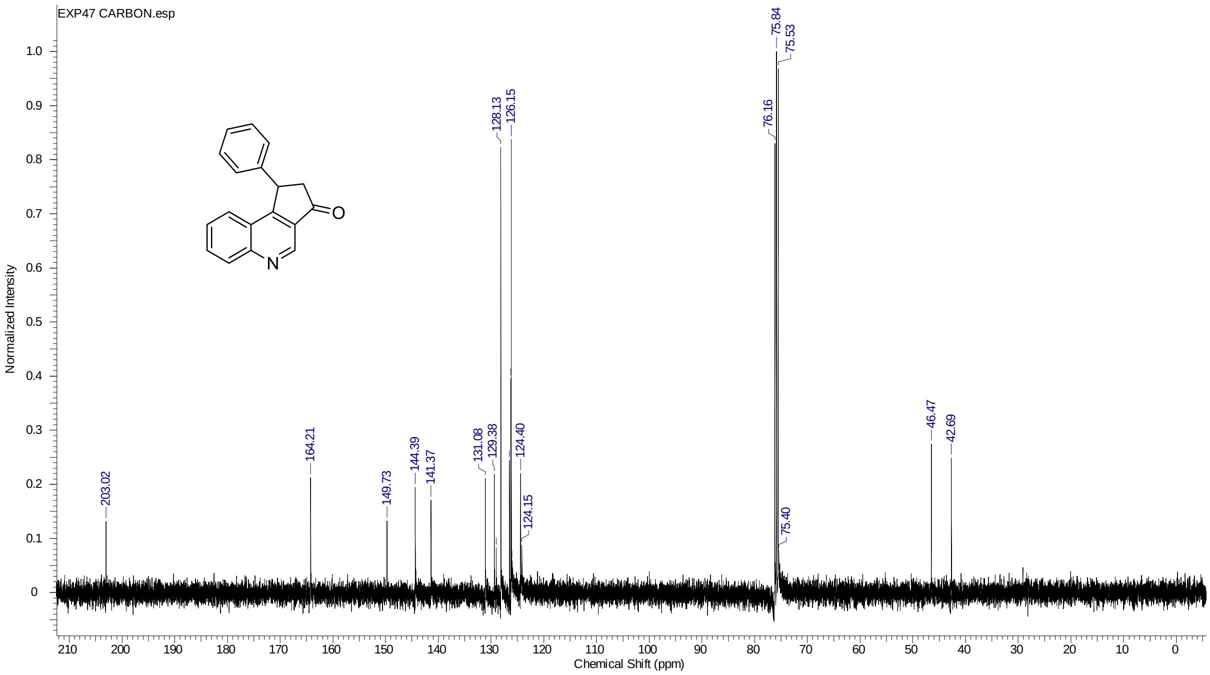
EXP47 CARBON.esp
Normalized Intensity
Chemical Shift (ppm)
N
O

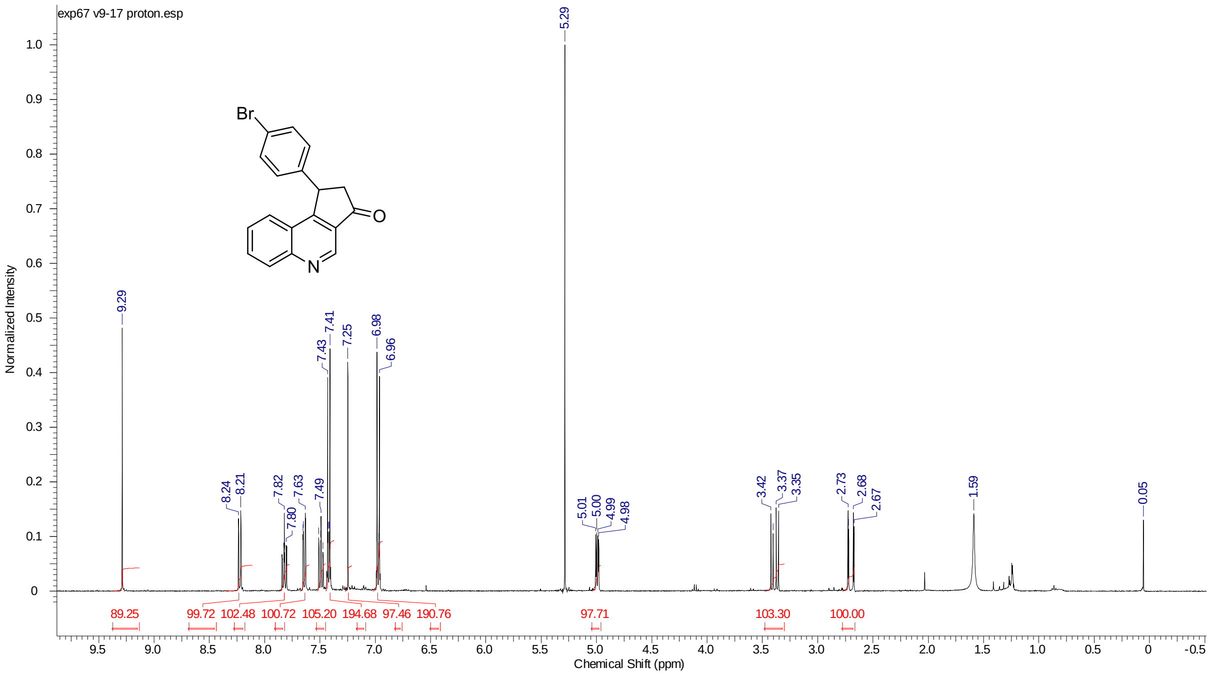
exp67 v9-17 proton.esp
Normalized Intensity
Chemical Shift (ppm)
Br
O
N
9.29
8.24
8.21
7.82
7.80
7.63
7.49
7.43
7.41
7.25
6.98
6.96
5.29
5.01
5.00
4.99
4.98
3.42
3.37
3.35
2.73
2.68
2.67
1.59
0.05
89.25
99.72
102.48
100.72
105.20
194.68
97.46
190.76
97.71
103.30
100.00

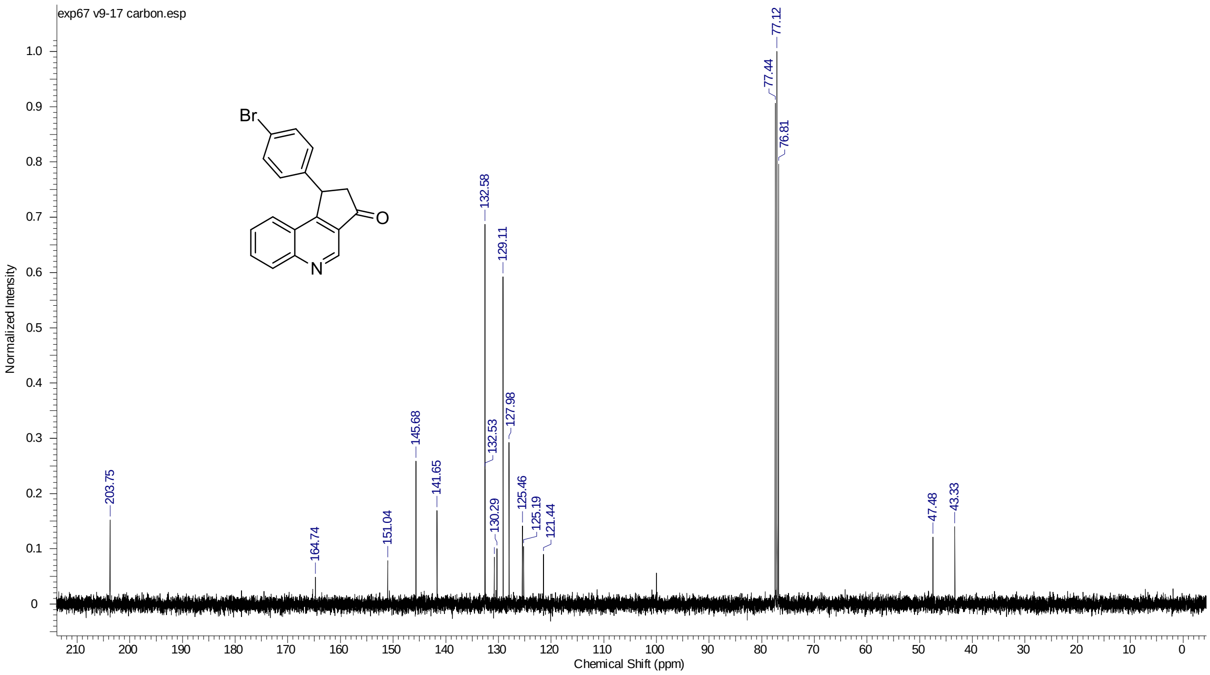
exp67 v9-17 carbon.esp
Normalized Intensity
Chemical Shift (ppm)
Br
O
N
203.75
164.74
151.04
145.68
141.65
132.58
132.53
130.29
129.11
127.98
125.46
125.19
121.44
77.44
77.12
76.81
47.48
43.33

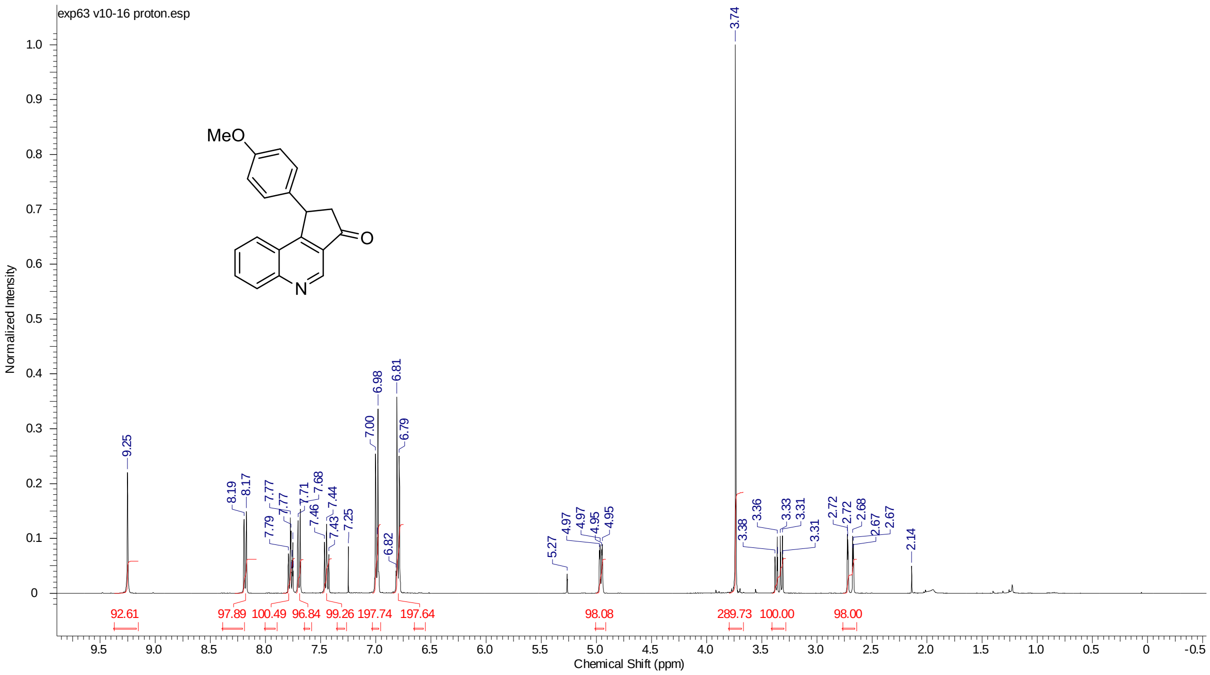
exp63 v10-16 proton.esp
MeO
O
N
Normalized Intensity
Chemical Shift (ppm)
9.25
8.19
8.17
7.79
7.77
7.77
7.71
7.68
7.46
7.44
7.43
7.25
7.00
6.98
6.82
6.81
6.79
5.27
4.97
4.97
4.95
4.95
3.74
3.38
3.36
3.33
3.31
3.31
2.72
2.72
2.68
2.67
2.67
2.14
92.61
97.89
100.49
96.84
99.26
197.74
197.64
98.08
289.73
100.00
98.00

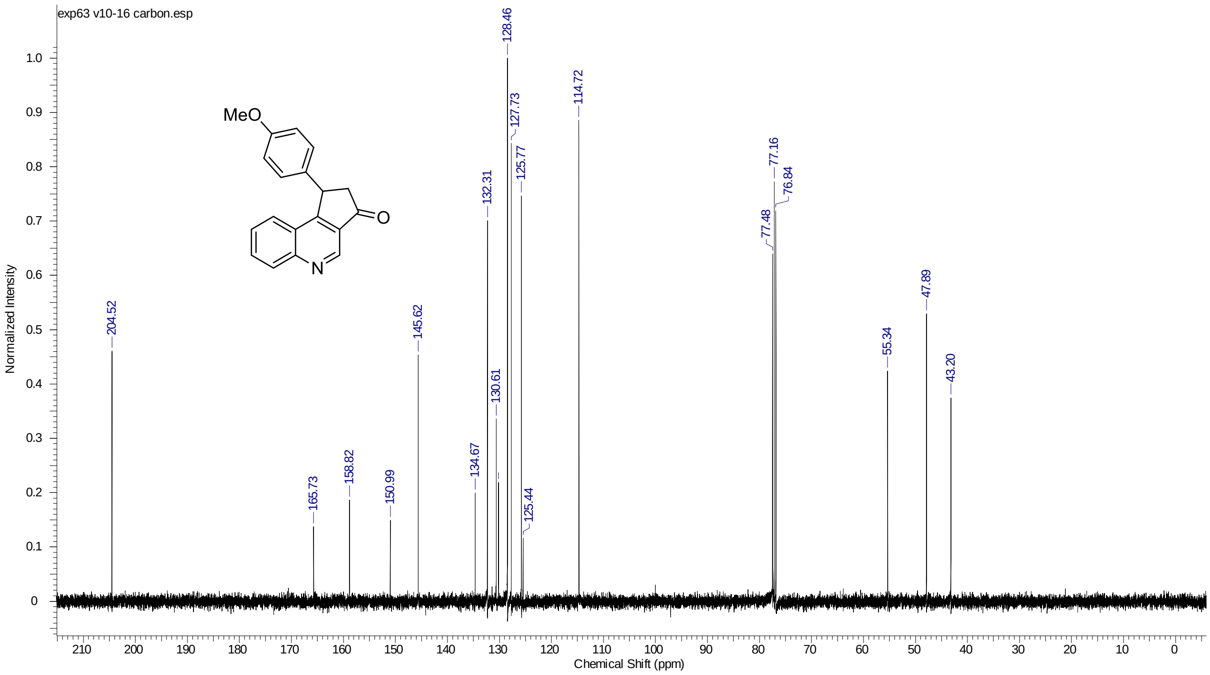
exp63 v10-16 carbon.esp
MeO
O
N
Normalized Intensity
Chemical Shift (ppm)
204.52
165.73
158.82
150.99
145.62
134.67
132.31
130.61
128.46
127.73
125.77
125.44
114.72
77.48
77.16
76.84
55.34
47.89
43.20

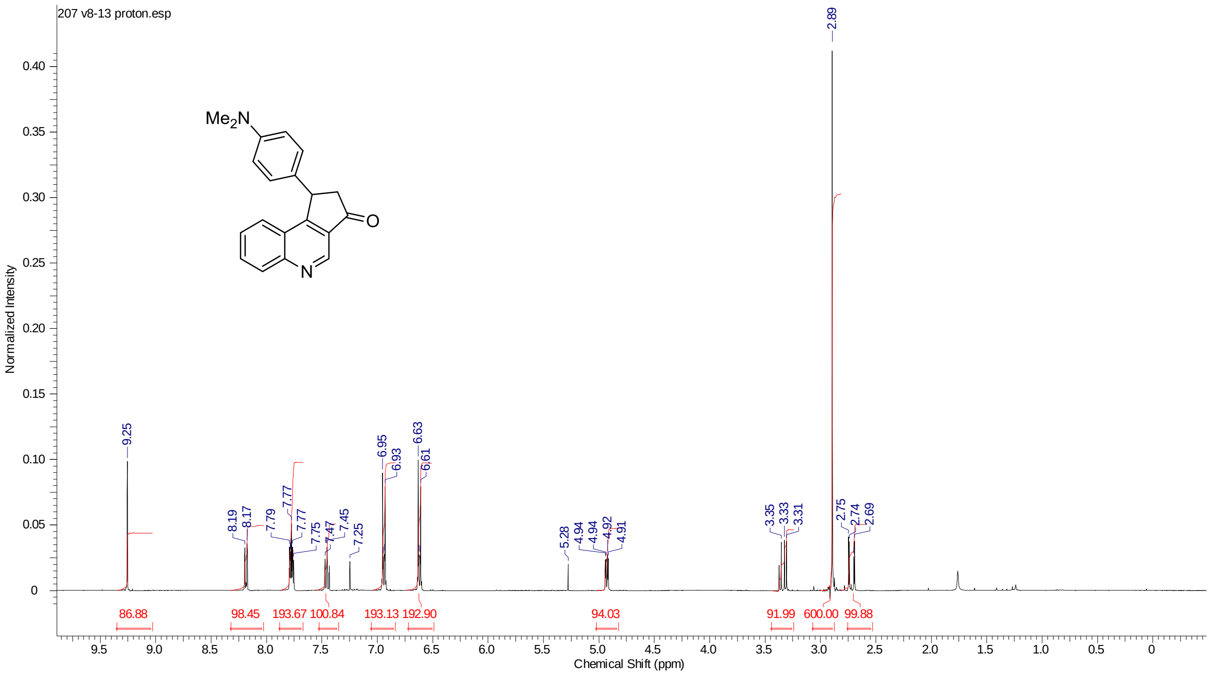
207 v8-13 proton.esp
Me2N
N
O
Normalized Intensity
Chemical Shift (ppm)
9.25
8.19
8.17
7.79
7.77
7.77
7.75
7.47
7.45
7.25
6.95
6.93
6.63
6.61
5.28
4.94
4.94
4.92
4.91
3.35
3.33
3.31
2.89
2.75
2.74
2.69
86.88
98.45
193.67
100.84
193.13
192.90
94.03
91.99
600.00
99.88

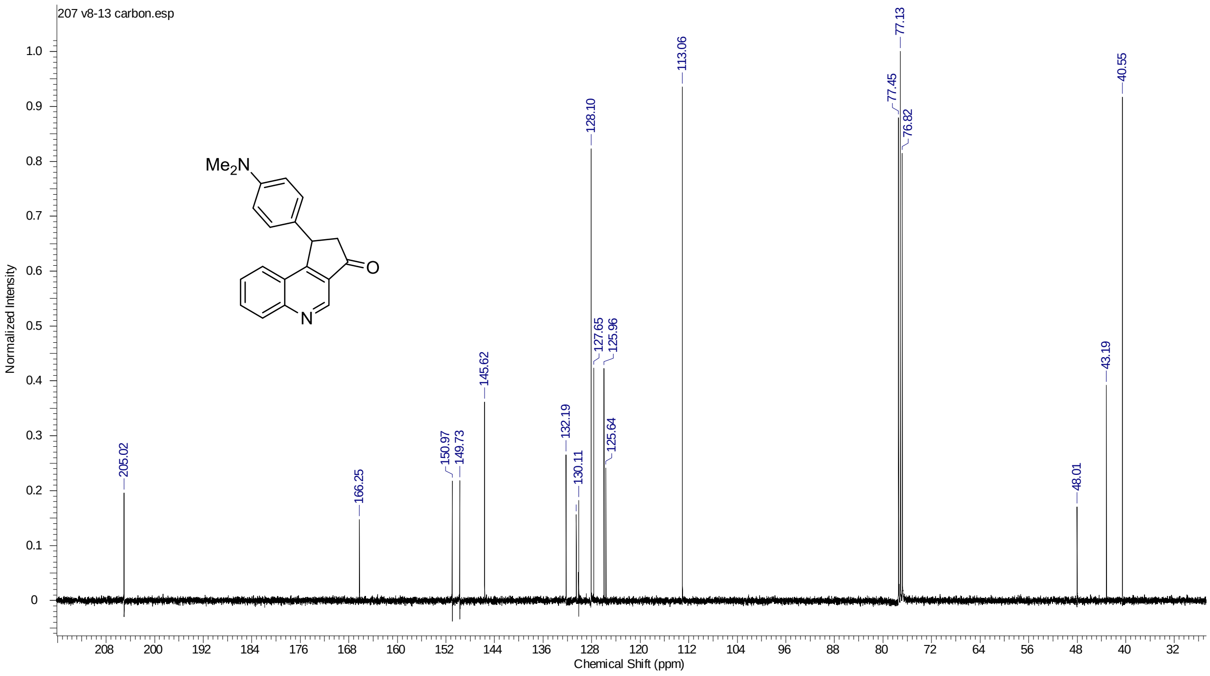
207 v8-13 carbon.esp
Me2N
N
O
Normalized Intensity
Chemical Shift (ppm)
205.02
166.25
150.97
149.73
145.62
132.19
130.11
128.10
127.65
125.96
125.64
113.06
77.45
77.13
76.82
48.01
43.19
40.55

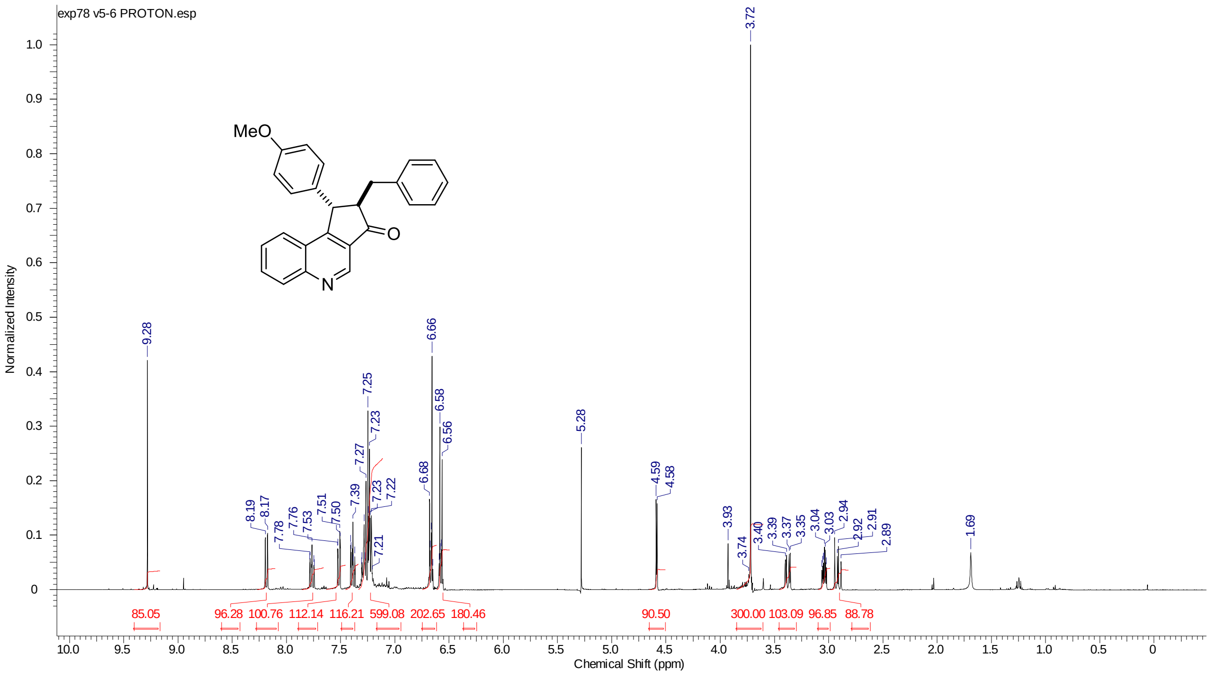
exp78 v5-6 PROTON.esp
MeO
N
O
9.28
8.19
8.17
7.78
7.76
7.53
7.51
7.50
7.39
7.27
7.25
7.23
7.23
7.22
7.21
6.68
6.66
6.58
6.56
5.28
4.59
4.58
3.93
3.74
3.72
3.40
3.39
3.37
3.35
3.04
3.03
2.94
2.92
2.91
2.89
1.69
85.05
96.28
100.76
112.14
116.21
599.08
202.65
180.46
90.50
300.00
103.09
96.85
88.78
Normalized Intensity
Chemical Shift (ppm)

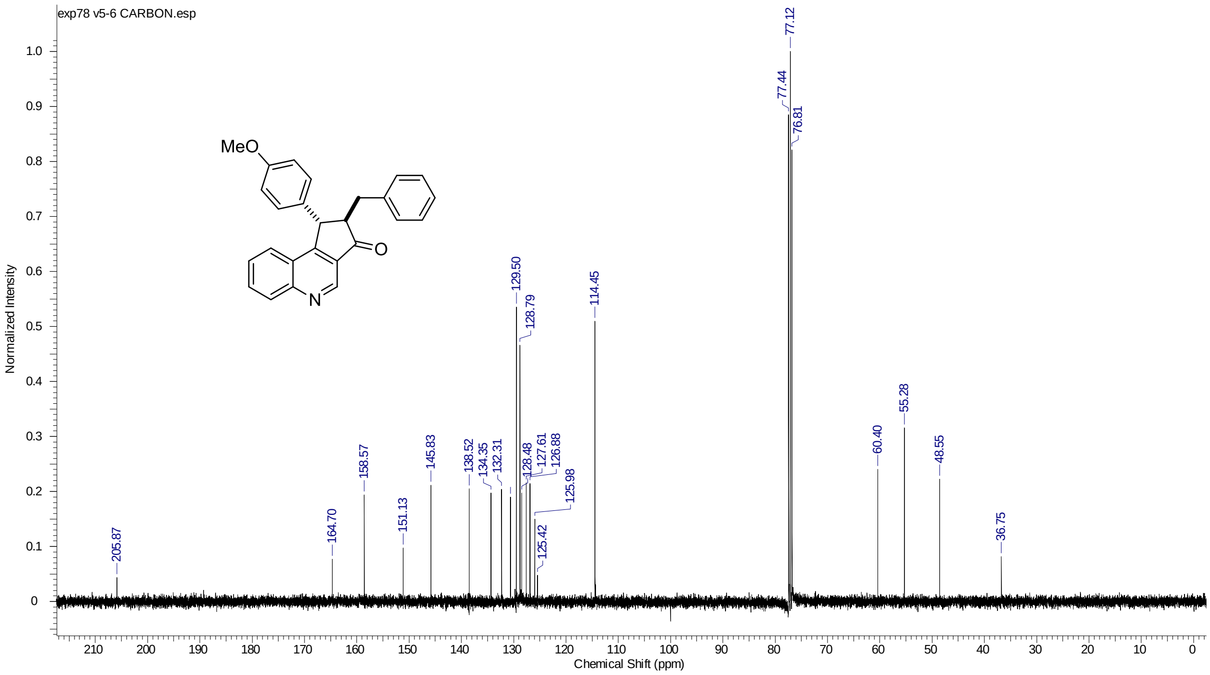
exp78 v5-6 CARBON.esp
MeO
N
O
205.87
164.70
158.57
151.13
145.83
138.52
134.35
132.31
129.50
128.79
128.48
127.61
126.88
125.98
125.42
114.45
77.44
77.12
76.81
60.40
55.28
48.55
36.75
Normalized Intensity
Chemical Shift (ppm)

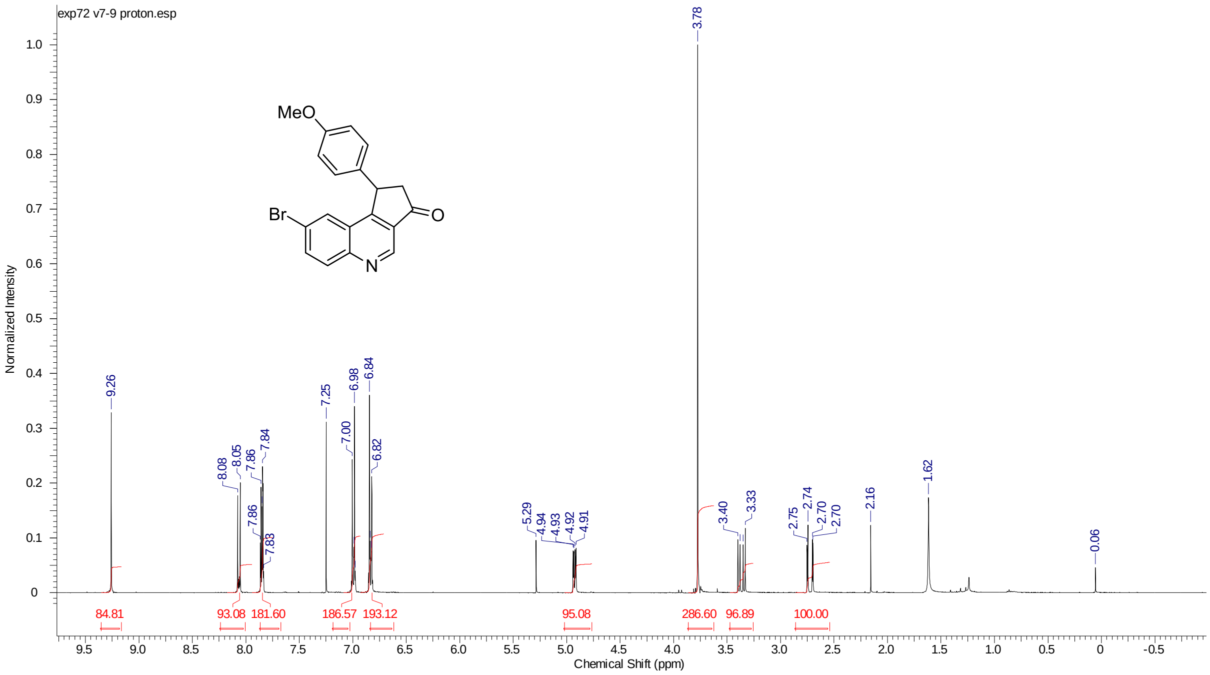
exp72 v7-9 proton.esp
Normalized Intensity
Chemical Shift (ppm)
MeO
Br
N
O
9.26
8.08
8.05
7.86
7.84
7.86
7.83
7.25
7.00
6.98
6.84
6.82
5.29
4.94
4.93
4.92
4.91
3.78
3.40
3.33
2.75
2.74
2.70
2.70
2.16
1.62
0.06
84.81
93.08
181.60
186.57
193.12
95.08
286.60
96.89
100.00

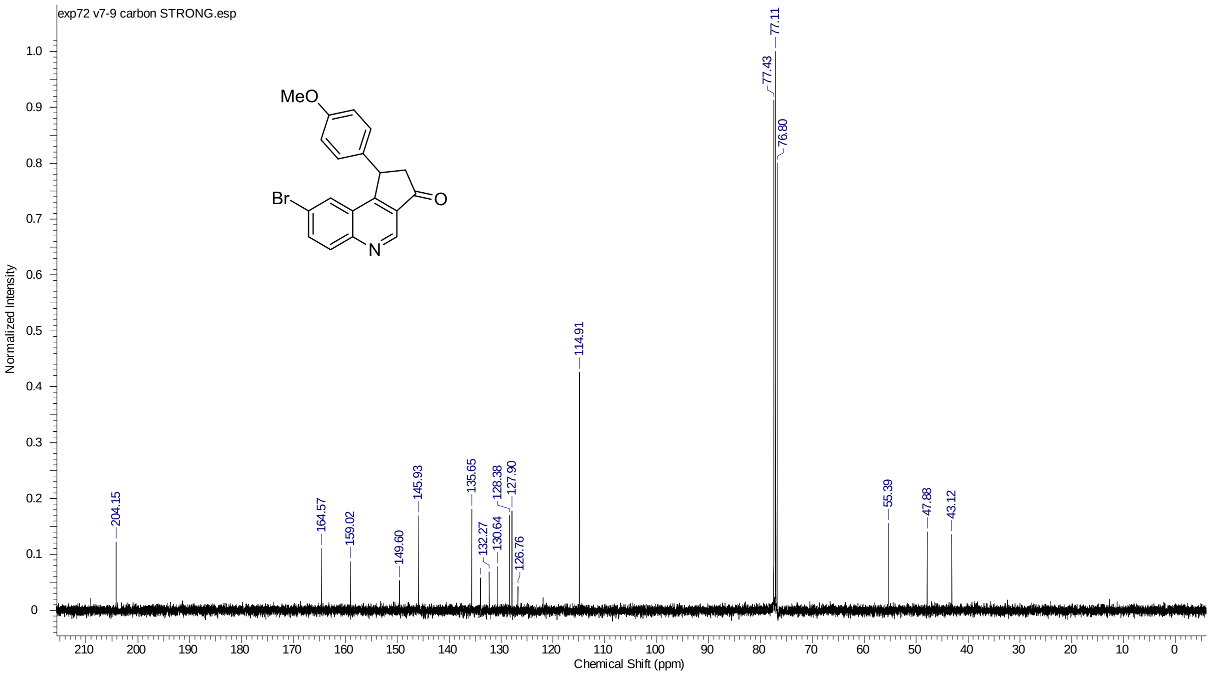
exp72 v7-9 carbon STRONG.esp
Normalized Intensity
Chemical Shift (ppm)
MeO
Br
N
O
204.15
164.57
159.02
149.60
145.93
135.65
132.27
130.64
128.38
127.90
126.76
114.91
77.43
77.11
76.80
55.39
47.88
43.12

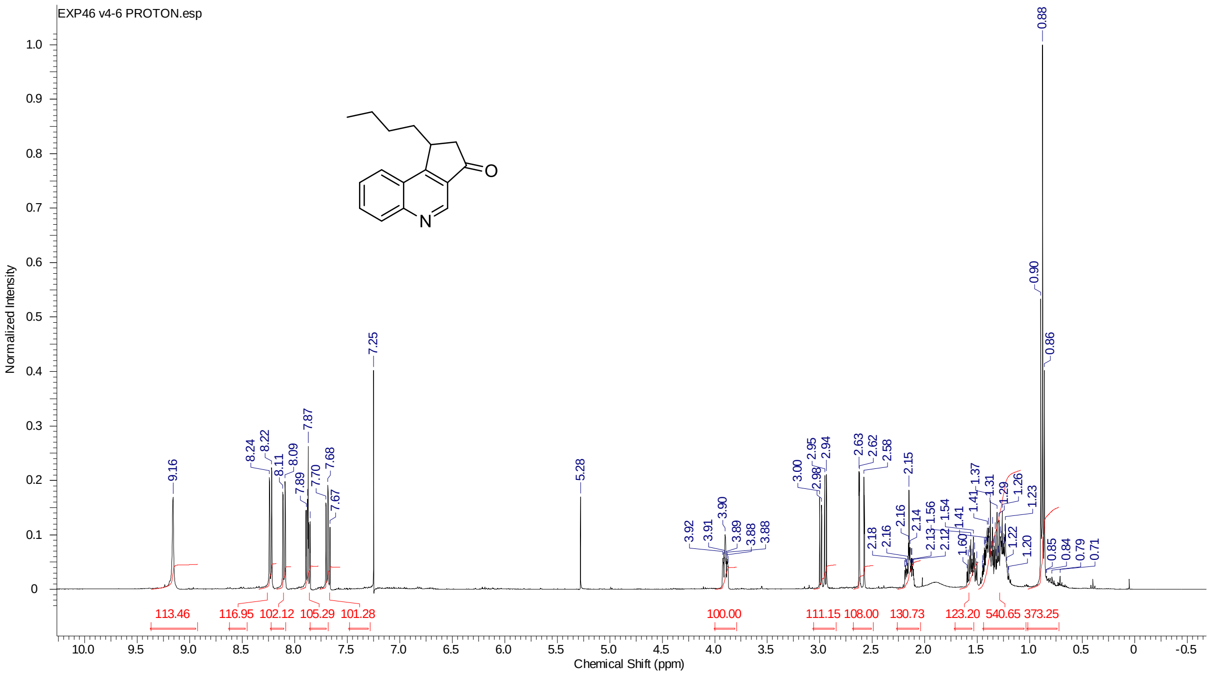
EXP46 v4-6 PROTON.esp
Normalized Intensity
Chemical Shift (ppm)
N
O
9.16
8.24
8.22
8.11
8.09
7.89
7.87
7.70
7.68
7.67
7.25
5.28
3.92
3.91
3.90
3.89
3.88
3.88
3.00
2.98
2.95
2.94
2.63
2.62
2.58
2.18
2.16
2.16
2.15
2.14
2.13
2.12
1.60
1.56
1.54
1.41
1.41
1.37
1.31
1.29
1.26
1.23
1.22
1.20
0.90
0.88
0.86
0.85
0.84
0.79
0.71
113.46
116.95
102.12
105.29
101.28
100.00
111.15
108.00
130.73
123.20
540.65
373.25

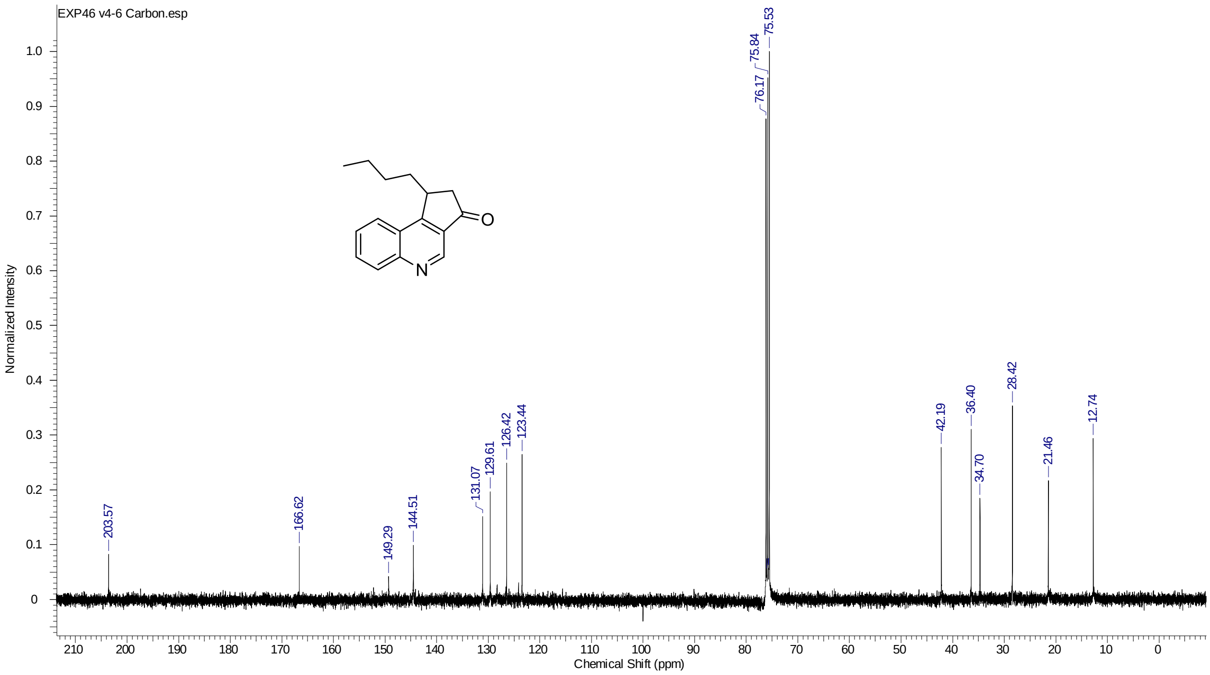
EXP46 v4-6 Carbon.esp
Normalized Intensity
Chemical Shift (ppm)
N
O
203.57
166.62
149.29
144.51
131.07
129.61
126.42
123.44
76.17
75.84
75.53
42.19
36.40
34.70
28.42
21.46
12.74

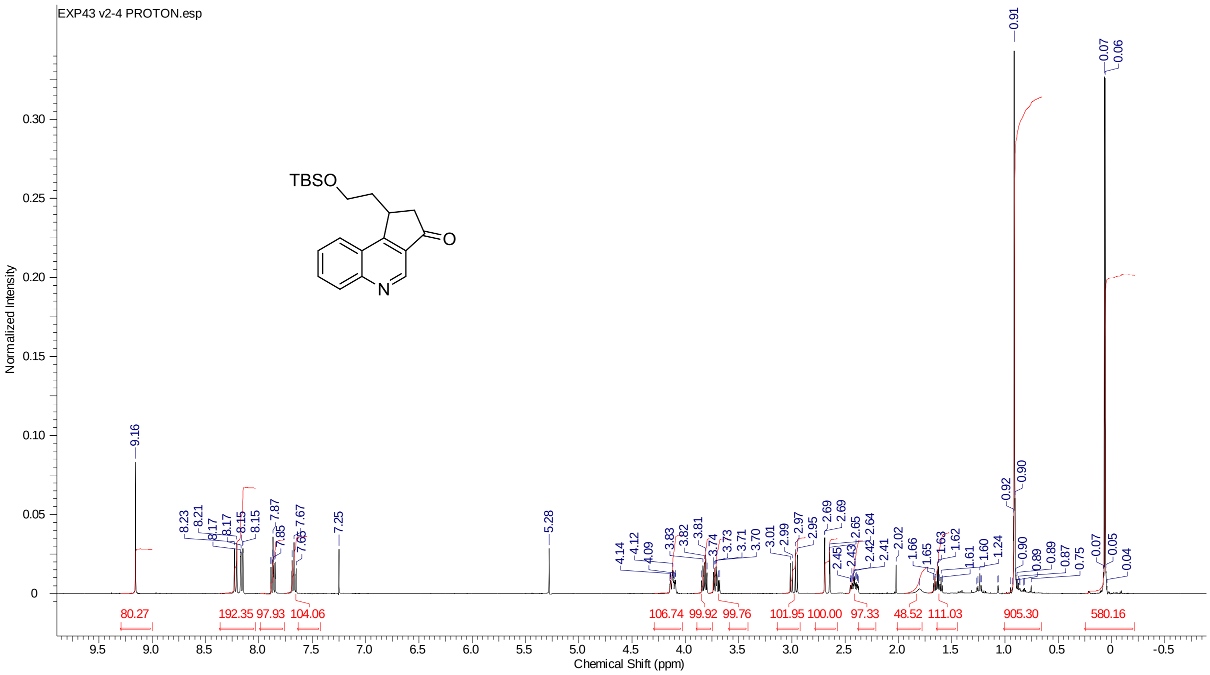
EXP43 v2-4 PROTON.esp
TBSO
O
N
Normalized Intensity
Chemical Shift (ppm)

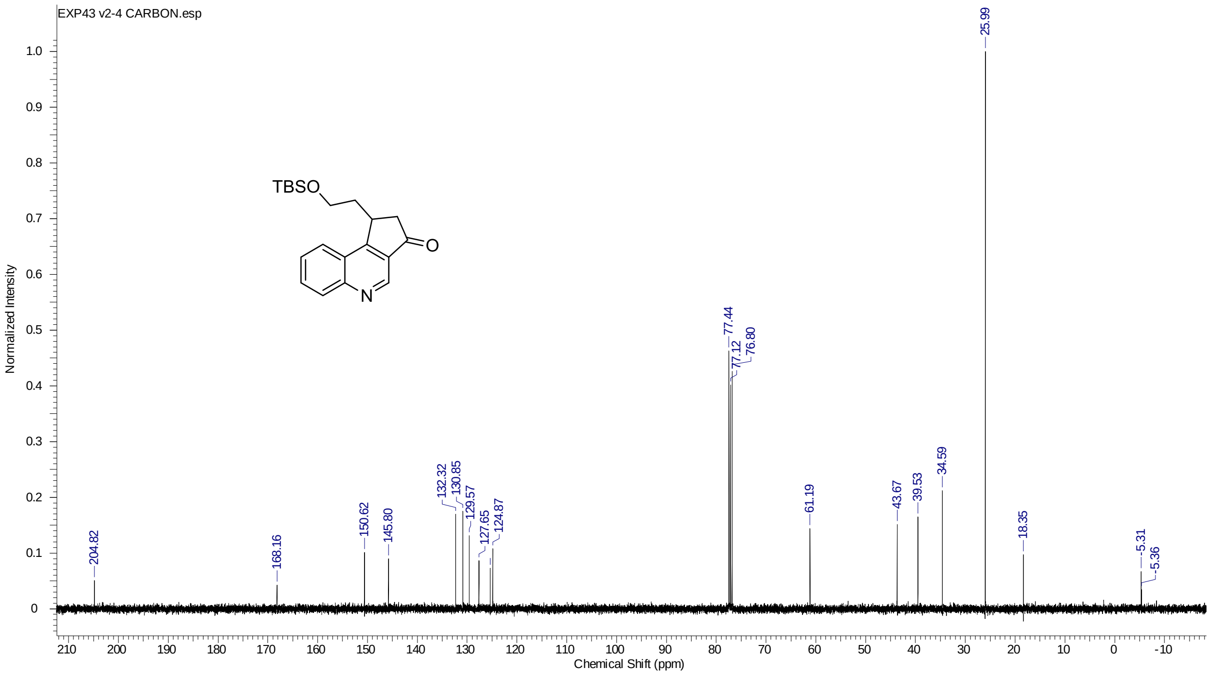
EXP43 v2-4 CARBON.esp
TBSO
O
N
Normalized Intensity
Chemical Shift (ppm)

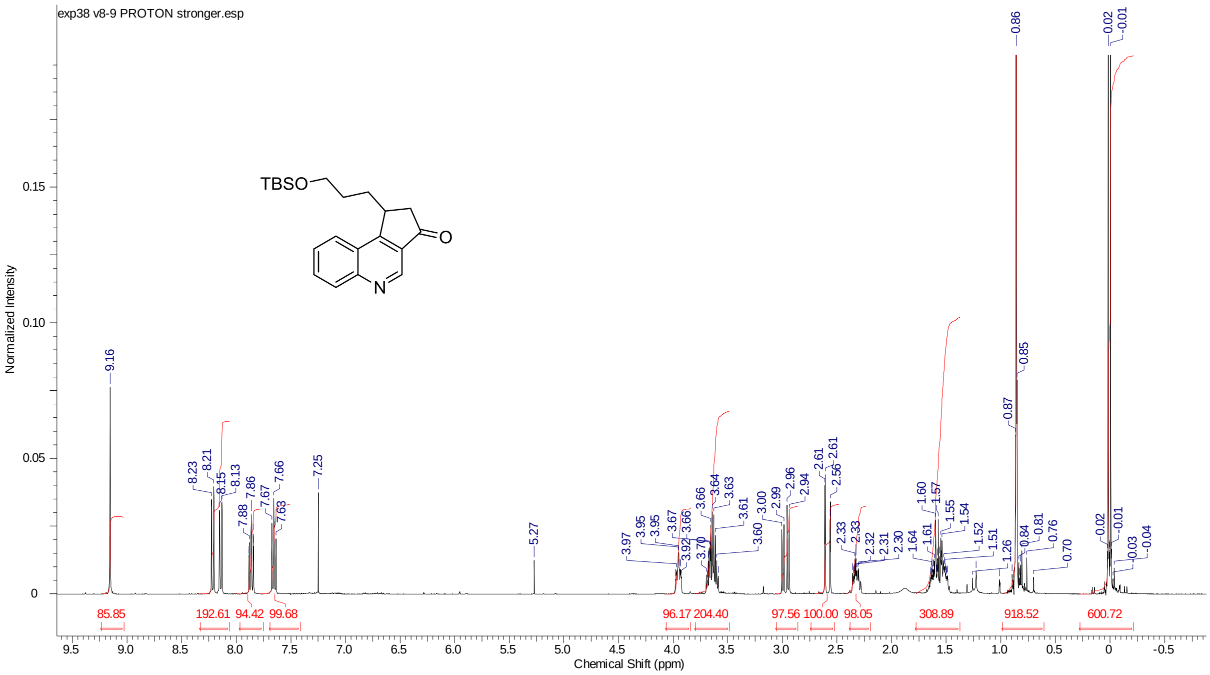
exp38 v8-9 PROTON stronger.esp
TBSO
O
N
Normalized Intensity
Chemical Shift (ppm)
9.16
8.23
8.21
8.15
8.13
7.88
7.86
7.67
7.66
7.63
7.25
5.27
3.97
3.95
3.95
3.92
3.70
3.67
3.66
3.66
3.64
3.63
3.61
3.60
3.00
2.99
2.96
2.94
2.61
2.61
2.56
2.33
2.33
2.32
2.31
2.30
1.64
1.61
1.60
1.57
1.55
1.54
1.52
1.51
1.26
0.87
0.86
0.85
0.84
0.81
0.76
0.70
0.02
-0.01
0.02
-0.01
-0.03
-0.04
85.85
192.61
94.42
99.68
96.17
204.40
97.56
100.00
98.05
308.89
918.52
600.72

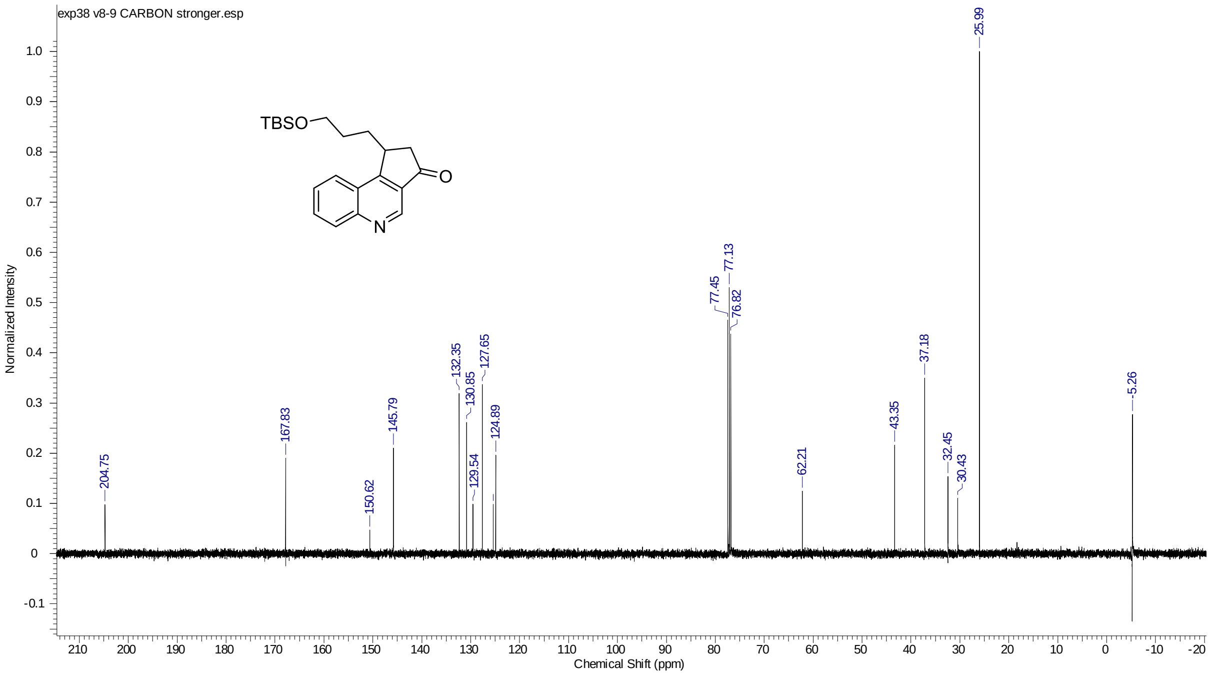
exp38 v8-9 CARBON stronger.esp
TBSO
O
N
Normalized Intensity
Chemical Shift (ppm)
204.75
167.83
150.62
145.79
132.35
130.85
129.54
127.65
124.89
77.45
77.13
76.82
62.21
43.35
37.18
32.45
30.43
25.99
-5.26

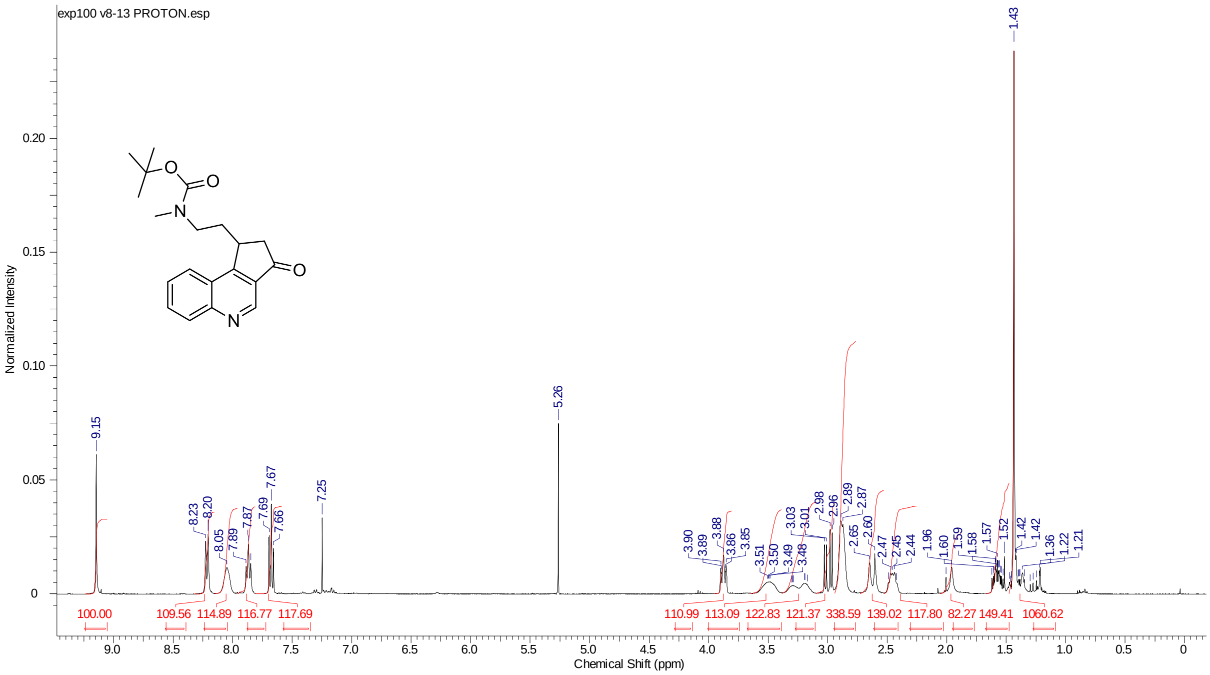
exp100 v8-13 PROTON.esp
Normalized Intensity
Chemical Shift (ppm)
9.15
8.23
8.20
8.05
7.89
7.87
7.69
7.67
7.66
7.25
5.26
3.90
3.89
3.88
3.86
3.85
3.51
3.50
3.49
3.48
3.03
3.01
2.98
2.96
2.89
2.87
2.65
2.60
2.47
2.45
2.44
1.96
1.60
1.59
1.58
1.57
1.52
1.43
1.42
1.42
1.36
1.22
1.21
100.00
109.56
114.89
116.77
117.69
110.99
113.09
122.83
121.37
338.59
139.02
117.80
82.27
149.41
1060.62
O
N
O
O
N
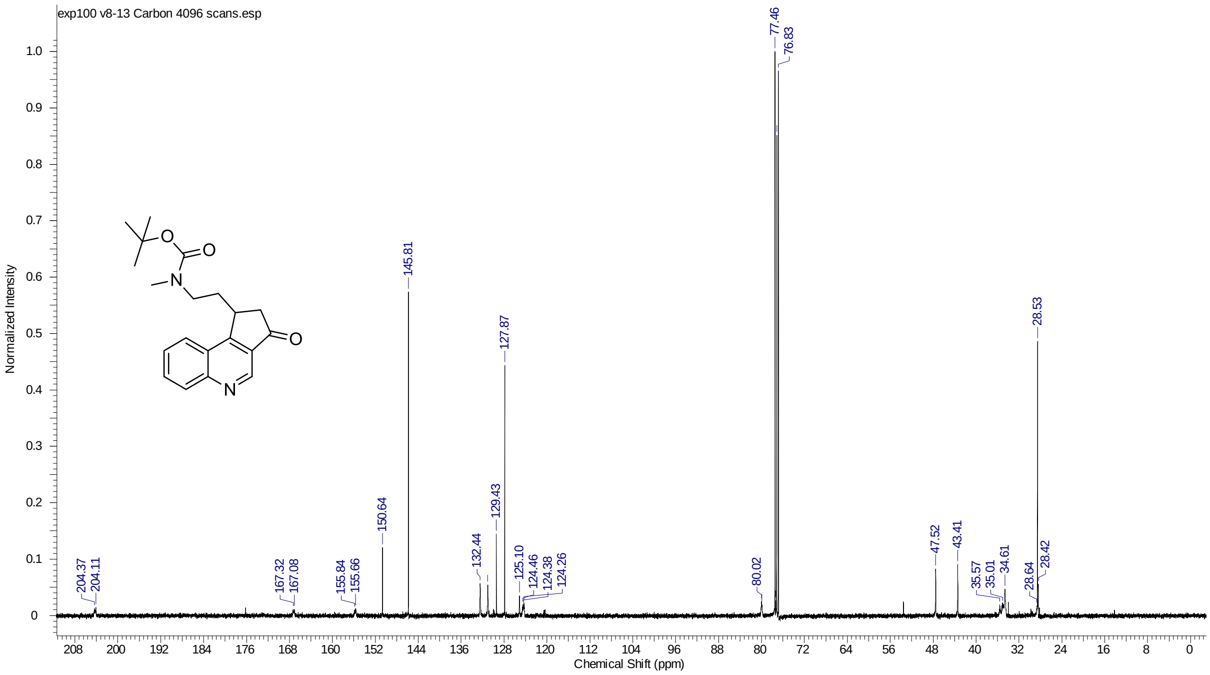
exp100 v8-13 Carbon 4096 scans.esp
Normalized Intensity
Chemical Shift (ppm)
204.37
204.11
167.32
167.08
155.84
155.66
150.64
145.81
132.44
129.43
127.87
125.10
124.46
124.38
124.26
80.02
77.46
76.83
47.52
43.41
35.57
35.01
34.61
28.64
28.53
28.42
O
N
O
O
N

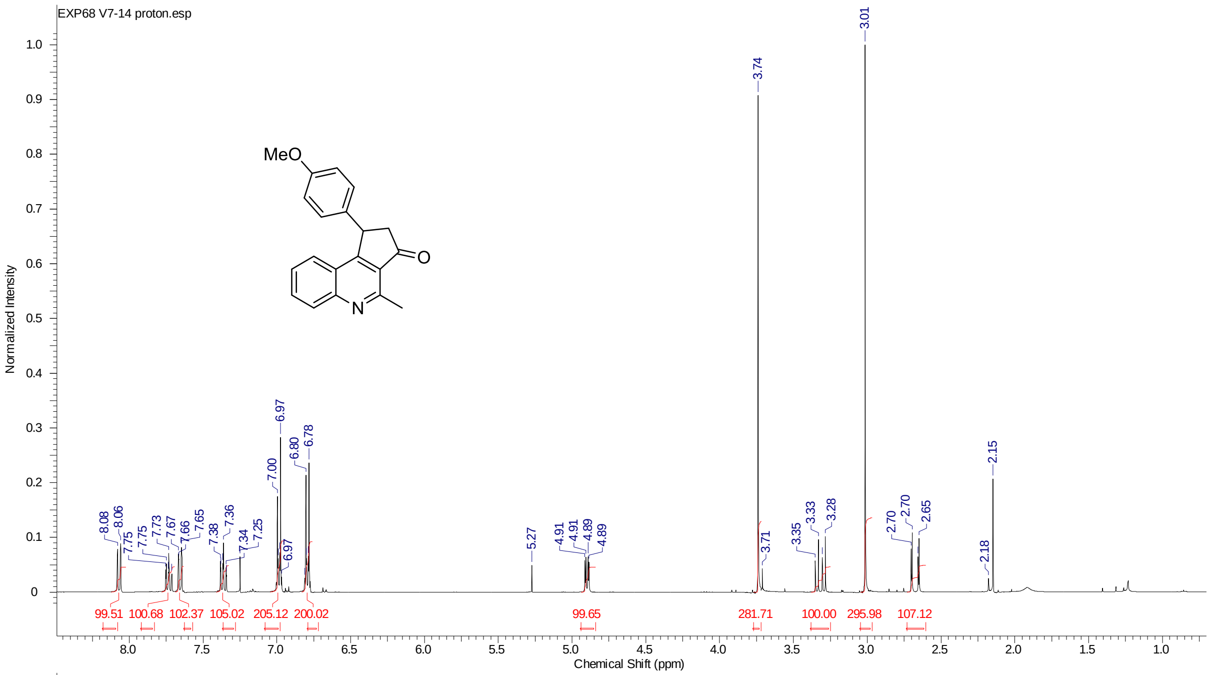
EXP68 V7-14 proton.esp
MeO
O
N
Normalized Intensity
Chemical Shift (ppm)
8.08 8.06 7.75 7.75 7.73 7.67 7.66 7.65 7.38 7.36 7.34 7.25 7.00 6.97 6.97 6.80 6.78 5.27 4.91 4.91 4.89 4.89 3.74 3.71 3.35 3.33 3.28 3.01 2.70 2.70 2.65 2.18 2.15
99.51 100.68 102.37 105.02 205.12 200.02 99.65 281.71 100.00 295.98 107.12

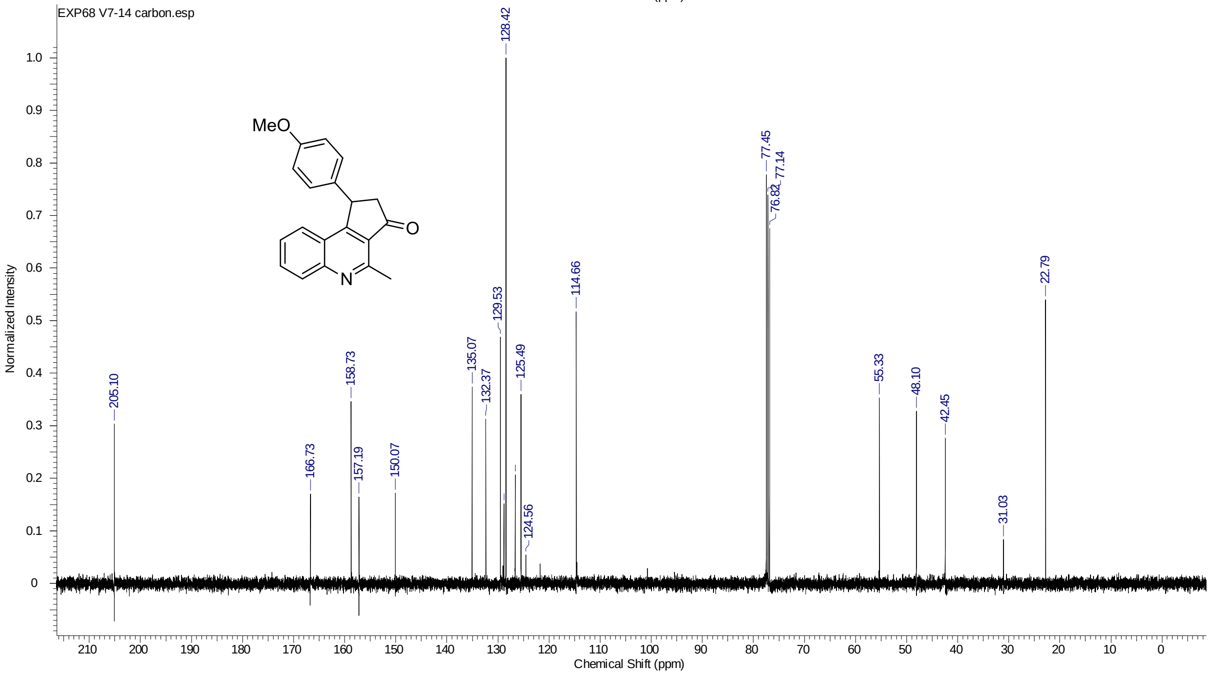
EXP68 V7-14 carbon.esp
MeO
O
N
Normalized Intensity
Chemical Shift (ppm)
205.10 166.73 158.73 157.19 150.07 135.07 132.37 129.53 128.42 125.49 124.56 114.66 77.45 77.14 76.82 55.33 48.10 42.45 31.03 22.79

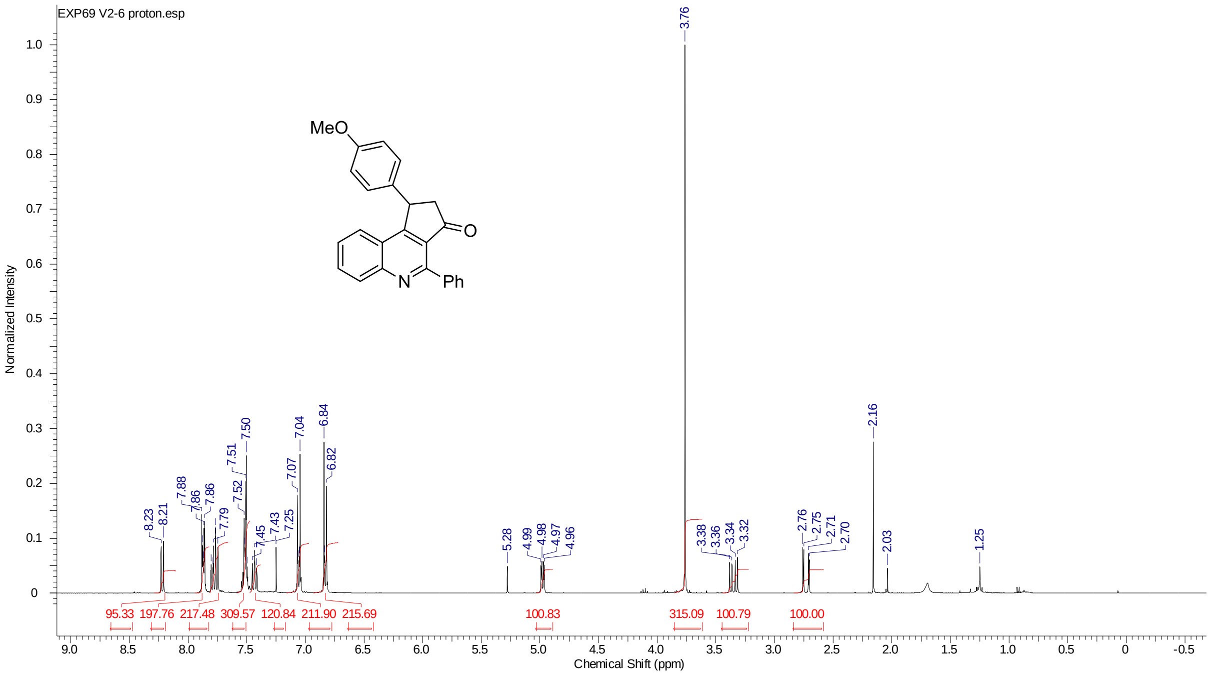
EXP69 V2-6 proton.esp
MeO
O
N
Ph
Normalized Intensity
Chemical Shift (ppm)
8.23
8.21
7.88
7.86
7.86
7.79
7.52
7.51
7.50
7.45
7.43
7.25
7.07
7.04
6.84
6.82
5.28
4.99
4.98
4.97
4.96
3.76
3.38
3.36
3.34
3.32
2.76
2.75
2.71
2.70
2.16
2.03
1.25
95.33
197.76
217.48
309.57
120.84
211.90
215.69
100.83
315.09
100.79
100.00

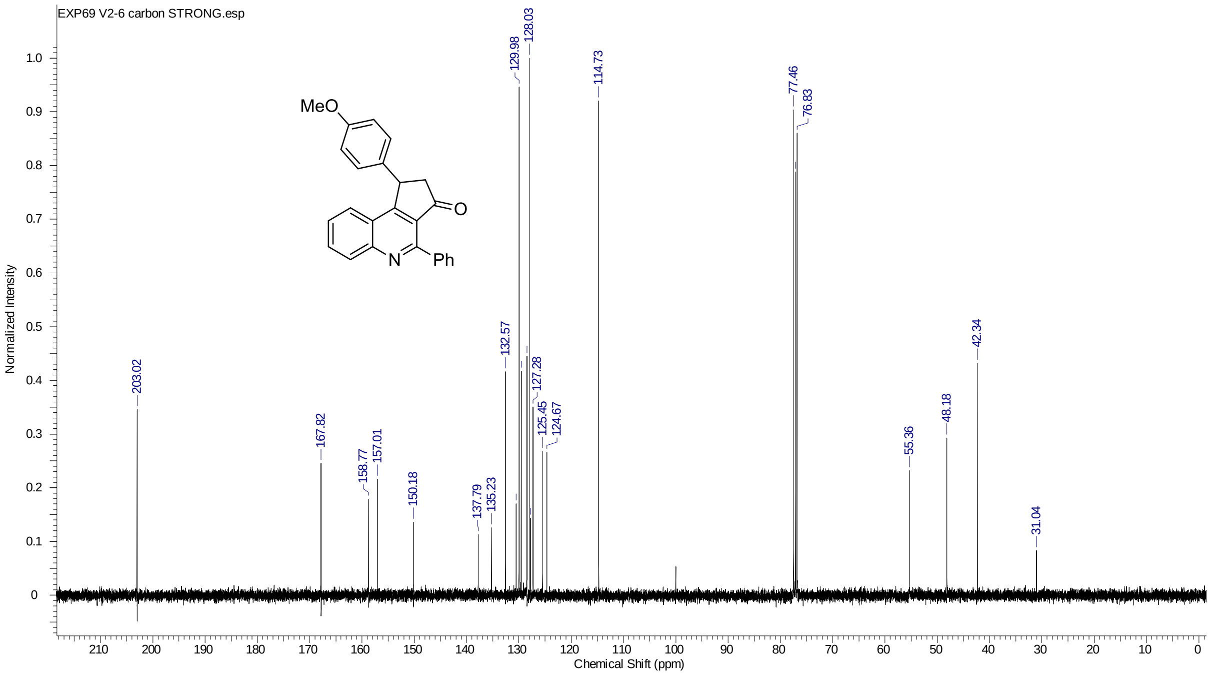
EXP69 V2-6 carbon STRONG.esp
MeO
O
N
Ph
Normalized Intensity
Chemical Shift (ppm)
203.02
167.82
158.77
157.01
150.18
137.79
135.23
132.57
129.98
128.03
127.28
125.45
124.67
114.73
77.46
76.83
55.36
48.18
42.34
31.04

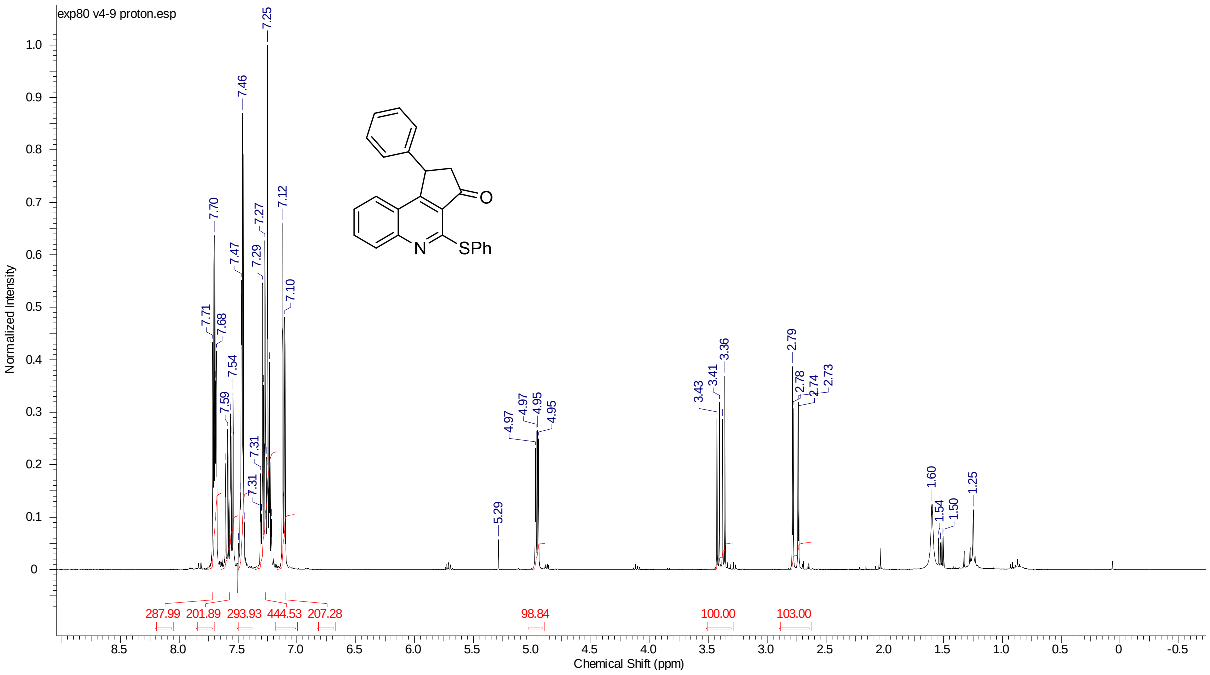
exp80 v4-9 proton.esp
Normalized Intensity
Chemical Shift (ppm)
7.25
7.46
7.70
7.27
7.12
7.47
7.29
7.10
7.71
7.68
7.54
7.59
7.31
7.31
5.29
4.97
4.97
4.95
4.95
3.43
3.41
3.36
2.79
2.78
2.74
2.73
1.60
1.54
1.50
1.25
287.99
201.89
293.93
444.53
207.28
98.84
100.00
103.00
N
SPh
O

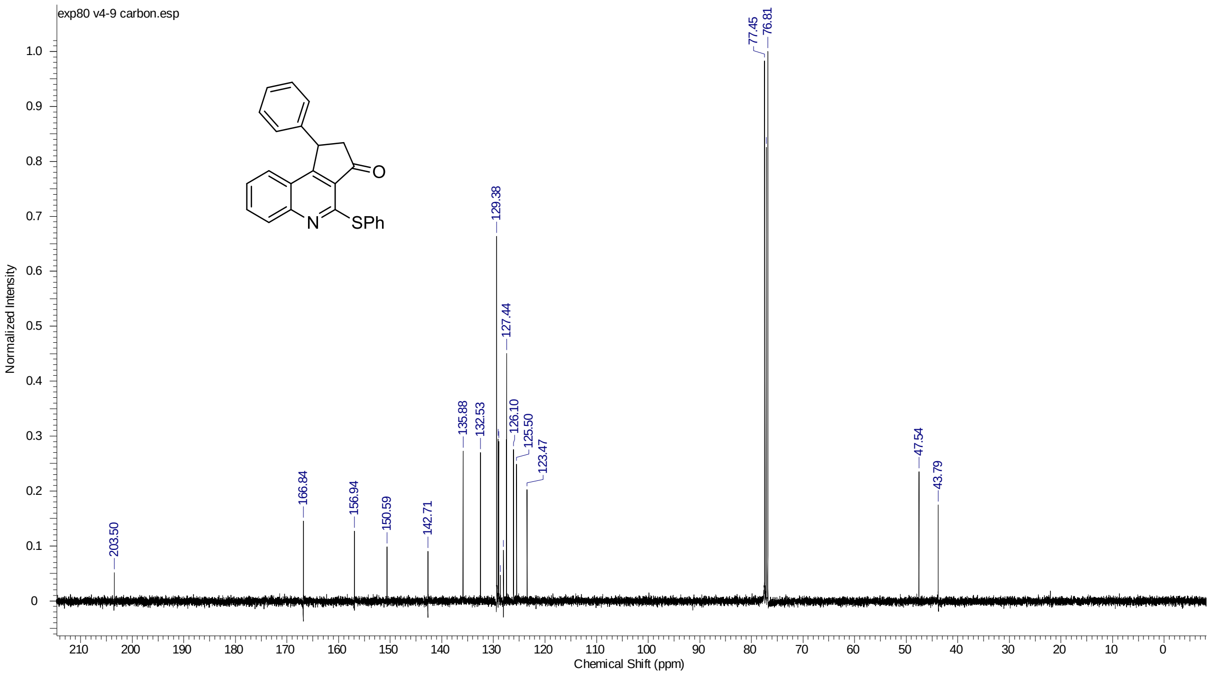
exp80 v4-9 carbon.esp
Normalized Intensity
Chemical Shift (ppm)
77.45
76.81
129.38
127.44
135.88
132.53
126.10
125.50
123.47
47.54
43.79
203.50
166.84
156.94
150.59
142.71
N
SPh
O

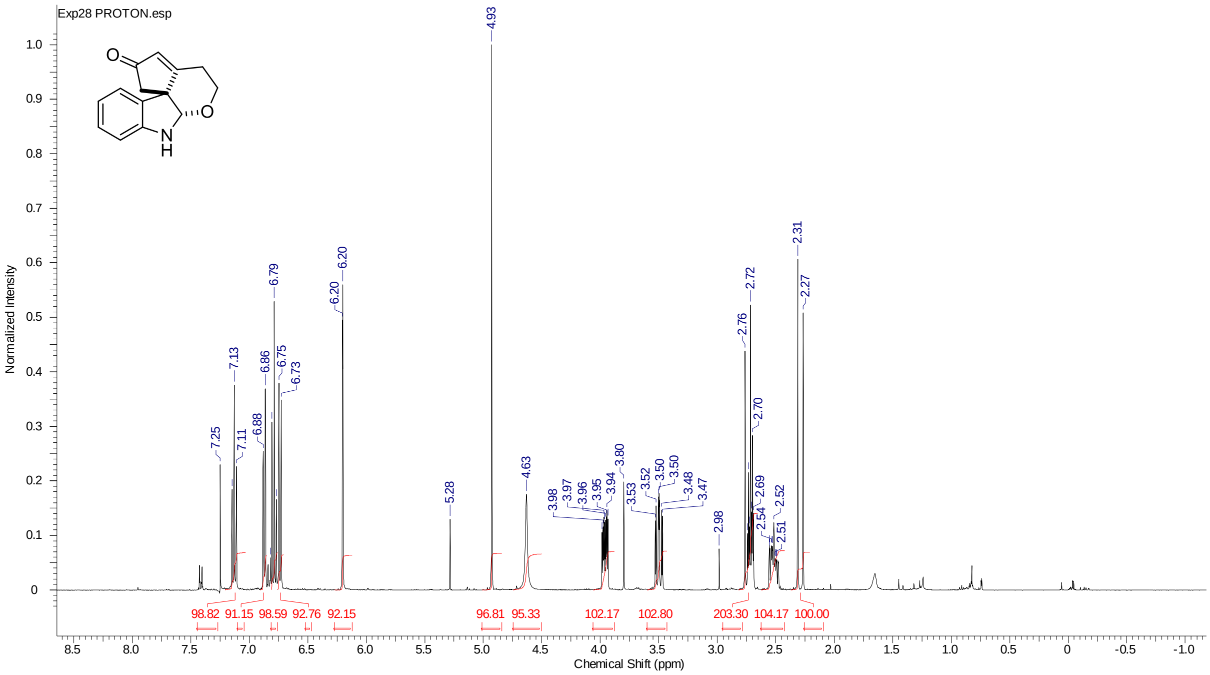
Exp28 PROTON.esp
Normalized Intensity
Chemical Shift (ppm)
4.93
6.20
6.20
6.79
7.13
6.86
6.75
6.73
7.25
7.11
6.88
5.28
4.63
3.98
3.97
3.96
3.95
3.94
3.80
3.53
3.52
3.50
3.50
3.48
3.47
2.98
2.76
2.72
2.70
2.69
2.54
2.52
2.51
2.31
2.27
98.82
91.15
98.59
92.76
92.15
96.81
95.33
102.17
102.80
203.30
104.17
100.00

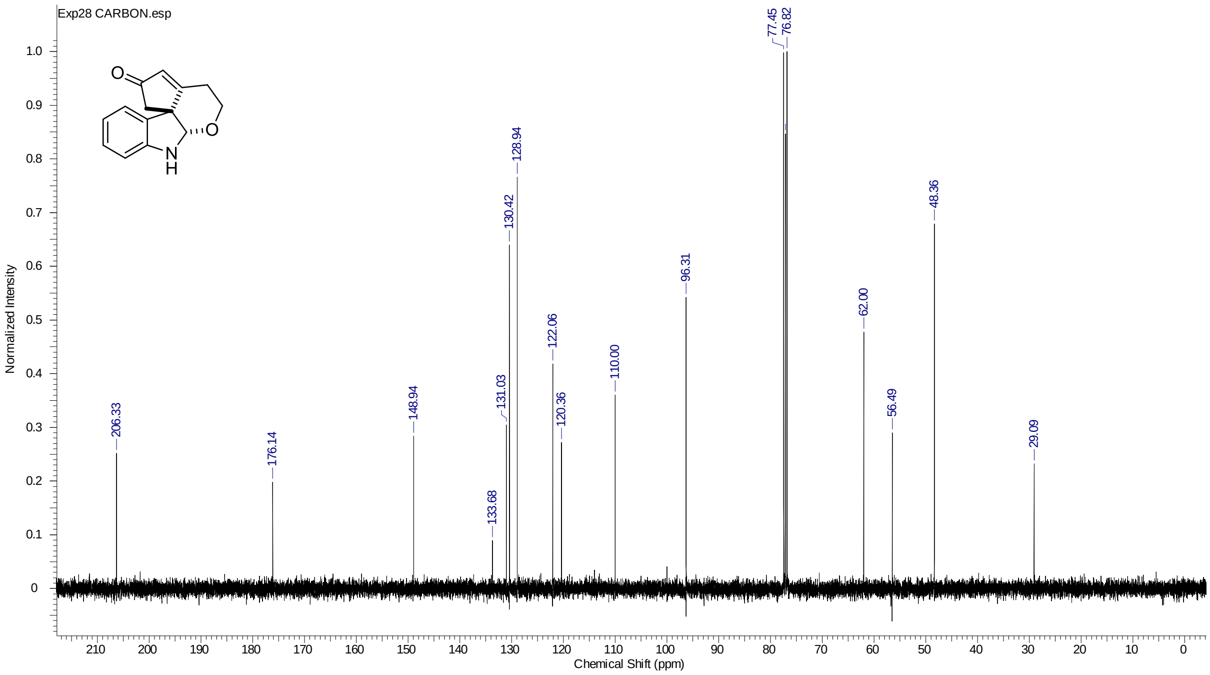
Exp28 CARBON.esp
Normalized Intensity
Chemical Shift (ppm)
77.45
76.82
128.94
48.36
130.42
96.31
62.00
122.06
110.00
56.49
148.94
131.03
120.36
206.33
29.09
176.14
133.68

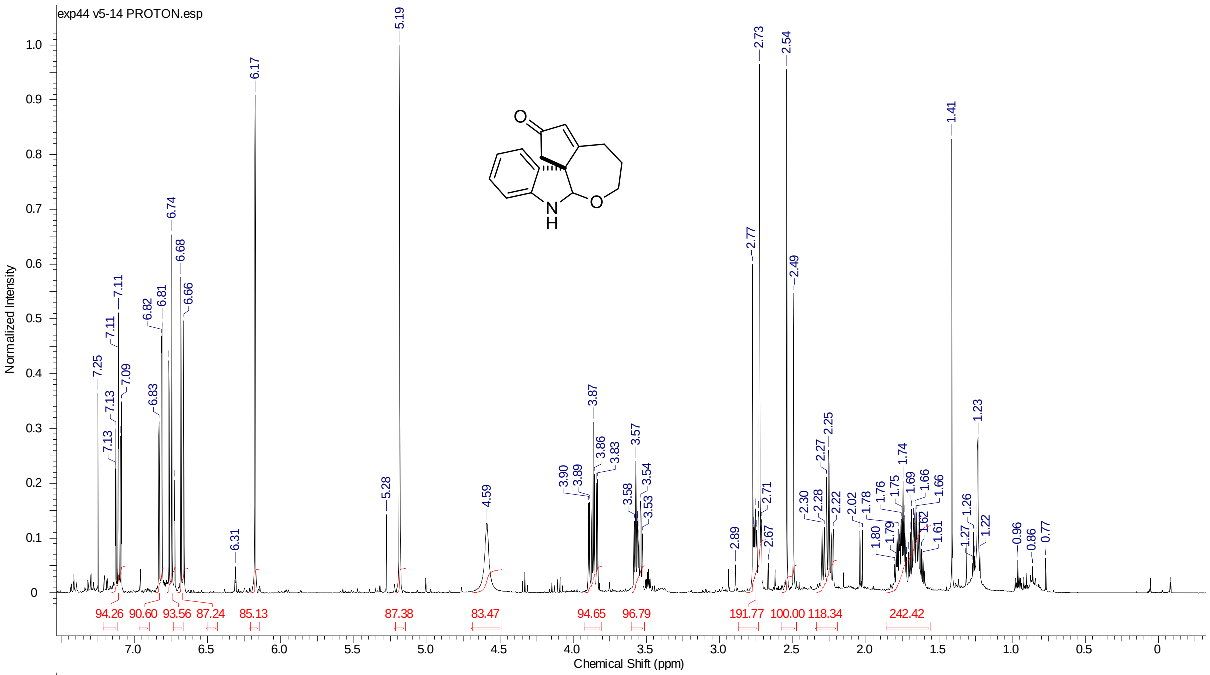
exp44 v5-14 PROTON.esp
Normalized Intensity
Chemical Shift (ppm)
O
N
H
O

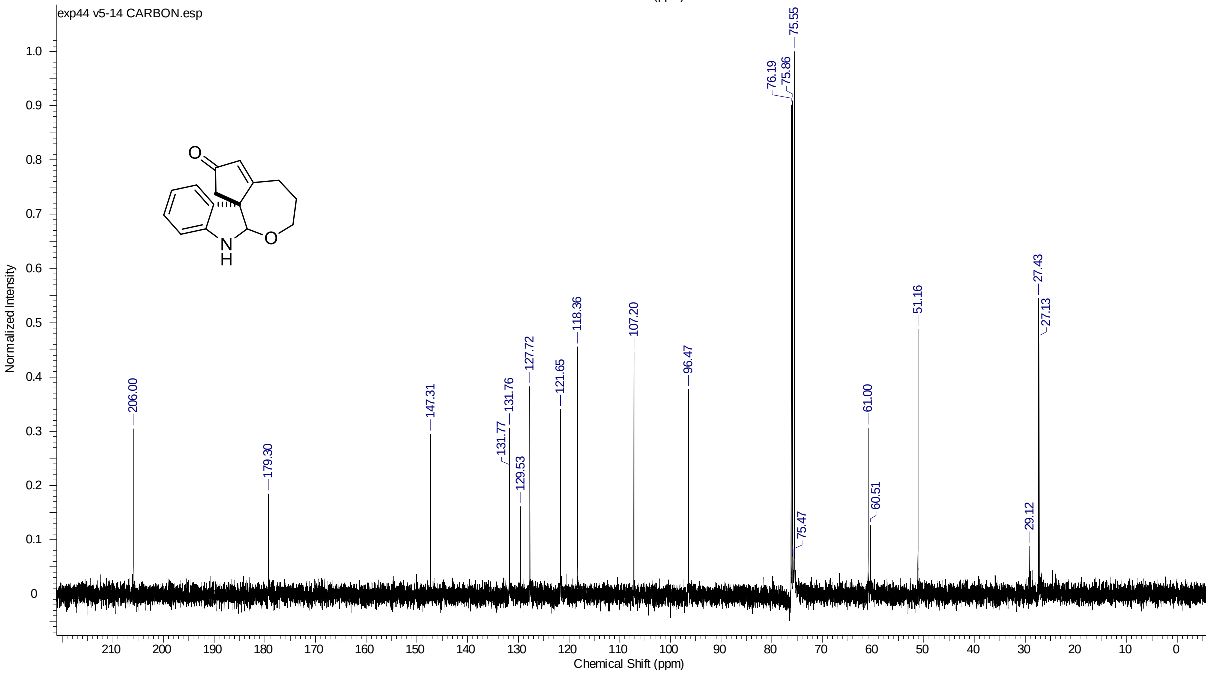
exp44 v5-14 CARBON.esp
Normalized Intensity
Chemical Shift (ppm)
O
N
H
O

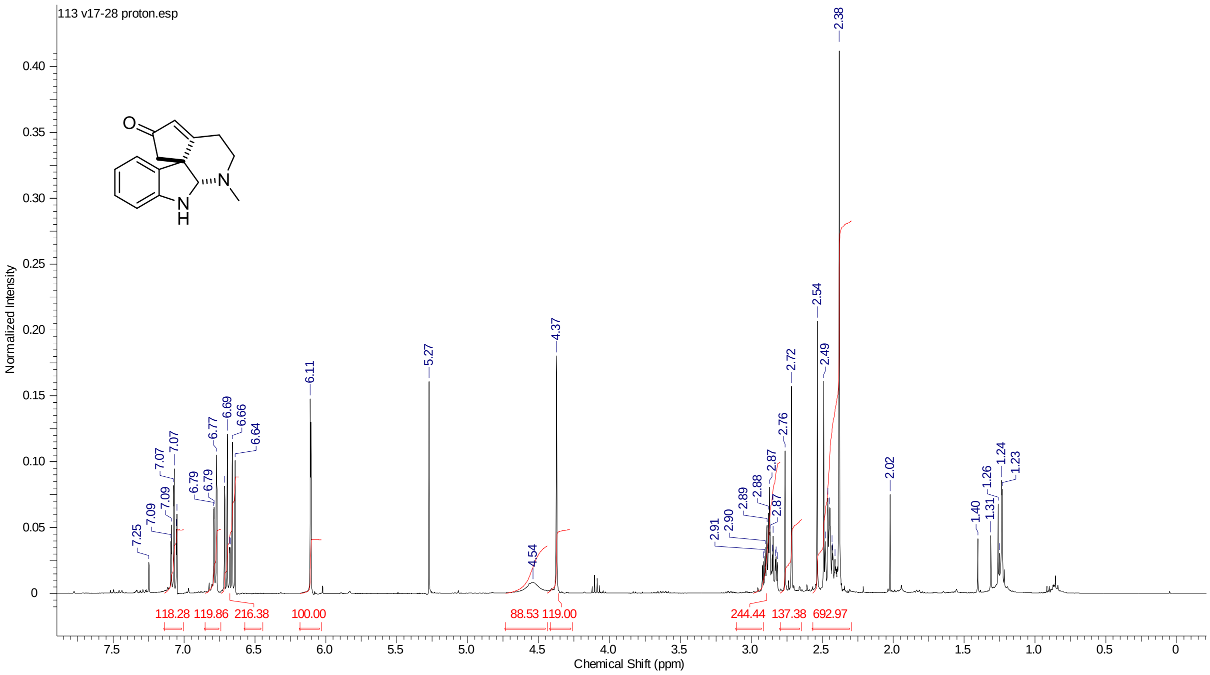
113 v17-28 proton.esp
Normalized Intensity
Chemical Shift (ppm)
2.38
2.54
2.49
2.72
2.76
4.37
4.54
5.27
6.11
7.25
7.09
7.07
7.09
7.07
6.79
6.79
6.77
6.69
6.66
6.64
2.91
2.90
2.89
2.88
2.87
2.87
2.02
1.40
1.31
1.26
1.24
1.23
118.28
119.86
216.38
100.00
88.53
119.00
244.44
137.38
692.97
O
N
H

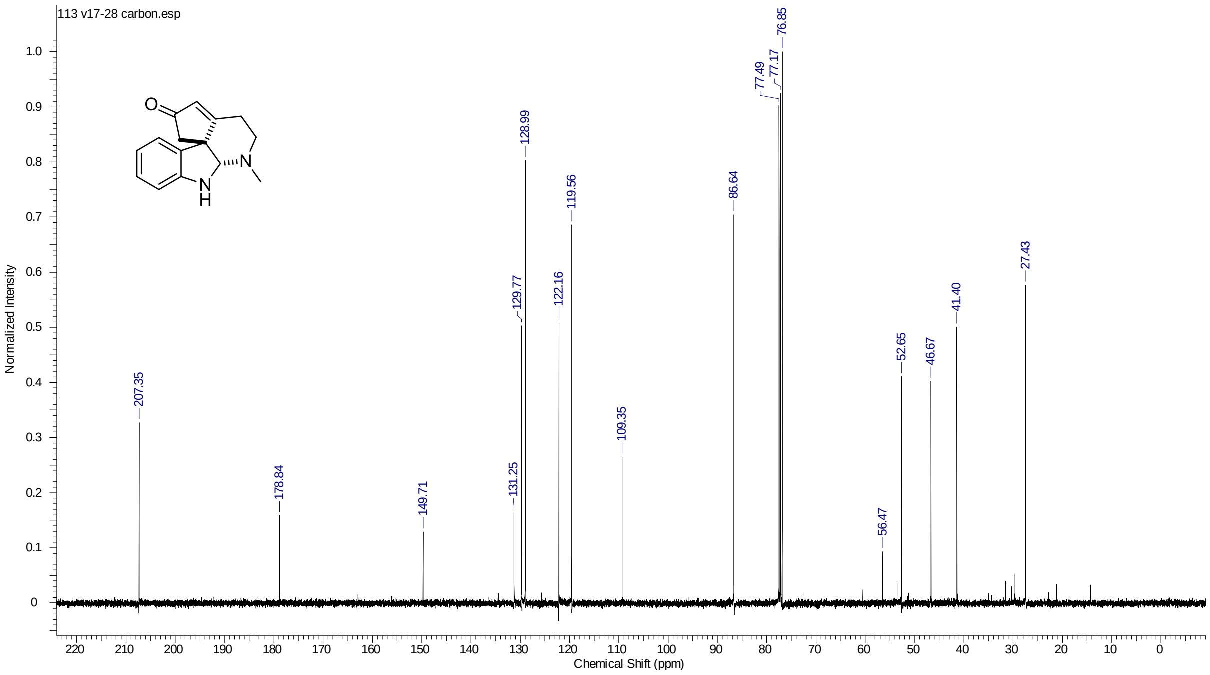
113 v17-28 carbon.esp
Normalized Intensity
Chemical Shift (ppm)
207.35
178.84
149.71
131.25
129.77
128.99
122.16
119.56
109.35
86.64
77.49
77.17
76.85
56.47
52.65
46.67
41.40
27.43
O
N
H

CSP-HPLC of **8h** using Chiralpak IB column, eluting with 10% IPA in hexanes

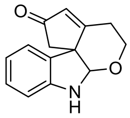

Racemic **8h** Recorded at 230 nm

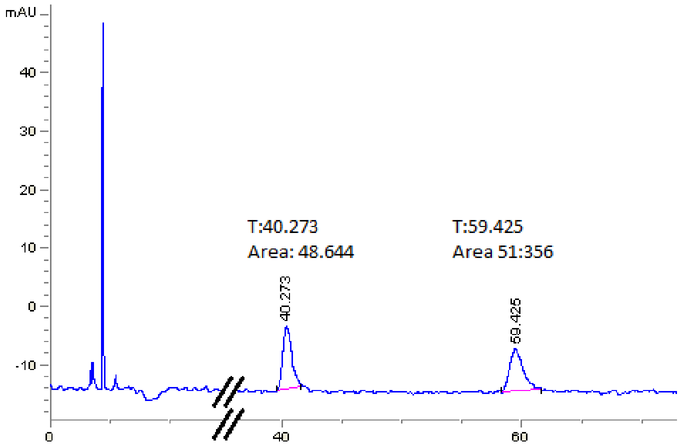

Enantioenriched **(*S*)-8h** Recorded at 230 nm

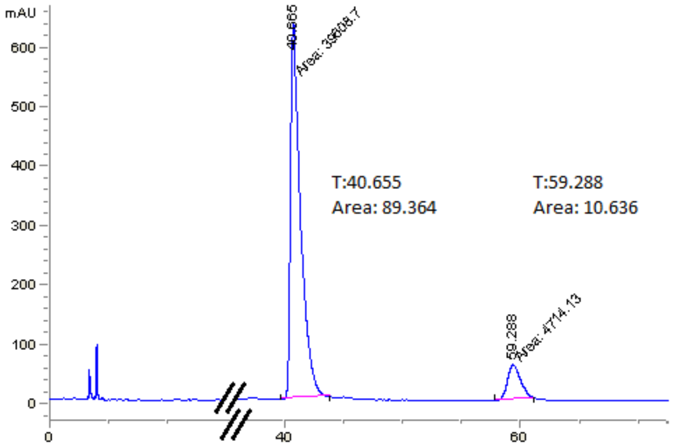

Recrystallized **(*S*)-8h** Recorded at 230 nm

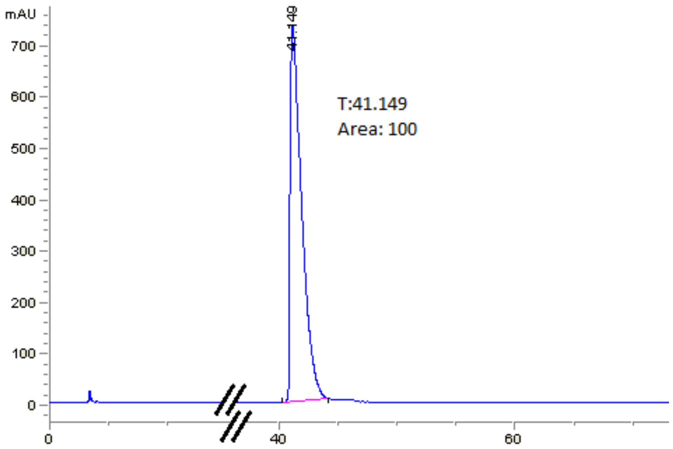

X-ray Crystal Images

CCDC 1453490 and 1453491 contain the supplementary crystallographic data for this paper. These data can be obtained free of charge from The Cambridge Crystallographic Data Centre via www.ccdc.com.ac.uk/data_request/cif

Compound **7f**

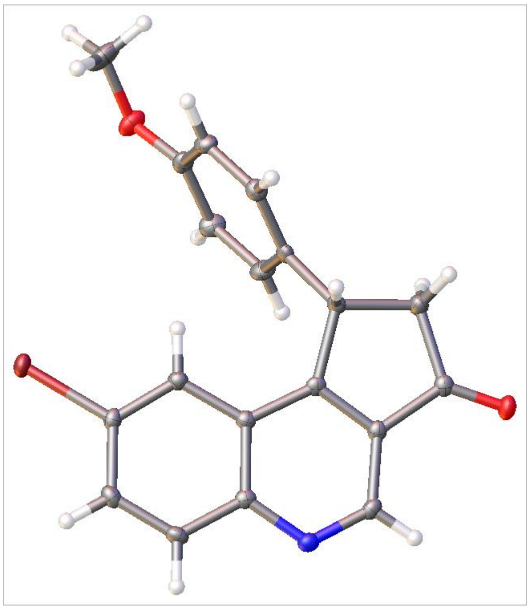

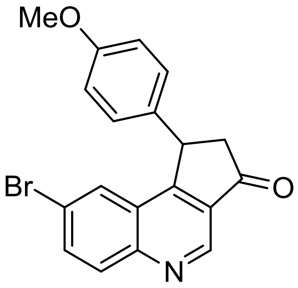

Compound ***(S)*-8h**

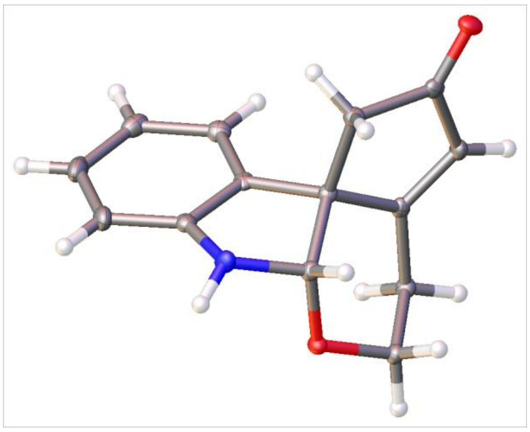

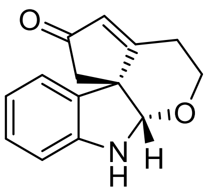

References

[1] M. J. James, J. D. Cuthbertson, P. O'Brien, R. J. K. Taylor, W. P. Unsworth, *Angew. Chemie Int. Ed.* **2015**, *54*, 7640–7643.

[2] M. J. James, R. E. Clubley, K. Y. Palate, T. J. Procter, A. C. Wyton, P. O'Brien, R. J. K. Taylor, W. P. Unsworth, *Org. Lett.* **2015**, *17*, 4372–4375.

[3] Z. Al-Shuhaib, H. Böckemeier, L. Coghlan, E. Dörksen, I. V. Jones, P. J. Murphy, R. Nash, J. M. Page, *Tetrahedron Lett.* **2013**, *54*, 6716–6718.

[4] B. Baire, D. Niu, P. H. Willoughby, B. P. Woods, T. R. Hoye, *Nat. Protoc.* **2013**, *8*, 501–8.

[5] M. Holmes, D. Kwon, M. Taron, R. Britton, *Org. Lett.* **2015**, *17*, 3868–3871.

[6] M. Wijtmans, D. a Pratt, J. Brinkhorst, R. Serwa, L. Valgimigli, G. F. Pedulli, N. a Porter, *J. Org. Chem* **2004**, *69*, 9215–9223.

[7] X.-L. Fang, R.-Y. Tang, P. Zhong, J.-H. Li, *Synthesis (Stuttg).* **2009**, *2009*, 4183–4189.
